# umite: fast quantification of Smart-seq3 libraries with improved UMI retrieval

**DOI:** 10.1093/bioinformatics/btag075

**Published:** 2026-02-15

**Authors:** Leo Carl Foerster, Enrico Frigoli, Xiaoyu Sun, Jooa Hooli, Angela Goncalves, Ana Martin-Villalba

**Affiliations:** Molecular Neurobiology, German Cancer Research Center (DKFZ), Heidelberg 69120, Germany; Combined Faculty of Mathematics, Engineering and Natural Sciences, University of Heidelberg, Heidelberg 69120, Germany; Molecular Neurobiology, German Cancer Research Center (DKFZ), Heidelberg 69120, Germany; Combined Faculty of Mathematics, Engineering and Natural Sciences, University of Heidelberg, Heidelberg 69120, Germany; Molecular Neurobiology, German Cancer Research Center (DKFZ), Heidelberg 69120, Germany; Combined Faculty of Mathematics, Engineering and Natural Sciences, University of Heidelberg, Heidelberg 69120, Germany; Molecular Neurobiology, German Cancer Research Center (DKFZ), Heidelberg 69120, Germany; Combined Faculty of Mathematics, Engineering and Natural Sciences, University of Heidelberg, Heidelberg 69120, Germany; Computational and Molecular Prevention, German Cancer Research Center (DKFZ), Heidelberg 69120, Germany; DKFZ Hector Cancer Institute, University Medicine Mannheim, Heidelberg 69120, Germany; Medical Faculty Mannheim, University of Heidelberg, Heidelberg 69120, Germany; Molecular Neurobiology, German Cancer Research Center (DKFZ), Heidelberg 69120, Germany

## Abstract

**Motivation:**

Commercial solutions like 10X cellranger provide robust UMI quantification for their proprietary single-cell protocols, but open methods such as Smart-seq3 lack comparable support.

**Results:**

Here, we introduce umite, a Smart-seq3 UMI counting pipeline with a focus on speed and a light memory footprint. Unlike existing tools, umite offers efficient mismatch-tolerant UMI detection, boosting UMI retrieval by 5%–15% in benchmarks. It also outperforms current Smart-seq3 quantification tools in runtime, disk usage, and memory footprint, offering better scalability on large datasets.

**Availability and implementation:**

umite is available at https://github.com/leoforster/umite (or via Zenodo: https://doi.org/10.5281/zenodo.18166431) and includes a Snakemake workflow for Smart-seq3 quantification.

## 1 Introduction

The advent of single-cell RNA-sequencing (scRNA-seq) technologies has had a transformative effect on molecular biology, enabling the rapid, high-throughput profiling of entire tissues and organs (Regev *et al.* 2017, The Tabula Muris Consortium *et al.* 2018, Cao *et al.* 2019). However, due to the low input volumes, stochastic biases in PCR during scRNA-seq library preparation can inordinately affect the resulting gene expression estimates (Parekh *et al.* 2016). To counteract this bias, unique molecular identifiers (UMIs) are added to individual transcripts prior to amplification, enabling the subsequent removal of PCR duplicates. This approach to deduplication results in more accurate differential gene expression estimates (Parekh *et al.* 2016) and has become ubiquitous across scRNA-seq protocols.

Although droplet-based and combinatorial indexing platforms dominate in the creation of large atlas-level datasets (Regev *et al.* 2017, The Tabula Muris Consortium *et al.* 2018, Cao *et al.* 2019), plate-based scRNA-seq protocols like Smart-seq3 remain relevant in experiments requiring higher transcriptome coverage (Hagemann-Jensen *et al.* 2020, Daugelaite *et al.* 2025), isoform resolution (Booeshaghi *et al.* 2021), or index sorted protein quantifications (Kremer *et al.* 2024, Foerster *et al.* 2025). While general purpose toolkits such as htseq-count (Anders *et al.* 2015) and UMI-tools (Smith *et al.* 2017) are able to handle UMI extraction and counting across protocols, to date zUMIs remains the standard and fastest pipeline for processing Smart-seq3 libraries (Parekh *et al.* 2018).

The zUMIs pipeline offers a flexible and highly configurable approach to Smart-seq3 quantifications which includes an innovative parallelization approach and separate tracking of intron- and exon-mapping reads (Parekh *et al.* 2018). However, zUMIs’ separate genome- and transcriptome-mapping steps inflate runtime, memory, and disk-space requirements, while its read processing and counting steps are prone to sharp memory spikes. Echoing this, the shell script architecture used by zUMIs limits extensibility and lacks robust error handling, causing friction for users debugging context-specific issues. Moreover, unlike the robust error-tolerant UMI detection implemented in UMI-tools, zUMIs uses position-based UMI detection which can miss reads with e.g. sequencing errors. To overcome these challenges, we developed umite, a unified Smart-seq3 quantification framework based on htseq-count, which supports customizable fuzzy UMI matching and consolidates UMI detection, correction, deduplication, and counting into a space- and time-efficient pipeline.

## 2 Features

**Figure 1:**
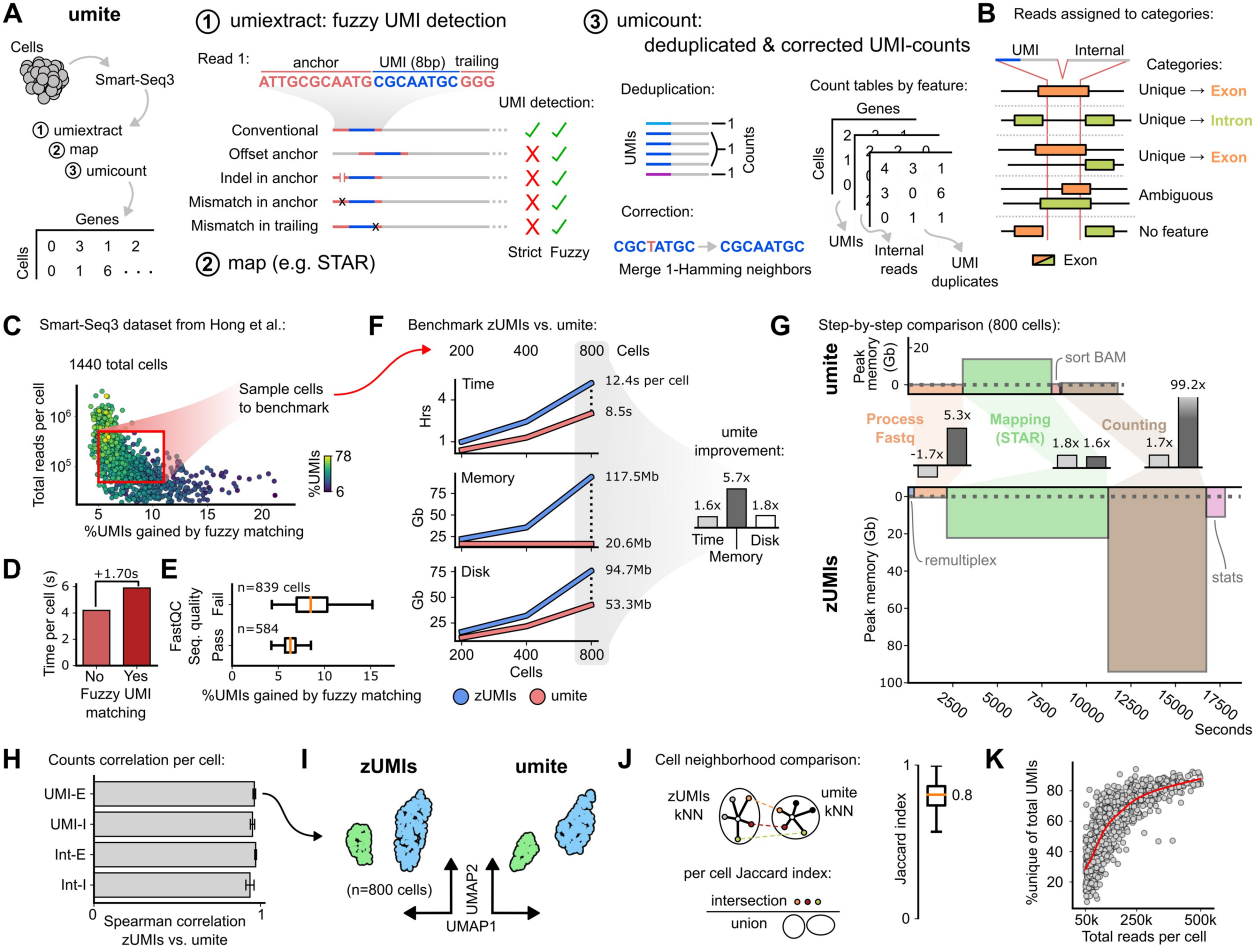
Quantification of Smart-seq3 libraries by umite. (A) Overview of the umite pipeline and individual stages of UMI extraction with fuzzy UMI detection as well as counting of deduplicated and corrected UMIs. (B) Categorization of aligned reads based on overlapping genomic features. (C) 1440 cells from Hong *et al*. dataset used for benchmarking. (D) Runtime increase incurred by enabling fuzzy UMI matching on cells in (C). (E) Relative UMIs gained through fuzzy matching, by FastQC sequence quality filter status. (F) Benchmarking results for zUMIs and umite pipelines on 200, 400, and 800 cell-samples from (C), in terms of time taken, peak memory, and used disk space. Usage units reflect average per cell. Barplot (right) represents resource usage fold-reduction for umite versus zUMIs. (G) Step-by-step comparison of 800-cell benchmark from (F) for umite (top) and zUMIs (bottom). Pipeline steps are matched by color. Inset barplots represent fold-reduction in resource usage per stage for umite versus zUMIs. (H) Mean Spearman correlation of UMI- and internal (Int)-reads in exonic (-E) and intronic (-I) categories, from 800-cell benchmark in (F). Correlations are averaged across cells, errorbars represent standard deviation. (I) UMAP embedding of UMI-E counts from zUMIs and umite. (J) Schematic of per-cell kNN Jaccard indexes (left), quantified boxplot for 800-cell benchmark (right). (K) Relationship between cell library size and percentage of unique versus duplicate UMIs.

The umite module comprises a pair of Python command line tools, umiextract and umicount (Fig. 1A), which orchestrate the following workflow: first, UMI containing reads are identified and tagged using umiextract. Then, reads are mapped to the genome using any standard RNA-seq aligner such as STAR (Dobin *et al.* 2013). The resulting BAM files are then input to umicount to generate counts matrices for deduplicated and corrected UMI- and internal-read counts, as well as for UMI duplicates. Both tools support parallel processing at the cell or library level, speeding up runtimes without additional overhead. Here, we provide details of the umite tools, followed by benchmarking against zUMIs. For additional details and in-depth descriptions of the underlying algorithms see the Supplement, available as supplementary data at *Bioinformatics* online.

In both umiextract and zUMIs, UMIs are detected from FASTQ reads via flanking sequences introduced during Smart-seq3 library preparation, including an 11 bp template switching oligo (TSO; ATTGCGCAATG, here called the “anchor”) and a GGG trinucleotide “trailing” sequence added during reverse transcription. The UMI itself consists of the bases between these anchor and trailing sequences, usually being 8 bp in length (Hagemann-Jensen *et al.* 2020). In zUMIs, UMI-containing reads are identified using a position-specific anchor-matching approach which tolerates limited mismatches. While such position-based approaches will correctly identify most UMIs, sequencing errors and other technical artifacts can obscure or displace the anchor, causing valid UMIs to be overlooked. To rescue such reads, umiextract leverages fuzzy (i.e. error-tolerant) string matching to recognize mutated or shifted anchor and trailing sequences, ensuring their proper retention. At the command line, umiextract offers flexible options for specifying UMI length as well as anchor and trailing sequences, including mismatch thresholds in fuzzy matching. Together, these features enable umiextract to recover more valid reads, offering greater flexibility without added complexity.

The umicount tool processes a BAM file and its corresponding GTF annotations to produce count tables of deduplicated and corrected UMI- and internal-reads. At its core, umicount adapts the main counting loop from htseq-count, where genomic features from the GTF file are represented by genomic intervals, enabling efficient querying of read alignment overlaps using set operations (Anders *et al.* 2015). Unlike zUMIs, which requires a secondary transcriptome alignment to capture intronic reads, umicount directly assigns such reads from existing gene- and exon-level GTF annotations. Counts are then determined based on the overlap between aligned reads and genomic features, with each read pair assigned to one of several categories (Fig. 1B): unmapped, if not aligned; no feature, if intergenic; multimapping, if repeated; ambiguous, if overlapping multiple genes; or unique, if overlapping a single gene. Unique reads are then aggregated per gene, and UMI correction carried out by merging 1-Hamming neighbors. To avoid overcorrection by chain-merging, UMIs are merged only if one was observed at least twice as frequently as the other, consistent with UMI-tools and zUMIs (Smith *et al.* 2017, Parekh *et al.* 2018). After correction, UMIs are deduplicated per gene. Gene counts for UMI-containing, internal, or UMI duplicate read pairs are tracked separately, as are those for exonic and intronic reads within each group. These counts are then output in separate counts matrices for downstream analysis. Overall, umicount combines accurate counting according to defined criteria with an efficient implementation and a low memory footprint.

## 3 Benchmarking

To evaluate the impact of fuzzy UMI matching and benchmark umite against zUMIs, we analyzed 1440 Smart-seq3 libraries from the murine nasal vasculature study by Hong *et al.* (Hong *et al.* 2023) (Fig. 1C). First, we ran FastQC to capture cells’ quality metrics, then extracted UMIs with umiextract both with and without fuzzy matching. Overall, cells contained 6%–78% UMI-reads and enabling fuzzy matching recovered an additional 5%–15% of UMIs at a cost of about 1.7 s per cell (Fig. 1D). Interestingly, cells failing FastQC thresholds tended to benefit more from fuzzy UMI matching, suggesting that lower library quality disproportionately impacts strict matching (Fig. 1E). Yet even among high-quality cells, fuzzy matching boosted UMI recovery by roughly 6%. As non-UMI reads are routinely discarded in downstream analysis, even a modest gain in UMI detection can impact gene expression estimates. We repeated this analysis on 500 human CD4+ T-cells from Chuang *et al.* (Chuang *et al.* 2024) (see Supplement, available as supplementary data at *Bioinformatics* online), observing a similar proportion of UMIs per cell (17%–64%) and a mean per-cell UMI gain of 13% despite this dataset exhibiting distinct sequence error profiles, highlighting the generality of the umite approach.

To compare runtimes and scaling of both umite and zUMIs pipelines, we next sampled 200, 400, and 800 nasal vasculature cells with replacement from the densest region of (Fig. 1C). Cells had between 50 000 and 500 000 reads, with the 800-cell sample comprising approximately 150 million paired reads. All benchmarks were run on eight cores (each a 2 GHz Intel Broadwell processor, see Supplement, available as supplementary data at *Bioinformatics* online), with running time and peak memory tracked using GNU time, and disk usage assessed after termination. Across every sample, umite outperformed zUMIs in all metrics (Fig. 1F). While resource usage for umite scaled largely linearly with increasing cell number, zUMIs’ memory usage in particular grew disproportionately, rising sharply for larger cell counts. Notably, on the 800-cell sample, umite achieved a 31% (1.6-fold) reduction in overall running time, an 82% (5.7-fold) reduction in peak memory, and a 43% (1.8-fold) reduction in disk space. Comparing the tools’ performance step-by-step (Fig. 1G), we noted that while FASTQ processing (i.e. read QC & UMI detection) took about 40% (1.7-fold) longer due to having fuzzy UMI matching enabled, the overall umite memory footprint remained approximately 80% (5.3-fold) lower. Despite using the same STAR parameters, umite performed better during the mapping step due to the dual genome and transcriptome mapping carried out by zUMIs. Finally, at the counting step (which includes UMI deduplication & correction), umite again demonstrated improved runtimes (by 40%, 1.7-fold) and memory footprint (by 99%, 99.2-fold) over zUMIs. In sum, umite delivers more efficient and better scaling Smart-seq3 quantification compared to zUMIs, while enabling improved UMI detection through fuzzy UMI matching.

We additionally compared counts estimates between umite and zUMIs for the 800-cell sample. To match zUMIs counting logic, we enabled primary alignment counting for multimapping reads in umicount. Both tools produce separate UMI- and internal-read counts, each further divided into exonic and intronic tables. Comparing per-cell expression by Spearman correlation showed consistently high agreement between the two methods (Fig. 1H). When we applied identical preprocessing to the exonic UMI counts matrices, the resulting UMAPs (McInnes *et al.* 2020) were near-identical and recapitulated those in the original study (Hong *et al.* 2023) (Fig. 1I). To quantify local neighborhood similarity, we computed the Jaccard index on each cell’s kNN graph, observing consistently high overlap with a mean of 0.8 across cells (Fig. 1J). Given this consistent agreement in gene expression estimates, we conclude that the improved speed and efficiency of umite do not compromise its accuracy.

## 4 Discussion

Smart-seq3 remains a popular choice in single-cell transcriptomics, with recent advances in miniaturization and integration of multi-omic readouts (Cerrizuela *et al.* 2022) ensuring its continued application. To streamline Smart-seq3 data processing, we developed umite, a flexible and efficient pipeline which combines features from related tools, including separate counting of intronic and exonic reads as in zUMIs (Parekh *et al.* 2018), as well as enabling fuzzy UMI matching and frequency-aware UMI correction as in UMI-tools (Smith *et al.* 2017). Our results indicate that, compared to zUMIs, umite achieves accurate quantification of gene expression with considerably lower time, space, and memory requirements. Furthermore, by implementing robust error-tolerant UMI detection umite achieves a 5%–15% gain in UMI-containing reads. Uniquely, umite reports per-gene UMI duplicate counts, enabling saturation analyses (Fig. 1K) and systematic assessment of PCR bias (e.g. by gene GC content). While umite implements cell-level multithreading, runtimes can become skewed when some libraries are significantly larger than others. In future versions, we may consider reducing runtimes through combined, alignment-free UMI detection and deduplication during preprocessing (Orabi *et al.* 2019, Tsagiopoulou *et al.* 2021). Overall, umite provides a lightweight pipeline for Smart-seq3 quantification, with an integrated Snakemake workflow that enables improved UMI detection and faster processing without compromising accuracy.

## Supplementary Material

btag075_Supplementary_Data

## Data Availability

umite is available at https://github.com/leoforster/umite (or via Zenodo: https://doi.org/10.5281/zenodo.18166431). Single cell libraries of the mouse nasal vasculature dataset (GSE207085) and human CD4+ T-cell dataset (GSE270928) used in benchmarking were downloaded from NCBI. Notebooks documenting data processing and benchmarking analyses are deposited in Zenodo and uploaded as supplementary data at *Bioinformatics* online.
