# Supplementary material for: umite: fast quantification of Smart-seq3 libraries with improved UMI retrieval
Source: Bioinformatics. 2026 Feb 15;42(3):btag075. doi: 10.1093/bioinformatics/btag075 (PMC12989134; doi:10.1093/bioinformatics/btag075)
Supplement: btag075_Supplementary_Data [file btag075_supplementary_data.zip › umite_pipeline_benchmarks_and_figures_documentation.html]

umite\_pipeline\_benchmarks\_and\_figures\_documentation


# umite pipeline benchmarks and figures documentation¶

this notebook serves to document the datasets and analysis which comprise the article introducing umite (https://github.com/leoforster/umite)

In [ ]:

```

```

## dataset download¶

in finding an appropriate benchmarking dataset, the primary focus was to identify a recent publication with a large number of smart-seq3 cells available on GEO. Here, we use the data published with:

Hong, S.P., Yang, M.J., Bae, J.H. et al. Three-dimensional morphologic and molecular atlases of nasal vasculature. Nat Cardiovasc Res 2, 449–466 (2023). https://doi.org/10.1038/s44161-023-00257-3

In [ ]:

```

```

next we need to download the raw fastqs from the sequence read archive (SRA), using the sources as identified in the corresponding GEO source: https://www.ncbi.nlm.nih.gov/geo/query/acc.cgi?acc=GSE207085

we retrieve the accessions for the individual cell libraries manually using the SRA run selector (accessible from the GEO source, see below), and export said accessions into a file called accessions\_full.sort.txt

In [ ]:

```

```

### from SRA¶

using SRA accessions from the accessions list, we use the SRA prefetch tool to begin downloading these to disk

In [ ]:

```
cd /mnt/volume/resources/external_SS3_cells/2nd_nasal_dataset_complete_bench
$CO/sratoolkit.2.10.5-ubuntu64/bin/prefetch --option-file accessions_full.sort.txt
```

In [ ]:

```

```

In [ ]:

```
# check all files were downloaded
ls -1 -d */ | rev | cut -c 2- | rev | sort > accesions_downloaded.txt
diff -b accesions_downloaded.txt accessions_full.sort.txt | grep '^>' | cut -c 3- > accessions_missed.txt
```

In [ ]:

```
$CO/sratoolkit.2.10.5-ubuntu64/bin/prefetch --option-file accessions_missed.txt
```

In [ ]:

```

```

convert to fastq with --origfmt (necessary for zUMIs)

In [ ]:

```
find ./ -name '*.sra' | xargs -I % echo /home/ubuntu/dev/co/sratoolkit.2.10.5-ubuntu64/bin/fastq-dump --origfmt --defline-qual '+' --gzip --split-files % > SRA_fastq_dump_calls.sh
```

In [ ]:

```
./SRA_fastq_dump_calls.sh
```

In [ ]:

```
find ./ -type d | xargs -I % rm -r %
```

In [ ]:

```

```

## dataset preprocessing¶

now that we have the fastq libraries, its time to inspect the data quality

In [ ]:

```

```

### fastqc¶

run fastqc

In [ ]:

```
cat accessions_full.sort.txt | xargs -I % echo "fastqc -f fastq -t 6 -o ./fastq_reads_fastqc/ %_1.fastq.gz %_2.fastq.gz" > sra_accessions.fastqc.sh
```

In [ ]:

```
parallel ./sra_accessions.fastqc.sh
```

In [ ]:

```

```

then collate using multiqc

In [ ]:

```
fastqc fastqs/*.fastq.gz
multiqc fastqs/
```

In [ ]:

```

```

In [ ]:

```

```

In [ ]:

```

```

store the multiQC output in a dataframe, for use in downstream filtering of cells

In [15]:

```
mqcdata = pd.read_csv('/mnt/volume/jupyter/projects/manuscript_prep/11_umicounts_benchmarking/XX_input/bench_data_nasopharyngeal/multiqc_data/multiqc_fastqc.txt', sep='\t')
```

In [16]:

```
mqcdata.columns
```

Out[16]:

```
Index(['Sample', 'Filename', 'File type', 'Encoding', 'Total Sequences',
       'Sequences flagged as poor quality', 'Sequence length', '%GC',
       'total_deduplicated_percentage', 'avg_sequence_length',
       'median_sequence_length', 'basic_statistics',
       'per_base_sequence_quality', 'per_tile_sequence_quality',
       'per_sequence_quality_scores', 'per_base_sequence_content',
       'per_sequence_gc_content', 'per_base_n_content',
       'sequence_length_distribution', 'sequence_duplication_levels',
       'overrepresented_sequences', 'adapter_content', 'kmer_content'],
      dtype='object')
```

In [ ]:

```

```

## modified zUMIs and umite for benchmarking¶

the zUMIs and umite pipelines documented below were each run identically for 200/400/800 cell sets as demonstrated in the following sections. the code here is provided to document the benchmarking process and pipeline execution.

In [ ]:

```

```

### genome index¶

as both tools use the same index, we dont add the index creation to the runtime/memory benchmarks

In [ ]:

```
git clone https://github.com/alexdobin/STAR.git
```

In [ ]:

```
/home/l329r/scratch/leo/STAR/bin/Linux_x86_64/STAR --runThreadN 16 \
     --runMode genomeGenerate \
     --genomeDir /home/l329r/scratch/leo/reference/star_mouse_GRCm38_reference \
     --genomeFastaFiles /home/l329r/scratch/leo/reference/Mus_musculus.GRCm38.dna_rm.primary_assembly.fa \
     --sjdbGTFfile /home/l329r/scratch/leo/reference/Mus_musculus.GRCm38.96.gtf \
     --sjdbOverhang 99
```

In [ ]:

```

```

### setup zUMIs¶

the current version of zUMIs (2.9.7e) merges all execution steps into a monolithic zUMIs.sh . to enable benchmarking, we will use a wrapper function which reproduces the original calls within zUMIs.sh but pipes them through gnu time to track time and memory usage.

it is also necessary to un-demultiplex (henceforth remultiplex) fastq files retrieved from SRA, as un-demultiplexed reads are the default input for zUMIs

In [ ]:

```

```

#### remultiplex¶

since zUMIs expects non-demultiplexed input we use the helpfully provided script:

In [ ]:

```
f(!require(stringi)){
  install.packages("stringi")
  library(stringi)
}

if(!require(optparse)){
  install.packages("optparse")
  library(optparse)
}

if(!require(data.table)){
  install.packages("data.table")
  library(data.table)
}

setDTthreads(1)

option_list <- list(
  make_option(c("-d", "--dir"), type="character",
              help="Directory with fastq files. Mandatory"),
  make_option(c("-p", "--pigz"), type="character",
              help="Executable for pigz. Default: pigz.", default = "pigz"),
  make_option(c("-t","--threads"), type="integer",
                help="Number of threads to use. Default: 24",
              default=24)
)
opt <- parse_args(OptionParser(option_list=option_list))

fastq_directory <- opt$dir
fastq_files <- list.files(path = fastq_directory, pattern = ".[fastq|fq].gz$")


### first check if the fastq file names are bcl2fastq style or SRA style:
num_files_sra <- sum(grepl(pattern = '_[1-2].nonsense', fastq_files))
num_files_bcl <- sum(grepl(pattern = '_R[1-2].fastq.gz', fastq_files)) # modified

if(num_files_bcl >= num_files_sra){
  style <- 'bcl2fastq'
  file_delim_r1 <- "_R1"
  file_delim_r2 <- "_R2"
}else{
  style <- 'SRA'
  file_delim_r1 <- "_R1.fastq.gz"
  file_delim_r2 <- "_R2.fastq.gz"
}
print(paste("Detected files to be in", style, "format."))


read1_files <- grep(file_delim_r1, fastq_files, value = TRUE)
read2_files <- grep(file_delim_r2, fastq_files, value = TRUE)


#check if SE or PE data
if(length(read2_files) == 0){
  mode <- "SE"
}else{
  mode <- "PE"
}

#terminate if there is no data!
if(length(read1_files) == 0){
  print("No valid fastq files found!")
  stop()
}

samples <- data.table(r1 = read1_files)
if(mode == "PE") samples[,r2 := read2_files]
samples[, sample := tstrsplit(r1, file_delim_r1, keep = 1)][
        , BC := stringi::stri_rand_strings(.N, 8, pattern = "[A-Z]")]

outfile_r1 <- paste0(opt$dir,"/reads_for_zUMIs.R1.fastq.gz")
outfile_r2 <- paste0(opt$dir,"/reads_for_zUMIs.R2.fastq.gz")
outfile_index <- paste0(opt$dir,"/reads_for_zUMIs.index.fastq.gz")

for(i in seq(nrow(samples))){
  system(paste("cat", paste(opt$dir,samples[i]$r1,sep = "/"), ">>", outfile_r1))
  if(mode == "PE") system(paste("cat", paste(opt$dir,samples[i]$r2,sep = "/"), ">>", outfile_r2))
  system(paste0(opt$pigz," -p2 -dc ", paste(opt$dir,samples[i]$r1,sep = "/"), " | awk -v bc=\"",samples[i]$BC,"\" '{i++;if(i==1 || i==3){print;}if(i==2){print bc;}if(i==4){i=0;print \"AAAAAAAA\";}}' | ",opt$pigz," -c -p",opt$threads," >> ",outfile_index))
}

#system(paste(opt$pigz,"-p",opt$threads,outfile_r1))
#if(mode == "PE") system(paste(opt$pigz,"-p",opt$threads,outfile_r2))
#system(paste(opt$pigz,"-p",opt$threads,outfile_index))

fwrite(samples, file =  paste0(opt$dir,"/reads_for_zUMIs.samples.txt"), quote = F, sep = "\t")
write(samples$BC, file = paste0(opt$dir,"/reads_for_zUMIs.expected_barcodes.txt"))
```

In [ ]:

```

```

and benchmark run by pointing to directory with files

In [ ]:

```
# taskset limits available cores
taskset -c 0-7 /usr/bin/time -v Rscript remultiplex.R -d fastqs_multiqc_200cell/ -p /usr/bin/pigz -t 8
```

In [ ]:

```

```

example output:

In [ ]:

```
	Command being timed: "Rscript remultiplex.R -d fastqs_multiqc_200cell/ -p /usr/bin/pigz -t 8"
	User time (seconds): 356.82
	System time (seconds): 112.22
	Percent of CPU this job got: 207%
	Elapsed (wall clock) time (h:mm:ss or m:ss): 3:45.96
	Average shared text size (kbytes): 0
	Average unshared data size (kbytes): 0
	Average stack size (kbytes): 0
	Average total size (kbytes): 0
	Maximum resident set size (kbytes): 81500
	Average resident set size (kbytes): 0
	Major (requiring I/O) page faults: 126
	Minor (reclaiming a frame) page faults: 529013
	Voluntary context switches: 11698977
	Involuntary context switches: 2824930
	Swaps: 0
	File system inputs: 24636168
	File system outputs: 31749264
	Socket messages sent: 0
	Socket messages received: 0
	Signals delivered: 0
	Page size (bytes): 4096
	Exit status: 0
```

In [ ]:

```
-rw-r--r-- 1 root root 7.0G Apr 16 12:42 fastqs_multiqc_200cell/reads_for_zUMIs.R1.fastq.gz
-rw-r--r-- 1 root root 7.7G Apr 16 12:42 fastqs_multiqc_200cell/reads_for_zUMIs.R2.fastq.gz
-rw-r--r-- 1 root root 2.2K Apr 16 12:42 fastqs_multiqc_200cell/reads_for_zUMIs.expected_barcodes.txt
-rw-r--r-- 1 root root 554M Apr 16 12:42 fastqs_multiqc_200cell/reads_for_zUMIs.index.fastq.gz
-rw-r--r-- 1 root root  17K Apr 16 12:42 fastqs_multiqc_200cell/reads_for_zUMIs.samples.txt
```

In [ ]:

```

```

#### zUMIs yaml¶

here we document the zUMIs yaml file, in this case for the 200 cells run, however the yaml files used in the 400 and 800 cell runs are effectively identical and just point to different folders for the inputs

In [ ]:

```
project: zumis_nasopharyngeal_200cell
sequence_files:
  file1:
    name: /mnt/scratch/leo/zumis_fastqs/reads_for_zUMIs.R1.fastq.gz
    base_definition:
      - cDNA(23-151)
      - UMI(12-19)
    find_pattern: ATTGCGCAATG
  file2:
    name: /mnt/scratch/leo/zumis_fastqs/reads_for_zUMIs.R2.fastq.gz
    base_definition:
      - cDNA(1-151)
  file3:
    name: /mnt/scratch/leo/zumis_fastqs/reads_for_zUMIs.index.fastq.gz
    base_definition:
      - BC(1-8)
reference:
  STAR_index: /mnt/scratch/leo/reference/star_mouse_GRCm38_reference_150bp_overhang/
  GTF_file: /mnt/scratch/leo/reference/Mus_musculus.GRCm38.96.gtf
  additional_STAR_params: '--clip3pAdapterSeq CTGTCTCTTATACACATCT' 
  additional_files:
out_dir: /mnt/scratch/leo/zumis_run/zumis_200cell_output
num_threads: 8
mem_limit: 99
filter_cutoffs:
  BC_filter:
    num_bases: 3
    phred: 20
  UMI_filter:
    num_bases: 2
    phred: 20
barcodes:
  barcode_num: ~
  barcode_file: /mnt/scratch/leo/zumis_fastqs/reads_for_zUMIs.expected_barcodes.txt
  automatic: no
  BarcodeBinning: 0
  nReadsperCell: 100
  demultiplex: no
counting_opts:
  introns: yes
  downsampling: '0'
  strand: 0
  Ham_Dist: 1
  write_ham: no
  velocyto: no
  primaryHit: yes
  twoPass: no
make_stats: yes
which_Stage: Filtering
samtools_exec: /dev/null
pigz_exec: /dev/null
STAR_exec: /mnt/scratch/tools/STAR-2.7.3a/bin/Linux_x86_64/STAR
Rscript_exec: /dev/null
zUMIs_directory: /mnt/scratch/leo/zUMIs
read_layout: PE
```

In [ ]:

```

```

#### modify zumis.sh to report timing¶

we modify the central zumis.sh to add calls to date and report progress to stdout, accomplished by wrapping function calls to this benchmarking function:

In [ ]:

```
# Benchmarking function
benchmark() {
    local label="$1"
    shift
    echo "Running step: $label"
    start_time=$(date +%s)

    /usr/bin/time -v "$@" 2> "${outdir}/${project}.${label}.benchmark.log"

    end_time=$(date +%s)
    duration=$((end_time - start_time))
    echo "Step '$label' took ${duration} seconds" | tee -a "${outdir}/${project}.zUMIs_timing_summary.txt"
}
```

In [ ]:

```

```

this benchmark function was included in zUMIs.sh and edits made to wrap pipeline steps to wrap them via this function. Here, we reproduce the modified zUMIs.sh which was used to carry out benchmarks reported in the manuscript (the first benchmark call appears line ~200):

In [ ]:

```
#!/bin/bash

date

> "/mnt/scratch/leo/zumis_run/zUMIs.timing_summary.txt"

# Benchmarking function
benchmark() {
    local label="$1"
    shift
    echo "Running step: $label"
    start_time=$(date +%s)

    /usr/bin/time -v "$@" 2> "/mnt/scratch/leo/zumis_run/zUMIs.${label}.benchmark.log"

    end_time=$(date +%s)
    duration=$((end_time - start_time))
    echo "Step '$label' took ${duration} seconds" | tee -a "/mnt/scratch/leo/zumis_run/zUMIs.timing_summary.txt"
}

vers=2.9.7e
currentv=$(curl -s https://raw.githubusercontent.com/sdparekh/zUMIs/main/zUMIs.sh | grep '^vers=' | cut -f2 -d "=")
if [ "$currentv" != "$vers" ] ; then
    echo -e "------------- \n\n Good news! A newer version of zUMIs is available at https://github.com/sdparekh/zUMIs \n\n-------------";
fi

function check_opts() {
    value=$1
    name=$2
    flag=$3

    if [[ -z "${value}" ]] ; then
        failure "No ${name}!! One can not run this pipeline without ${flag} option."
    fi
}

function failure() {
	echo -e "\n There seems to be a problem. Please check the usage: \n $1 \n\n"
	usage
	exit 1
}

zumis=$0

function usage () {
    cat >&2 <<EOF

  USAGE: ${zumis} [options]
	-h  Print the usage info.

## Required parameters ##

	-y  <YAML config file> : Path to the YAML config file. Required.

## Program path ##
	-d  <zUMIs-dir>   	 : Directory containing zUMIs scripts.  Default: path to this script.

## Miniconda environment

  -c : Use zUMIs dependencies in the preinstalled conda enviroment.

zUMIs version ${vers}

EOF
}

# Define the default variables #
zumisdir=$(dirname $(readlink -f $0))

while getopts ":y:d:ch" options; do #Putting <:> between keys implies that they can not be called without an argument.
  case ${options} in
  y ) yaml=${OPTARG};;
  d ) zumisdir=${OPTARG};;
  c ) conda=true;;
  h ) usage
          exit 1;;
  \? ) echo -e "\n This key is not available! Please check the usage again: -${OPTARG}"
  	usage
  	exit 1;;
  esac
done

if [[ ${OPTIND} -eq 1 ]] ; then
    usage
    exit 1
fi

check_opts "${yaml}" "YAML" "-y"

# create temporary YAML file for corrected options
yaml_orig=${yaml}
yaml=$(dirname ${yaml})/$(basename ${yaml} .yaml).run.yaml
cp ${yaml_orig} ${yaml}

#now get some variables from YAML
num_threads=$(grep 'num_threads' ${yaml} | awk '{print $2}')
project=$(grep 'project:' ${yaml} | awk '{print $2}')
whichStage=$(grep 'which_Stage:' ${yaml} | awk '{print $2}')
outdir=$(grep 'out_dir' ${yaml} | awk '{print $2}')
genomedir=$(grep 'STAR_index:' ${yaml} | awk '{print $2}')
mem_limit=$(grep 'mem_limit:' ${yaml} | awk '{print $2}')
isstats=$(grep 'make_stats:' ${yaml} | awk '{print $2}')
fqfiles=$(grep 'name:' ${yaml} | awk '{print $2}')
velo=$(grep 'velocyto:' ${yaml} | awk '{print $2}')
staridxdir=$(grep 'STAR_index:' ${yaml} | awk '{print $2}')

if grep -q 'samtools_exec:' ${yaml} ; then
    samtoolsexc=$(grep 'samtools_exec' ${yaml} | awk '{print $2}')
else
    samtoolsexc=samtools
    echo "samtools_exec: ${samtoolsexc}" >> ${yaml}
fi

if grep -q 'pigz_exec:' ${yaml} ; then
    pigzexc=$(grep 'pigz_exec' ${yaml} | awk '{print $2}')
else
    echo "Warning: YAML file doesn't include 'pigz_exec' option; setting to 'pigz'"
    pigzexc=pigz
    echo "pigz_exec: ${pigzexc}" >> ${yaml}
fi

if grep -q 'STAR_exec:' ${yaml} ; then
    starexc=$(grep 'STAR_exec' ${yaml} | awk '{print $2}')
else
    echo "Warning: YAML file doesn't include 'STAR_exec' option; setting to 'STAR'"
    starexc=STAR
    echo "STAR_exec: ${starexc}" >> ${yaml}
fi

if grep -q 'Rscript_exec:' ${yaml} ; then
    Rexc=$(grep 'Rscript_exec' ${yaml} | awk '{print $2}')
else
    echo "Warning: YAML file doesn't include 'Rscript_exec' option; setting to 'Rscript'"
    Rexc=Rscript
    echo "Rscript_exec: ${Rexc}" >> ${yaml}
fi

#check for conda usage!
if [[ ${conda} = true ]] ; then
  echo "Using miniconda environment for zUMIs!"
  echo " note: internal executables will be used instead of those specified in the YAML file!"
  samtoolsexc=samtools
  if grep -q 'samtools_exec:' ${yaml} ; then
      sed -i '/samtools_exec:/d' ${yaml}
  fi
  echo "samtools_exec: ${samtoolsexc}" >> ${yaml}
  pigzexc=pigz
  if grep -q 'pigz_exec:' ${yaml} ; then
      sed -i '/pigz_exec:/d' ${yaml}
  fi
  echo "pigz_exec: ${pigzexc}" >> ${yaml}
  starexc=STAR
  if grep -q 'STAR_exec:' ${yaml} ; then
      sed -i '/STAR_exec:/d' ${yaml}
  fi
  echo "STAR_exec: ${starexc}" >> ${yaml}
  Rexc=Rscript
  if grep -q 'Rscript_exec:' ${yaml} ; then
      sed -i '/Rscript_exec:/d' ${yaml}
  fi
  echo "Rscript_exec: ${Rexc}" >> ${yaml}

  zumisenv=${zumisdir}/zUMIs-env
  miniconda=${zumisdir}/zUMIs-miniconda.tar.bz2
  #check if zUMIs environment has been unpacked from tar
  if [[ ! -d ${zumisenv} ]] || [[ ${zumisdir}/zUMIs-miniconda.partaa -nt ${zumisenv} ]] ; then
    [ -d ${zumisenv} ] || mkdir -p ${zumisenv}
    cat ${zumisdir}/zUMIs-miniconda.parta* > ${miniconda}
    tar -xj --overwrite -f ${miniconda} -C ${zumisenv}
  fi
  #activate zUMIs environment!
  unset PYTHONPATH
  unset PYTHONHOME
  source ${zumisenv}/bin/activate
  conda-unpack
fi

if grep -q 'zUMIs_directory:' ${yaml} ; then
    sed -i "s|zUMIs_directory:.*|zUMIs_directory: ${zumisdir}|" ${yaml}
else
    echo "zUMIs_directory: ${zumisdir}" >> ${yaml}
fi

${Rexc} ${zumisdir}/checkyaml.R ${yaml} > ${project}.zUMIs_YAMLerror.log
iserror=$(tail ${project}.zUMIs_YAMLerror.log -n1 | awk '{print $2}')

if [[ ${iserror} -eq 1 ]] ; then
    echo "YAML file has an error. Look at the zUMIs_YAMLerror.log or contact developers."
    exit 1
fi

#create main output folder if it didn't exist
if [[ ! -d ${outdir} ]] ; then
  mkdir -p ${outdir}
  if [ $? -ne 0 ] ; then
      echo "Please provide a valid output directory path."
      exit 1
  fi
fi

echo -e "\n\n You provided these parameters:
 YAML file:	${yaml_orig}
 zUMIs directory:		${zumisdir}
 STAR executable		${starexc}
 samtools executable		${samtoolsexc}
 pigz executable		${pigzexc}
 Rscript executable		${Rexc}
 RAM limit:   ${mem_limit}
 zUMIs version ${vers} \n\n" | tee "${outdir}/${project}.zUMIs_runlog.txt"
date

#check for executables
sam_exc_check=$(which ${samtoolsexc})
pigz_exc_check=$(which ${pigzexc})
r_exc_check=$(which ${Rexc})
star_exc_check=$(which ${starexc})

if [[ -z "${sam_exc_check}" ]] ||
   [[ -z "${pigz_exc_check}" ]] ||
   [[ -z "${r_exc_check}" ]] ||
   [[ -z "${star_exc_check}" ]] ; then
    echo "One or more of your executables were not found. Please check back."
    exit 1
fi

# Check if the STAR version used for mapping and the one in the provided STAR index are the same
starver=$(${starexc} --version | sed 's/STAR_//g' | sed 's/\s+//g')
staridxver=$(grep "versionGenome" ${staridxdir}/genomeParameters.txt | awk '{print $2}' | sed 's/\s+//g')

if [[ "${starver}" != "${staridxver}" ]] ; then
  echo "WARNING: The STAR version used for mapping is ${starver} and the STAR index was created using the version ${staridxver}. This may lead to an error while mapping. If you encounter any errors at the mapping stage, please make sure to create the STAR index using STAR ${starver}."
  #exit 1
fi

#create output folders
outdir=$(grep 'out_dir' ${yaml} | awk '{print $2}')
#[ -d ${outdir} ] || mkdir ${outdir}
[ -d ${outdir}/zUMIs_output/ ] || mkdir -p ${outdir}/zUMIs_output/
[ -d ${outdir}/zUMIs_output/expression ] || mkdir -p ${outdir}/zUMIs_output/expression
[ -d ${outdir}/zUMIs_output/stats ] || mkdir -p ${outdir}/zUMIs_output/stats
[ -d ${outdir}/zUMIs_output/.tmpMerge ] || mkdir -p ${outdir}/zUMIs_output/.tmpMerge

# Filtering block
if [[ "${whichStage}" == "Filtering" ]] ; then
  echo "Filtering..."
  date

  f=$(cut -d' ' -f1 <(echo ${fqfiles})) # the first fastq file to determine gzip status
  fullsize=$(stat -L --printf="%s" ${f})

  tmpMerge=${outdir}/zUMIs_output/.tmpMerge/

  if [[ ${f} =~ \.gz$ ]] ; then
      ${pigzexc} -dc ${f} | head -n 4000000 | ${pigzexc} > ${tmpMerge}/${project}.1mio.check.fq.gz
      smallsize=$(stat --printf="%s" ${tmpMerge}/${project}.1mio.check.fq.gz)
      rm ${tmpMerge}/${project}.1mio.check.fq.gz
      nreads=$(expr ${fullsize} \* 1000000 / ${smallsize})

      for i in ${fqfiles} ; do bash ${zumisdir}/splitfq.sh ${i} ${pigzexc} ${num_threads} ${tmpMerge} splitfqgz ${project} ${nreads} & done
      wait
      pref=$(basename ${f} .gz)
      l=$(ls ${tmpMerge}${pref}* | sed "s|${tmpMerge}${pref}||" | sed 's/.gz//')
  else
      head -n 4000000 ${f} > ${tmpMerge}/${project}.1mio.check.fq
      smallsize=$(stat --printf="%s" ${tmpMerge}/${project}.1mio.check.fq)
      rm ${tmpMerge}/${project}.1mio.check.fq
      nreads=$(expr ${fullsize} \* 1000000 / ${smallsize})

      for i in ${fqfiles} ; do bash ${zumisdir}/splitfq.sh ${i} ${pigzexc} ${num_threads} ${tmpMerge} splitfq ${project} ${nreads} & done
      wait
      pref=$(basename ${f})
      l=$(ls ${tmpMerge}${pref}* | sed "s|${tmpMerge}${pref}||")
  fi

  for x in ${l} ; do
    benchmark "FQFilter_${x}" perl ${zumisdir}/fqfilter_v2.pl ${yaml} ${samtoolsexc} ${Rexc} ${pigzexc} ${zumisdir} ${x} &
  done
  wait

  benchmark "MergeBAM" bash ${zumisdir}/mergeBAM.sh ${zumisdir} ${tmpMerge} ${num_threads} ${project} ${outdir} ${yaml} ${samtoolsexc}

  for i in ${fqfiles} ; do
      pref=$(basename ${i} | sed 's/.fastq.gz//' | sed 's/.fq.gz//')
      rm ${tmpMerge}${pref}*gz
  done
  date

  # Barcode detection
  benchmark "BCDetection" ${Rexc} ${zumisdir}/zUMIs-BCdetection.R ${yaml}

  BCbinTable=${outdir}/zUMIs_output/"${project}".BCbinning.txt
  if [[ -f "${BCbinTable}" ]] ; then
      for x in ${l} ; do
        rawbam="${tmpMerge}/${project}.${x}.raw.tagged.bam"
        fixedbam="${tmpMerge}/${project}.${x}.filtered.tagged.bam"
        mv ${fixedbam} ${rawbam}
        benchmark "CorrectBC_${x}" perl ${zumisdir}/correct_BCtag.pl ${rawbam} ${fixedbam} ${BCbinTable} ${samtoolsexc} &
      done
      wait
  fi
  date
fi

# Mapping
if [[ "${whichStage}" == "Filtering" ]] || [[ "${whichStage}" == "Mapping" ]] ; then
  echo "Mapping..."
  date
  benchmark "Mapping" ${Rexc} ${zumisdir}/zUMIs-mapping.R ${yaml}
  date
fi

# Counting
if [[ "${whichStage}" == "Filtering" ]] || [[ "${whichStage}" == "Mapping" ]] || [[ "${whichStage}" == "Counting" ]] ; then
  echo "Counting..."
  date
  benchmark "Counting" ${Rexc} ${zumisdir}/zUMIs-dge2.R ${yaml}
  date
  benchmark "ConvertToLoom" ${Rexc} ${zumisdir}/misc/rds2loom.R ${yaml}
  date
  if [[ "${velo}" == "yes" ]] ; then
    benchmark "Velocyto" ${Rexc} ${zumisdir}/runVelocyto.R ${yaml}
  fi
fi

# Statistics
if [[ "${whichStage}" == "Filtering" ]] || [[ "${whichStage}" == "Mapping" ]] || [[ "${whichStage}" == "Counting" ]] || [[ "${whichStage}" == "Summarising" ]] ; then
  if [[ "${isstats}" == "yes" ]] ; then
    echo "Descriptive statistics..."
    date
    benchmark "Stats" ${Rexc} ${zumisdir}/zUMIs-stats2.R ${yaml}
  fi
  date
fi

# Conda environment close
if [[ ${conda} = true ]] ; then
  source ${zumisenv}/bin/deactivate
fi

date
```

saved as zUMIs\_benchmark.sh

In [ ]:

```

```

and run zUMIs while limiting threads like:

In [ ]:

```
# manually limit threads available, otherwise spawns individual pigz processes with 8 cores each
taskset -c 0-7 /mnt/scratch/leo/zUMIs/zUMIs_benchmark.sh -c -y basic_config.yaml 2> zumis_stderr.txt > zumis_stdout.txt
```

In [ ]:

```

```

### setup umite¶

is run on same machine, same drive, and using same cores as was zUMIs, using following script:

In [ ]:

```
#!/bin/bash

GENOME_DIR="/mnt/scratch/leo/reference/star_mouse_GRCm38_reference_150bp_overhang"
PARALLEL_JOBS=8
FASTQ_DIR="/mnt/scratch/leo/umicount_fastqs"
OUTDIR="/mnt/scratch/leo/umicount_nasalvasc_run/umicount_200cell_output"
LOGDIR="/mnt/scratch/leo/umicount_nasalvasc_run"

date 

mkdir -p $OUTDIR
> "${LOGDIR}/umicount.timing_summary.txt"

# Benchmarking function
benchmark() {
    local label="$1"
    shift
    echo "Running step: $label"
    start_time=$(date +%s)

    /usr/bin/time -v "$@" 2> "${LOGDIR}/umicount.${label}.benchmark.log"

    end_time=$(date +%s)
    duration=$((end_time - start_time))
    echo "Step '$label' took ${duration} seconds" | tee -a "${LOGDIR}/umicount.timing_summary.txt"
}

# List cell IDs
samples=$(ls ${FASTQ_DIR}/*_1.fastq.gz | sed 's/_1.fastq.gz//' | xargs -n1 basename | sort -u)

sorted_samples=$(for f in $samples; do
    size=$(stat -c %s "${FASTQ_DIR}/${f}_1.fastq.gz")
    echo -e "$size\t$f"
done | sort -nr | cut -f2)

# umiextract with flexible TSO
benchmark "umiextract" umiextract \
	-1 $(echo "$sorted_samples" | xargs -I{} echo -n "${FASTQ_DIR}/{}_1.fastq.gz ") \
	-2 $(echo "$sorted_samples" | xargs -I{} echo -n "${FASTQ_DIR}/{}_2.fastq.gz ") \
	-d "$OUTDIR" \
	-c "$PARALLEL_JOBS" \
	--umilen 8 \
	--anchor "ATTGCGCAATG" \
	--trailing "GGG" \
	--search_region 30 \
	--fuzzy_umi 

# STAR alignments
run_star() {
    sample="$1"
    r1="${OUTDIR}/${sample}_1_umiextract.fastq.gz"
    r2="${OUTDIR}/${sample}_2_umiextract.fastq.gz"
    outprefix="${OUTDIR}/${sample}_"

    /mnt/scratch/tools/STAR-2.7.3a/bin/Linux_x86_64/STAR \
	--genomeDir "$GENOME_DIR" \
        --readFilesIn "$r1" "$r2" \
        --readFilesCommand zcat \
        --outFileNamePrefix "$outprefix" \
        --outSAMtype BAM Unsorted \
        --clip3pAdapterSeq CTGTCTCTTATACACATCT \
        --runThreadN 1 \
        --genomeLoad LoadAndKeep
}

export -f run_star
export sorted_samples
export GENOME_DIR FASTQ_DIR OUTDIR PARALLEL_JOBS

benchmark "load_STAR_genome" /mnt/scratch/tools/STAR-2.7.3a/bin/Linux_x86_64/STAR \
	--genomeDir "$GENOME_DIR" \
	--genomeLoad LoadAndExit \
	--runThreadN "$PARALLEL_JOBS"

benchmark "STAR_alignments" bash -c 'echo "$sorted_samples" | parallel -j "$PARALLEL_JOBS" run_star {}'

/mnt/scratch/tools/STAR-2.7.3a/bin/Linux_x86_64/STAR \
    --genomeDir "$GENOME_DIR" \
    --genomeLoad Remove

# Sort BAMs by readname
benchmark "sort_bams" bash -c 'echo "$1" | parallel -j "$2" "$3/samtools sort -n $4/{}_Aligned.out.bam > $4/{}_Aligned.sort.bam && rm $4/{}_Aligned.out.bam"' _ "$sorted_samples" "$PARALLEL_JOBS" "/mnt/scratch/tools/samtools-1.21" "$OUTDIR"

# umicount 
benchmark "GTF_parse_and_dump" umicount \
	-g /mnt/scratch/leo/reference/Mus_musculus.GRCm38.96.gtf \
	--GTF_dump "${OUTDIR}/umicount_GTF_dump.pkl"

benchmark "umicount" umicount \
	--bams $(echo "$sorted_samples" | xargs -I{} echo -n "${OUTDIR}/{}_Aligned.sort.bam ") \
	-d "$OUTDIR" \
	-c "$PARALLEL_JOBS" \
	--GTF_skip_parse "${OUTDIR}/umicount_GTF_dump.pkl" \
    --mm_count_primary \
	--UMI_correct

echo "Finished"

date
```

In [ ]:

```

```

a second script was used to test runs without fuzzy umi detection:

In [ ]:

```
#!/bin/bash

GENOME_DIR="/mnt/scratch/leo/reference/star_mouse_GRCm38_reference_150bp_overhang"
PARALLEL_JOBS=8
FASTQ_DIR="/mnt/scratch/leo/umicount_fastqs"
OUTDIR="/mnt/scratch/leo/umicount_run/umicount_200cell_output_nofuzzy"
LOGDIR="/mnt/scratch/leo/umicount_run"

date 

mkdir -p $OUTDIR
> "${LOGDIR}/umicount_nofuzzy.timing_summary.txt"

# Benchmarking function
benchmark() {
    local label="$1"
    shift
    echo "Running step: $label"
    start_time=$(date +%s)

    /usr/bin/time -v "$@" 2> "${LOGDIR}/umicount_nofuzzy.${label}.benchmark.log"

    end_time=$(date +%s)
    duration=$((end_time - start_time))
    echo "Step '$label' took ${duration} seconds" | tee -a "${LOGDIR}/umicount_nofuzzy.timing_summary.txt"
}

# List cell IDs
samples=$(ls ${FASTQ_DIR}/*_R1.fastq.gz | sed 's/_R1.fastq.gz//' | xargs -n1 basename | sort -u)

sorted_samples=$(for f in $samples; do
    size=$(stat -c %s "${FASTQ_DIR}/${f}_R1.fastq.gz")
    echo -e "$size\t$f"
done | sort -nr | cut -f2)

# umiextract with flexible TSO
benchmark "umiextract" umiextract \
	-1 $(echo "$sorted_samples" | xargs -I{} echo -n "${FASTQ_DIR}/{}_R1.fastq.gz ") \
	-2 $(echo "$sorted_samples" | xargs -I{} echo -n "${FASTQ_DIR}/{}_R2.fastq.gz ") \
	-d "$OUTDIR" \
	-c "$PARALLEL_JOBS" \
	--umilen 8 \
	--anchor "ATTGCGCAATG" \
	--trailing "GGG"

date
```

In [ ]:

```

```

### additional reproducibility information¶

this section aims to document the environment in which benchmarks were carried out, including available software and hardware

as noted above, available cpus were manually limited using the unix program taskset, which coincidentally limited benchmark runs to precisely the same cpus. The benchmarks themselves were run on a dedicated virtual machine supplied via DKFZ OpenStack infrastructure. This machine had 64gb of available RAM and 28 cores, of which 8 were used for benchmarking. Each core is identical and has the following associated data:

In [ ]:

```
> cat /proc/cpuinfo
processor       : 0
vendor_id       : GenuineIntel
cpu family      : 6
model           : 61
model name      : Intel Core Processor (Broadwell, IBRS)
stepping        : 2
microcode       : 0x1
cpu MHz         : 1999.998
cache size      : 16384 KB
physical id     : 0
siblings        : 1
core id         : 0
cpu cores       : 1
apicid          : 0
initial apicid  : 0
fpu             : yes
fpu_exception   : yes
cpuid level     : 13
wp              : yes
flags           : fpu vme de pse tsc msr pae mce cx8 apic sep mtrr pge mca cmov pat pse36 clflush mmx fxsr sse sse2 ss syscall nx pdpe1gb rdtscp lm constant_tsc rep_good nopl xtopology cpuid tsc_known_freq pni pclmulqdq vmx ssse3 fma cx16 pcid sse4_1 sse4_2 x2apic movbe popcnt tsc_deadline_timer aes xsave avx f16c rdrand hypervisor lahf_lm abm 3dnowprefetch cpuid_fault invpcid_single pti ssbd ibrs ibpb stibp tpr_shadow vnmi flexpriority ept vpid ept_ad fsgsbase tsc_adjust bmi1 hle avx2 smep bmi2 erms invpcid rtm rdseed adx smap xsaveopt arat umip arch_capabilities
vmx flags       : vnmi preemption_timer posted_intr invvpid ept_x_only ept_ad ept_1gb flexpriority apicv tsc_offset vtpr mtf vapic ept vpid unrestricted_guest vapic_reg vid shadow_vmcs pml
bugs            : cpu_meltdown spectre_v1 spectre_v2 spec_store_bypass l1tf mds swapgs taa srbds mmio_unknown
bogomips        : 3999.99
clflush size    : 64
cache_alignment : 64
address sizes   : 46 bits physical, 48 bits virtual
power management:
```

In [ ]:

```

```

as zUMIs supplies its own conda environment with pre-installed executables for pigz, samtools, etc, benchmarks were run while making use of this environment, with the sole exception of supplying my own STAR v2.7.3a executable as this is the one also used in umite benchmarks. Otherwise the supplied conda environment was used in zUMIs runs (see https://github.com/sdparekh/zUMIs).

for umite, a benchmarking environment was created which included:

- python 3.10.14
- pip v24.0
- and via pip:
- HTSeq==2.0.9
- regex==2024.11.6
- RapidFuzz==3.13.0

In [ ]:

```

```

## measure UMI gain and select benchmark cell-sets¶

first we check UMI presence and gain by fuzzy matching among all cells from the nasal dataset, then create 200/400/800 cell-sets for benchmarking

In [ ]:

```

```

### analzye UMI gain¶

using the above script to derive both fuzzy and strict UMI counts, then to gauge how much is gained. the output file parsed below simply captures the stdout of umiextract, which prints the number of internal and umi reads as well as the total read count per cell

In [62]:

```
nofuz_stats = pd.DataFrame(columns=['cell', 'reads', 'umis', 'written'])
```

In [63]:

```
with open('/mnt/volume/resources/external_SS3_cells/2nd_nasal_dataset_complete_bench/umicount_umiextract.nofuz.output') as f:
    for n, i in enumerate(f.readlines()):
        if i:
            nofuz_stats.loc[n] = extract_umi_stats(i)
```

In [64]:

```
nofuz_stats = nofuz_stats.set_index('cell').drop(index=[None])
```

In [66]:

```
nofuz_stats.index = [i.split('_')[0] for i in nofuz_stats.index]
```

In [67]:

```
nofuz_stats = nofuz_stats.astype('int64')
```

In [68]:

```
nofuz_stats
```

Out[68]:

|  | reads | umis | written |
| --- | --- | --- | --- |
| SRR19885507 | 318840 | 174332 | 318840 |
| SRR19885301 | 322344 | 109011 | 322344 |
| SRR19885158 | 302677 | 172220 | 302677 |
| SRR19885500 | 326264 | 93604 | 326264 |
| SRR19885927 | 318745 | 148780 | 318745 |
| ... | ... | ... | ... |
| SRR19885018 | 1146951 | 752925 | 1146951 |
| SRR19885289 | 1066425 | 552520 | 1066425 |
| SRR19885129 | 1126202 | 552130 | 1126202 |
| SRR19885286 | 1090300 | 673671 | 1090300 |
| SRR19885806 | 1078357 | 688763 | 1078357 |

1423 rows × 3 columns

In [ ]:

```

```

In [69]:

```
yesfuz_stats = pd.DataFrame(columns=['cell', 'reads', 'umis', 'written'])
```

In [70]:

```
with open('/mnt/volume/resources/external_SS3_cells/2nd_nasal_dataset_complete_bench/umicount_umiextract.yesfuz.output') as f:
    for n, i in enumerate(f.readlines()):
        if i:
            yesfuz_stats.loc[n] = extract_umi_stats(i)
```

In [71]:

```
yesfuz_stats = yesfuz_stats.set_index('cell').drop(index=[None])
```

In [72]:

```
yesfuz_stats.index = [i.split('_')[0] for i in yesfuz_stats.index]
```

In [73]:

```
yesfuz_stats = yesfuz_stats.astype('int64')
```

In [74]:

```
yesfuz_stats
```

Out[74]:

|  | reads | umis | written |
| --- | --- | --- | --- |
| SRR19886032 | 276498 | 135813 | 276498 |
| SRR19885578 | 291086 | 114578 | 291086 |
| SRR19885259 | 293474 | 172989 | 293474 |
| SRR19886087 | 286659 | 154886 | 286659 |
| SRR19886281 | 295610 | 164063 | 295610 |
| ... | ... | ... | ... |
| SRR19885146 | 1331512 | 710895 | 1331512 |
| SRR19885216 | 1307853 | 799305 | 1307853 |
| SRR19885150 | 1318194 | 784403 | 1318194 |
| SRR19885522 | 1295670 | 745206 | 1295670 |
| SRR19885297 | 1255936 | 655213 | 1255936 |

1440 rows × 3 columns

In [ ]:

```

```

In [82]:

```
yesfuz_stats = yesfuz_stats.reindex(nofuz_stats.index).dropna()
```

In [83]:

```
assert ((yesfuz_stats['reads'] - nofuz_stats['reads']) == 0).all()
```

In [90]:

```
assert ((yesfuz_stats['written'] - nofuz_stats['written']) <= 1).all()
```

In [ ]:

```

```

now we compare between the two conditions fuzzy and strict UMI matching

In [91]:

```
umiediff = pd.DataFrame()
```

In [92]:

```
umiediff['reads'] = yesfuz_stats['reads']
umiediff['fuzz'] = yesfuz_stats['umis']
umiediff['nofuzz'] = nofuz_stats['umis']
```

In [93]:

```
umiediff['umigain'] = umiediff['fuzz'] - umiediff['nofuzz']
umiediff['umigain_perc'] = umiediff['umigain'] / umiediff['nofuzz']
```

In [94]:

```
umiediff['umiperc'] = umiediff['nofuzz'] / umiediff['reads']
```

In [130]:

```
fmqcdata = pd.read_csv('XX_output/nasal_2nd_dataset_multiqc_pass_cells.csv') # the multiqc output from above
```

In [137]:

```
umiediff['mqcpass'] = umiediff.index.isin(fmqcdata['Sample'].unique())
```

In [138]:

```
umiediff
```

Out[138]:

|  | reads | fuzz | nofuzz | umigain | umigain\_perc | umiperc | mqcpass |
| --- | --- | --- | --- | --- | --- | --- | --- |
| SRR19885507 | 318840 | 183696 | 174332 | 9364 | 0.054 | 0.547 | True |
| SRR19885301 | 322344 | 115644 | 109011 | 6633 | 0.061 | 0.338 | True |
| SRR19885158 | 302677 | 181537 | 172220 | 9317 | 0.054 | 0.569 | True |
| SRR19885500 | 326264 | 100494 | 93604 | 6890 | 0.074 | 0.287 | True |
| SRR19885927 | 318745 | 158181 | 148780 | 9401 | 0.063 | 0.467 | True |
| ... | ... | ... | ... | ... | ... | ... | ... |
| SRR19885018 | 1146951 | 796550 | 752925 | 43625 | 0.058 | 0.656 | False |
| SRR19885289 | 1066425 | 579159 | 552520 | 26639 | 0.048 | 0.518 | False |
| SRR19885129 | 1126202 | 582017 | 552130 | 29887 | 0.054 | 0.490 | False |
| SRR19885286 | 1090300 | 703888 | 673671 | 30217 | 0.045 | 0.618 | False |
| SRR19885806 | 1078357 | 723999 | 688763 | 35236 | 0.051 | 0.639 | False |

1423 rows × 7 columns

In [ ]:

```

```

select a subset of cells based on read parameters

In [185]:

```
minreads = 5e4
maxreads = 5e5
mingain = 0.05
maxgain = 0.11
```

In [186]:

```
cellpick = umiediff[(umiediff['reads'] > minreads) & 
                    (umiediff['reads'] < maxreads) &
                    (umiediff['umigain_perc'] > mingain) & 
                    (umiediff['umigain_perc'] < maxgain)]
```

In [187]:

```
cellpick
```

Out[187]:

|  | reads | fuzz | nofuzz | umigain | umigain\_perc | umiperc | mqcpass |
| --- | --- | --- | --- | --- | --- | --- | --- |
| SRR19885507 | 318840 | 183696 | 174332 | 9364 | 0.054 | 0.547 | True |
| SRR19885301 | 322344 | 115644 | 109011 | 6633 | 0.061 | 0.338 | True |
| SRR19885158 | 302677 | 181537 | 172220 | 9317 | 0.054 | 0.569 | True |
| SRR19885500 | 326264 | 100494 | 93604 | 6890 | 0.074 | 0.287 | True |
| SRR19885927 | 318745 | 158181 | 148780 | 9401 | 0.063 | 0.467 | True |
| ... | ... | ... | ... | ... | ... | ... | ... |
| SRR19885295 | 53038 | 21013 | 19448 | 1565 | 0.080 | 0.367 | False |
| SRR19885618 | 52424 | 19974 | 18418 | 1556 | 0.084 | 0.351 | False |
| SRR19884929 | 50087 | 14030 | 12696 | 1334 | 0.105 | 0.253 | False |
| SRR19885774 | 50277 | 18172 | 16379 | 1793 | 0.109 | 0.326 | False |
| SRR19885181 | 50501 | 20297 | 18615 | 1682 | 0.090 | 0.369 | False |

850 rows × 7 columns

In [ ]:

```

```

In [625]:

```
f = plt.figure(figsize=(6, 3.5))
ax = f.gca()

p = umiediff[umiediff['reads'] >= 20000].sort_values('umiperc')

sc = plt.scatter(p['umigain_perc'], p['reads'], c=p['umiperc'], 
                 ec='k', lw=0.5, s=60,
                 rasterized=True)


from matplotlib.patches import Rectangle

ax.add_patch(Rectangle([mingain, minreads], 
                       width=maxgain - mingain, 
                       height=maxreads - minreads,
                       fill=None, ec='r', lw=3, ls='--'))

plt.ylim(18e3, 4e6)
plt.xlim(0.03, 0.23)
plt.yscale('symlog')

plt.xlabel('%UMIs gained by fuzzy matching')
plt.ylabel('Cell library size (#Reads)')

sns.despine(ax=ax)

from mpl_toolkits.axes_grid1.inset_locator import inset_axes
axins = inset_axes(ax, width='3%', height='30%', borderpad=0, loc=3,
                   bbox_to_anchor=(0.89, 0.69, 1, 1), bbox_transform=ax.transAxes)

cb = f.colorbar(sc, cax=axins)
cb.set_label('%UMIs', fontsize=10, rotation=90, labelpad=-10)
cb.set_ticks([p['umiperc'].max(), p['umiperc'].min()])
cb.set_ticklabels([int(round(p['umiperc'].max()*100, 1)), int(round(p['umiperc'].min()*100, 1))])

plt.tight_layout()
plt.savefig('XX_output/figures/benchmark_v2_scatter_with_cells_noline.svg', format='svg')
plt.show()
```

In [ ]:

```

```

### select cell-sets (and run pipelines)¶

according to the read parameters outlined above, no replacement

In [ ]:

```

```

200-cells

In [198]:

```
cellpick_selected = cellpick.sample(200)
```

In [199]:

```
cellpick_selected['mqcpass'].value_counts()
```

Out[199]:

```
mqcpass
True     133
False     67
Name: count, dtype: int64
```

In [236]:

```
cellpick_selected.to_csv('XX_output/nasal_2nd_dataset_benchmark_cellset.csv')
```

In [ ]:

```

```

400-cells (previous 200 + 200 more)

In [264]:

```
cellpick_selected2 = cellpick.drop(index=cellpick_selected.index).sample(200)
```

In [265]:

```
cellpick_selected2['mqcpass'].value_counts()
```

Out[265]:

```
mqcpass
True     134
False     66
Name: count, dtype: int64
```

In [ ]:

```

```

In [269]:

```
pd.concat([cellpick_selected, 
           cellpick_selected2]).to_csv('XX_output/nasal_2nd_dataset_benchmark_cellset.400.csv')
```

In [ ]:

```

```

800-cells (previous 400 + 400 more)

In [278]:

```
cellpick_selected3 = cellpick.drop(index=cellpick_selected.index).drop(cellpick_selected2.index).sample(400)
```

In [279]:

```
cellpick_selected3['mqcpass'].value_counts()
```

Out[279]:

```
mqcpass
True     262
False    138
Name: count, dtype: int64
```

In [ ]:

```

```

In [280]:

```
pd.concat([cellpick_selected, 
           cellpick_selected2,
           cellpick_selected3]).to_csv('XX_output/nasal_2nd_dataset_benchmark_cellset.800.csv')
```

In [ ]:

```

```

upon selecting these cell sets, benchmarks for each cell set (200/400/800) were run for umite and zUMIs each, using the above described functions and scripts.

In [ ]:

```

```

### plot fuzzy matching time gain¶

times taken from above mentioned pipeline runs with/without fuzzy matching

In [66]:

```
time_nofuz = 3356
time_yesfuz = 4718
```

In [672]:

```
cells = 800
```

In [697]:

```
f = plt.figure(figsize=(5, 1.5))
grid = f.add_gridspec(ncols=2, nrows=1, 
         width_ratios=[2, 1], wspace=0.3)

ax1 = f.add_subplot(grid[0,0])
ax2 = f.add_subplot(grid[0,1])

p1 = umiediff[umiediff['mqcpass'] == True]['umigain_perc']
p2 = umiediff[umiediff['mqcpass'] == False]['umigain_perc'].clip(0, 0.2)

ax1.boxplot([p1, p2], widths=0.35, vert=False, showfliers=False)

ax1.text(0.01, 2, 'n=%s' %len(p2))
ax1.text(0.01, 1, 'n=%s' %len(p1))

ax1.text(np.mean(p1), 1, '%.2f' %np.mean(p1))
ax1.text(np.mean(p2), 2, '%.2f' %np.mean(p2))

ax1.set_xlim(0, 0.175)

ax1.set_yticklabels(['Pass', 'Fail'])
ax1.set_ylabel('FastQC\nSeq. quality')
ax1.set_xlabel('%UMIs gained by fuzzy matching')

###################

ax2.bar(x=[1, 2], height=[time_nofuz/cells, time_yesfuz/cells], 
        width=0.66, ec='k', lw=0.5, color=['indianred', 'firebrick'])

ax2.text(1.1, 5, '%.2fs' %((time_yesfuz/cells) - (time_nofuz/cells)))

ax2.set_xlim(0.5, 2.5)
ax2.set_ylabel('Time per cell (s)')
ax2.set_xticklabels(['', 'No', 'Yes'])
ax2.set_xlabel('Fuzzy UMI\nmatching')

for ax in [ax1, ax2]:
    sns.despine(ax=ax)

plt.tight_layout()
#plt.savefig('XX_output/figures/benchmark_v2_umiextract_fuzstats.svg', format='svg')
plt.show()
```

In [ ]:

```

```

## Results: analyze benchmarks and create plots¶

the above pipeline scripts write the output of /usr/bin/time -v to individual logfiles, which we now read and analyze

In [ ]:

```

```

### helper functions¶

In [9]:

```
from datetime import datetime
```

In [10]:

```
fmt = '%M:%S'
```

In [11]:

```
def walltime_to_seconds(walltime):
    minutes, seconds = map(int, walltime.split(":"))
    return minutes * 60 + seconds + 1
```

In [ ]:

```

```

In [12]:

```
import re
```

In [13]:

```
def parse_elapsed_times(log_text):
    time_dict = {}
    # Pattern to capture filename and time
    pattern = re.compile(r'^(.*?):\s+Elapsed.*?: ([0-9]+:)?([0-9]+):([0-9.]+)', re.MULTILINE)
    
    for match in pattern.finditer(log_text):
        file = match.group(1)
        h = match.group(2)
        m = int(match.group(3))
        s = float(match.group(4))
        total_seconds = (int(h[:-1]) * 3600 if h else 0) + m * 60 + s
        time_dict[file] = total_seconds
    
    return time_dict
```

In [14]:

```
def parse_max_memory_usage(log_text):
    mem_dict = {}
    # Pattern to capture filename and memory size
    pattern = re.compile(r'^(.*?):\s+Maximum resident set size \(kbytes\): (\d+)', re.MULTILINE)
    
    for match in pattern.finditer(log_text):
        file = match.group(1)
        mem_kb = int(match.group(2))
        mem_dict[file] = mem_kb
    
    return mem_dict
```

In [ ]:

```

```

### zUMIs pipeline timing¶

the modified zUMIs\_benchmark.sh script produces a log file capturing gnu time output for each call to the benchmark function, i.e. each central pipeline step. These files are saved to a log directory and contain the name of the step (e.g. "Counting"). As gnu time reports several runtime statistics, we extract the relevant time (Elapsed time) and memory (Maximum resident set size) fields from zUMIs log files via grep, and the consumed resources analyzed using regexes within the above-defined helper functions. The mem\_total field denotes the size of the entire dataset + pipeline outputs, as measured using the unix program du

In [ ]:

```

```

#### 200 cell¶

In [15]:

```
z200_time_total_s = 4978 # 16:31:39 - 17:54:37
```

In [16]:

```
z200_mem_total = 16317000 # disk space used (measured upon completion)
```

In [ ]:

```

```

In [17]:

```
z200_walltime = parse_elapsed_times(
"""
zumis_200cell_logs/zUMIs.BCDetection.benchmark.log:	Elapsed (wall clock) time (h:mm:ss or m:ss): 0:00.80
zumis_200cell_logs/zUMIs.ConvertToLoom.benchmark.log:	Elapsed (wall clock) time (h:mm:ss or m:ss): 0:15.31
zumis_200cell_logs/zUMIs.Counting.benchmark.log:	Elapsed (wall clock) time (h:mm:ss or m:ss): 21:48.29
zumis_200cell_logs/zUMIs.FQFilter_zumis_nasalvasc_200cellaa.benchmark.log:	Elapsed (wall clock) time (h:mm:ss or m:ss): 8:01.02
zumis_200cell_logs/zUMIs.FQFilter_zumis_nasalvasc_200cellab.benchmark.log:	Elapsed (wall clock) time (h:mm:ss or m:ss): 7:59.57
zumis_200cell_logs/zUMIs.FQFilter_zumis_nasalvasc_200cellac.benchmark.log:	Elapsed (wall clock) time (h:mm:ss or m:ss): 8:06.23
zumis_200cell_logs/zUMIs.FQFilter_zumis_nasalvasc_200cellad.benchmark.log:	Elapsed (wall clock) time (h:mm:ss or m:ss): 7:56.93
zumis_200cell_logs/zUMIs.FQFilter_zumis_nasalvasc_200cellae.benchmark.log:	Elapsed (wall clock) time (h:mm:ss or m:ss): 8:05.64
zumis_200cell_logs/zUMIs.FQFilter_zumis_nasalvasc_200cellaf.benchmark.log:	Elapsed (wall clock) time (h:mm:ss or m:ss): 7:56.74
zumis_200cell_logs/zUMIs.FQFilter_zumis_nasalvasc_200cellag.benchmark.log:	Elapsed (wall clock) time (h:mm:ss or m:ss): 7:57.90
zumis_200cell_logs/zUMIs.FQFilter_zumis_nasalvasc_200cellah.benchmark.log:	Elapsed (wall clock) time (h:mm:ss or m:ss): 6:05.08
zumis_200cell_logs/zUMIs.Mapping.benchmark.log:	Elapsed (wall clock) time (h:mm:ss or m:ss): 43:11.09
zumis_200cell_logs/zUMIs.MergeBAM.benchmark.log:	Elapsed (wall clock) time (h:mm:ss or m:ss): 0:00.01
zumis_200cell_logs/zUMIs.Stats.benchmark.log:	Elapsed (wall clock) time (h:mm:ss or m:ss): 3:19.89
"""
)
```

In [18]:

```
z200_memory = parse_max_memory_usage(
"""
zumis_200cell_logs/zUMIs.BCDetection.benchmark.log:	Maximum resident set size (kbytes): 97776
zumis_200cell_logs/zUMIs.ConvertToLoom.benchmark.log:	Maximum resident set size (kbytes): 466576
zumis_200cell_logs/zUMIs.Counting.benchmark.log:	Maximum resident set size (kbytes): 17821864
zumis_200cell_logs/zUMIs.FQFilter_zumis_nasalvasc_200cellaa.benchmark.log:	Maximum resident set size (kbytes): 61164
zumis_200cell_logs/zUMIs.FQFilter_zumis_nasalvasc_200cellab.benchmark.log:	Maximum resident set size (kbytes): 61012
zumis_200cell_logs/zUMIs.FQFilter_zumis_nasalvasc_200cellac.benchmark.log:	Maximum resident set size (kbytes): 61028
zumis_200cell_logs/zUMIs.FQFilter_zumis_nasalvasc_200cellad.benchmark.log:	Maximum resident set size (kbytes): 60944
zumis_200cell_logs/zUMIs.FQFilter_zumis_nasalvasc_200cellae.benchmark.log:	Maximum resident set size (kbytes): 61140
zumis_200cell_logs/zUMIs.FQFilter_zumis_nasalvasc_200cellaf.benchmark.log:	Maximum resident set size (kbytes): 60932
zumis_200cell_logs/zUMIs.FQFilter_zumis_nasalvasc_200cellag.benchmark.log:	Maximum resident set size (kbytes): 61140
zumis_200cell_logs/zUMIs.FQFilter_zumis_nasalvasc_200cellah.benchmark.log:	Maximum resident set size (kbytes): 60976
zumis_200cell_logs/zUMIs.Mapping.benchmark.log:	Maximum resident set size (kbytes): 22200304
zumis_200cell_logs/zUMIs.MergeBAM.benchmark.log:	Maximum resident set size (kbytes): 3524
zumis_200cell_logs/zUMIs.Stats.benchmark.log:	Maximum resident set size (kbytes): 2512040
"""
)
```

In [ ]:

```

```

In [19]:

```
z200_times = {i.split('.')[1]:{'walltime':z200_walltime[i], 
                           'maxmem':z200_memory[i]} for i in z200_memory.keys()}
```

In [20]:

```
z200_times['remultiplex'] = {'walltime':walltime_to_seconds('3:45'), 'maxmem':97776}
```

In [21]:

```
fqsteps = [i for i in z200_times.keys() if i.startswith('FQFilter')]

z200_times['fqfilter'] = {
    'walltime':max([z200_times[i]['walltime'] for i in fqsteps]),
    'maxmem':sum([z200_times[i]['maxmem'] for i in fqsteps])
}

for i in fqsteps:
    del z200_times[i]
```

In [22]:

```
z200_times
```

Out[22]:

```
{'BCDetection': {'walltime': 0.8, 'maxmem': 97776},
 'ConvertToLoom': {'walltime': 15.31, 'maxmem': 466576},
 'Counting': {'walltime': 1308.29, 'maxmem': 17821864},
 'Mapping': {'walltime': 2591.09, 'maxmem': 22200304},
 'MergeBAM': {'walltime': 0.01, 'maxmem': 3524},
 'Stats': {'walltime': 199.89, 'maxmem': 2512040},
 'remultiplex': {'walltime': 226, 'maxmem': 97776},
 'fqfilter': {'walltime': 486.23, 'maxmem': 488336}}
```

In [ ]:

```

```

#### 400 cell¶

In [23]:

```
z400_time_total_s = 9826 # 12:04:21 - 14:48:07
```

In [24]:

```
z400_mem_total = 32128444
```

In [ ]:

```

```

In [25]:

```
z400_walltime = parse_elapsed_times(
"""
zumis_400cell_logs/zUMIs.BCDetection.benchmark.log:	Elapsed (wall clock) time (h:mm:ss or m:ss): 0:00.78
zumis_400cell_logs/zUMIs.ConvertToLoom.benchmark.log:	Elapsed (wall clock) time (h:mm:ss or m:ss): 0:22.04
zumis_400cell_logs/zUMIs.Counting.benchmark.log:	Elapsed (wall clock) time (h:mm:ss or m:ss): 39:55.31
zumis_400cell_logs/zUMIs.FQFilter_zumis_nasalvasc_400cellaa.benchmark.log:	Elapsed (wall clock) time (h:mm:ss or m:ss): 16:12.82
zumis_400cell_logs/zUMIs.FQFilter_zumis_nasalvasc_400cellab.benchmark.log:	Elapsed (wall clock) time (h:mm:ss or m:ss): 16:03.50
zumis_400cell_logs/zUMIs.FQFilter_zumis_nasalvasc_400cellac.benchmark.log:	Elapsed (wall clock) time (h:mm:ss or m:ss): 15:46.52
zumis_400cell_logs/zUMIs.FQFilter_zumis_nasalvasc_400cellad.benchmark.log:	Elapsed (wall clock) time (h:mm:ss or m:ss): 15:53.72
zumis_400cell_logs/zUMIs.FQFilter_zumis_nasalvasc_400cellae.benchmark.log:	Elapsed (wall clock) time (h:mm:ss or m:ss): 15:46.74
zumis_400cell_logs/zUMIs.FQFilter_zumis_nasalvasc_400cellaf.benchmark.log:	Elapsed (wall clock) time (h:mm:ss or m:ss): 15:39.89
zumis_400cell_logs/zUMIs.FQFilter_zumis_nasalvasc_400cellag.benchmark.log:	Elapsed (wall clock) time (h:mm:ss or m:ss): 16:01.68
zumis_400cell_logs/zUMIs.FQFilter_zumis_nasalvasc_400cellah.benchmark.log:	Elapsed (wall clock) time (h:mm:ss or m:ss): 14:15.72
zumis_400cell_logs/zUMIs.Mapping.benchmark.log:	Elapsed (wall clock) time (h:mm:ss or m:ss): 1:27:44
zumis_400cell_logs/zUMIs.MergeBAM.benchmark.log:	Elapsed (wall clock) time (h:mm:ss or m:ss): 0:00.01
zumis_400cell_logs/zUMIs.Stats.benchmark.log:	Elapsed (wall clock) time (h:mm:ss or m:ss): 6:46.41
"""
)
```

In [26]:

```
z400_memory = parse_max_memory_usage(
"""
zumis_400cell_logs/zUMIs.BCDetection.benchmark.log:	Maximum resident set size (kbytes): 98024
zumis_400cell_logs/zUMIs.ConvertToLoom.benchmark.log:	Maximum resident set size (kbytes): 638344
zumis_400cell_logs/zUMIs.Counting.benchmark.log:	Maximum resident set size (kbytes): 35604152
zumis_400cell_logs/zUMIs.FQFilter_zumis_nasalvasc_400cellaa.benchmark.log:	Maximum resident set size (kbytes): 60904
zumis_400cell_logs/zUMIs.FQFilter_zumis_nasalvasc_400cellab.benchmark.log:	Maximum resident set size (kbytes): 61092
zumis_400cell_logs/zUMIs.FQFilter_zumis_nasalvasc_400cellac.benchmark.log:	Maximum resident set size (kbytes): 60644
zumis_400cell_logs/zUMIs.FQFilter_zumis_nasalvasc_400cellad.benchmark.log:	Maximum resident set size (kbytes): 60828
zumis_400cell_logs/zUMIs.FQFilter_zumis_nasalvasc_400cellae.benchmark.log:	Maximum resident set size (kbytes): 60888
zumis_400cell_logs/zUMIs.FQFilter_zumis_nasalvasc_400cellaf.benchmark.log:	Maximum resident set size (kbytes): 60728
zumis_400cell_logs/zUMIs.FQFilter_zumis_nasalvasc_400cellag.benchmark.log:	Maximum resident set size (kbytes): 61044
zumis_400cell_logs/zUMIs.FQFilter_zumis_nasalvasc_400cellah.benchmark.log:	Maximum resident set size (kbytes): 61172
zumis_400cell_logs/zUMIs.Mapping.benchmark.log:	Maximum resident set size (kbytes): 22201576
zumis_400cell_logs/zUMIs.MergeBAM.benchmark.log:	Maximum resident set size (kbytes): 3472
zumis_400cell_logs/zUMIs.Stats.benchmark.log:	Maximum resident set size (kbytes): 4455340
"""
)
```

In [ ]:

```

```

In [27]:

```
z400_times = {i.split('.')[1]:{'walltime':z400_walltime[i], 
                           'maxmem':z400_memory[i]} for i in z400_memory.keys()}
```

In [28]:

```
z400_times['remultiplex'] = {'walltime':walltime_to_seconds('2:42'), 'maxmem':86352}
```

In [29]:

```
fqsteps = [i for i in z400_times.keys() if i.startswith('FQFilter')]

z400_times['fqfilter'] = {
    'walltime':max([z400_times[i]['walltime'] for i in fqsteps]),
    'maxmem':sum([z400_times[i]['maxmem'] for i in fqsteps])
}

for i in fqsteps:
    del z400_times[i]
```

In [30]:

```
z400_times
```

Out[30]:

```
{'BCDetection': {'walltime': 0.78, 'maxmem': 98024},
 'ConvertToLoom': {'walltime': 22.04, 'maxmem': 638344},
 'Counting': {'walltime': 2395.31, 'maxmem': 35604152},
 'Mapping': {'walltime': 5264.0, 'maxmem': 22201576},
 'MergeBAM': {'walltime': 0.01, 'maxmem': 3472},
 'Stats': {'walltime': 406.40999999999997, 'maxmem': 4455340},
 'remultiplex': {'walltime': 163, 'maxmem': 86352},
 'fqfilter': {'walltime': 972.82, 'maxmem': 487300}}
```

In [ ]:

```

```

#### 800 cell¶

In [31]:

```
z800_time_total_s = 19009 # 09:41:38 - 14:58:27
```

In [32]:

```
z800_mem_total = 75753916
```

In [ ]:

```

```

In [33]:

```
z800_walltime = parse_elapsed_times(
"""
zumis_800cell_logs/zUMIs.BCDetection.benchmark.log:	Elapsed (wall clock) time (h:mm:ss or m:ss): 0:00.91
zumis_800cell_logs/zUMIs.ConvertToLoom.benchmark.log:	Elapsed (wall clock) time (h:mm:ss or m:ss): 0:42.45
zumis_800cell_logs/zUMIs.Counting.benchmark.log:	Elapsed (wall clock) time (h:mm:ss or m:ss): 1:31:46
zumis_800cell_logs/zUMIs.FQFilter_zumis_nasalvasc_800cellaa.benchmark.log:	Elapsed (wall clock) time (h:mm:ss or m:ss): 30:06.41
zumis_800cell_logs/zUMIs.FQFilter_zumis_nasalvasc_800cellab.benchmark.log:	Elapsed (wall clock) time (h:mm:ss or m:ss): 30:18.31
zumis_800cell_logs/zUMIs.FQFilter_zumis_nasalvasc_800cellac.benchmark.log:	Elapsed (wall clock) time (h:mm:ss or m:ss): 30:05.45
zumis_800cell_logs/zUMIs.FQFilter_zumis_nasalvasc_800cellad.benchmark.log:	Elapsed (wall clock) time (h:mm:ss or m:ss): 29:49.01
zumis_800cell_logs/zUMIs.FQFilter_zumis_nasalvasc_800cellae.benchmark.log:	Elapsed (wall clock) time (h:mm:ss or m:ss): 30:20.98
zumis_800cell_logs/zUMIs.FQFilter_zumis_nasalvasc_800cellaf.benchmark.log:	Elapsed (wall clock) time (h:mm:ss or m:ss): 29:53.96
zumis_800cell_logs/zUMIs.FQFilter_zumis_nasalvasc_800cellag.benchmark.log:	Elapsed (wall clock) time (h:mm:ss or m:ss): 30:01.92
zumis_800cell_logs/zUMIs.FQFilter_zumis_nasalvasc_800cellah.benchmark.log:	Elapsed (wall clock) time (h:mm:ss or m:ss): 30:43.14
zumis_800cell_logs/zUMIs.FQFilter_zumis_nasalvasc_800cellai.benchmark.log:	Elapsed (wall clock) time (h:mm:ss or m:ss): 22:57.35
zumis_800cell_logs/zUMIs.Mapping.benchmark.log:	Elapsed (wall clock) time (h:mm:ss or m:ss): 2:30:57
zumis_800cell_logs/zUMIs.MergeBAM.benchmark.log:	Elapsed (wall clock) time (h:mm:ss or m:ss): 0:00.01
zumis_800cell_logs/zUMIs.Stats.benchmark.log:	Elapsed (wall clock) time (h:mm:ss or m:ss): 17:35.19
"""
)
```

In [34]:

```
z800_memory = parse_max_memory_usage(
"""
zumis_800cell_logs/zUMIs.BCDetection.benchmark.log:	Maximum resident set size (kbytes): 98232
zumis_800cell_logs/zUMIs.ConvertToLoom.benchmark.log:	Maximum resident set size (kbytes): 1445040
zumis_800cell_logs/zUMIs.Counting.benchmark.log:	Maximum resident set size (kbytes): 94019568
zumis_800cell_logs/zUMIs.FQFilter_zumis_nasalvasc_800cellaa.benchmark.log:	Maximum resident set size (kbytes): 60892
zumis_800cell_logs/zUMIs.FQFilter_zumis_nasalvasc_800cellab.benchmark.log:	Maximum resident set size (kbytes): 61108
zumis_800cell_logs/zUMIs.FQFilter_zumis_nasalvasc_800cellac.benchmark.log:	Maximum resident set size (kbytes): 60984
zumis_800cell_logs/zUMIs.FQFilter_zumis_nasalvasc_800cellad.benchmark.log:	Maximum resident set size (kbytes): 60892
zumis_800cell_logs/zUMIs.FQFilter_zumis_nasalvasc_800cellae.benchmark.log:	Maximum resident set size (kbytes): 60936
zumis_800cell_logs/zUMIs.FQFilter_zumis_nasalvasc_800cellaf.benchmark.log:	Maximum resident set size (kbytes): 60972
zumis_800cell_logs/zUMIs.FQFilter_zumis_nasalvasc_800cellag.benchmark.log:	Maximum resident set size (kbytes): 61108
zumis_800cell_logs/zUMIs.FQFilter_zumis_nasalvasc_800cellah.benchmark.log:	Maximum resident set size (kbytes): 61004
zumis_800cell_logs/zUMIs.FQFilter_zumis_nasalvasc_800cellai.benchmark.log:	Maximum resident set size (kbytes): 60804
zumis_800cell_logs/zUMIs.Mapping.benchmark.log:	Maximum resident set size (kbytes): 22200796
zumis_800cell_logs/zUMIs.MergeBAM.benchmark.log:	Maximum resident set size (kbytes): 3500
zumis_800cell_logs/zUMIs.Stats.benchmark.log:	Maximum resident set size (kbytes): 10896700
"""
)
```

In [ ]:

```

```

In [35]:

```
z800_times = {i.split('.')[1]:{'walltime':z800_walltime[i], 
                           'maxmem':z800_memory[i]} for i in z800_memory.keys()}
```

In [36]:

```
z800_times['remultiplex'] = {'walltime':walltime_to_seconds('5:22'), 'maxmem':98520}
```

In [37]:

```
fqsteps = [i for i in z800_times.keys() if i.startswith('FQFilter')]

z800_times['fqfilter'] = {
    'walltime':max([z800_times[i]['walltime'] for i in fqsteps]),
    'maxmem':sum([z800_times[i]['maxmem'] for i in fqsteps])
}

for i in fqsteps:
    del z800_times[i]
```

In [38]:

```
z800_times
```

Out[38]:

```
{'BCDetection': {'walltime': 0.91, 'maxmem': 98232},
 'ConvertToLoom': {'walltime': 42.45, 'maxmem': 1445040},
 'Counting': {'walltime': 5506.0, 'maxmem': 94019568},
 'Mapping': {'walltime': 9057.0, 'maxmem': 22200796},
 'MergeBAM': {'walltime': 0.01, 'maxmem': 3500},
 'Stats': {'walltime': 1055.19, 'maxmem': 10896700},
 'remultiplex': {'walltime': 323, 'maxmem': 98520},
 'fqfilter': {'walltime': 1843.14, 'maxmem': 548700}}
```

In [ ]:

```

```

### umite pipeline timing¶

as with zUMIs, the modified umite script above includes a benchmark wrapper which calls gnu time, producing step-named outputs in a log directory. Relevant lines from gnu time output are grepped from output logfiles as for zUMIs above, and used to compare the two pipelines. Disk space usage was assessed in the output directory using the unix program du, as described for zUMIs above.

In [ ]:

```

```

#### 200 cells¶

In [39]:

```
u200_time_total_s = 3022 # 13:33:32 - 14:23:54
```

In [40]:

```
u200_mem_total = 10699816 # disk space used (as above)
```

In [ ]:

```

```

In [41]:

```
u200_walltime = parse_elapsed_times(
"""
umicount_200cell_logs/umicount.GTF_parse_and_dump.benchmark.log:	Elapsed (wall clock) time (h:mm:ss or m:ss): 1:29.78
umicount_200cell_logs/umicount.STAR_alignments.benchmark.log:	Elapsed (wall clock) time (h:mm:ss or m:ss): 20:25.30
umicount_200cell_logs/umicount.load_STAR_genome.benchmark.log:	Elapsed (wall clock) time (h:mm:ss or m:ss): 0:35.05
umicount_200cell_logs/umicount.sort_bams.benchmark.log:	Elapsed (wall clock) time (h:mm:ss or m:ss): 2:00.60
umicount_200cell_logs/umicount.umicount.benchmark.log:	Elapsed (wall clock) time (h:mm:ss or m:ss): 13:19.44
umicount_200cell_logs/umicount.umiextract.benchmark.log:	Elapsed (wall clock) time (h:mm:ss or m:ss): 12:28.68
"""
)
```

In [42]:

```
u200_memory = parse_max_memory_usage(
"""
umicount_200cell_logs/umicount.GTF_parse_and_dump.benchmark.log:	Maximum resident set size (kbytes): 739912
umicount_200cell_logs/umicount.STAR_alignments.benchmark.log:	Maximum resident set size (kbytes): 13305800
umicount_200cell_logs/umicount.load_STAR_genome.benchmark.log:	Maximum resident set size (kbytes): 16512368
umicount_200cell_logs/umicount.sort_bams.benchmark.log:	Maximum resident set size (kbytes): 323616
umicount_200cell_logs/umicount.umicount.benchmark.log:	Maximum resident set size (kbytes): 946644
umicount_200cell_logs/umicount.umiextract.benchmark.log:	Maximum resident set size (kbytes): 101932
"""
)
```

In [ ]:

```

```

In [43]:

```
u200_times = {i.split('.')[1]:{'walltime':u200_walltime[i], 'maxmem':u200_memory[i]} for i in u200_memory.keys()}
```

In [44]:

```
u200_times
```

Out[44]:

```
{'GTF_parse_and_dump': {'walltime': 89.78, 'maxmem': 739912},
 'STAR_alignments': {'walltime': 1225.3, 'maxmem': 13305800},
 'load_STAR_genome': {'walltime': 35.05, 'maxmem': 16512368},
 'sort_bams': {'walltime': 120.6, 'maxmem': 323616},
 'umicount': {'walltime': 799.44, 'maxmem': 946644},
 'umiextract': {'walltime': 748.68, 'maxmem': 101932}}
```

In [ ]:

```

```

#### 400 cells¶

In [45]:

```
u400_time_total_s = 6000 # 14:23:54 - 16:03:54
```

In [46]:

```
u400_mem_total = 21845032
```

In [ ]:

```

```

In [47]:

```
u400_walltime = parse_elapsed_times(
"""
umicount_400cell_logs/umicount.GTF_parse_and_dump.benchmark.log:	Elapsed (wall clock) time (h:mm:ss or m:ss): 1:30.87
umicount_400cell_logs/umicount.STAR_alignments.benchmark.log:	Elapsed (wall clock) time (h:mm:ss or m:ss): 42:13.80
umicount_400cell_logs/umicount.load_STAR_genome.benchmark.log:	Elapsed (wall clock) time (h:mm:ss or m:ss): 0:15.02
umicount_400cell_logs/umicount.sort_bams.benchmark.log:	Elapsed (wall clock) time (h:mm:ss or m:ss): 4:08.50
umicount_400cell_logs/umicount.umicount.benchmark.log:	Elapsed (wall clock) time (h:mm:ss or m:ss): 26:27.50
umicount_400cell_logs/umicount.umiextract.benchmark.log:	Elapsed (wall clock) time (h:mm:ss or m:ss): 25:20.61
"""
)
```

In [48]:

```
u400_memory = parse_max_memory_usage(
"""
umicount_400cell_logs/umicount.GTF_parse_and_dump.benchmark.log:	Maximum resident set size (kbytes): 739808
umicount_400cell_logs/umicount.STAR_alignments.benchmark.log:	Maximum resident set size (kbytes): 13305780
umicount_400cell_logs/umicount.load_STAR_genome.benchmark.log:	Maximum resident set size (kbytes): 16512400
umicount_400cell_logs/umicount.sort_bams.benchmark.log:	Maximum resident set size (kbytes): 337272
umicount_400cell_logs/umicount.umicount.benchmark.log:	Maximum resident set size (kbytes): 946804
umicount_400cell_logs/umicount.umiextract.benchmark.log:	Maximum resident set size (kbytes): 102852
"""
)
```

In [ ]:

```

```

In [49]:

```
u400_times = {i.split('.')[1]:{'walltime':u400_walltime[i], 'maxmem':u400_memory[i]} for i in u400_memory.keys()}
```

In [50]:

```
u400_times
```

Out[50]:

```
{'GTF_parse_and_dump': {'walltime': 90.87, 'maxmem': 739808},
 'STAR_alignments': {'walltime': 2533.8, 'maxmem': 13305780},
 'load_STAR_genome': {'walltime': 15.02, 'maxmem': 16512400},
 'sort_bams': {'walltime': 248.5, 'maxmem': 337272},
 'umicount': {'walltime': 1587.5, 'maxmem': 946804},
 'umiextract': {'walltime': 1520.61, 'maxmem': 102852}}
```

In [ ]:

```

```

#### 800 cells¶

In [51]:

```
u800_time_total_s = 11771 # 16:03:54 - 19:20:05
```

In [52]:

```
u800_mem_total = 42604348
```

In [ ]:

```

```

In [53]:

```
u800_walltime = parse_elapsed_times(
"""
umicount_800cell_logs/umicount.GTF_parse_and_dump.benchmark.log:	Elapsed (wall clock) time (h:mm:ss or m:ss): 1:28.60
umicount_800cell_logs/umicount.STAR_alignments.benchmark.log:	Elapsed (wall clock) time (h:mm:ss or m:ss): 1:23:04
umicount_800cell_logs/umicount.load_STAR_genome.benchmark.log:	Elapsed (wall clock) time (h:mm:ss or m:ss): 0:14.97
umicount_800cell_logs/umicount.sort_bams.benchmark.log:	Elapsed (wall clock) time (h:mm:ss or m:ss): 7:58.99
umicount_800cell_logs/umicount.umicount.benchmark.log:	Elapsed (wall clock) time (h:mm:ss or m:ss): 52:28.29
umicount_800cell_logs/umicount.umiextract.benchmark.log:	Elapsed (wall clock) time (h:mm:ss or m:ss): 50:48.47
"""
)
```

In [54]:

```
u800_memory = parse_max_memory_usage(
"""
umicount_800cell_logs/umicount.GTF_parse_and_dump.benchmark.log:	Maximum resident set size (kbytes): 740016
umicount_800cell_logs/umicount.STAR_alignments.benchmark.log:	Maximum resident set size (kbytes): 13832792
umicount_800cell_logs/umicount.load_STAR_genome.benchmark.log:	Maximum resident set size (kbytes): 16512424
umicount_800cell_logs/umicount.sort_bams.benchmark.log:	Maximum resident set size (kbytes): 360244
umicount_800cell_logs/umicount.umicount.benchmark.log:	Maximum resident set size (kbytes): 947596
umicount_800cell_logs/umicount.umiextract.benchmark.log:	Maximum resident set size (kbytes): 103872
"""
)
```

In [ ]:

```

```

In [55]:

```
u800_times = {i.split('.')[1]:{'walltime':u800_walltime[i], 'maxmem':u800_memory[i]} for i in u800_memory.keys()}
```

In [56]:

```
u800_times
```

Out[56]:

```
{'GTF_parse_and_dump': {'walltime': 88.6, 'maxmem': 740016},
 'STAR_alignments': {'walltime': 4984.0, 'maxmem': 13832792},
 'load_STAR_genome': {'walltime': 14.97, 'maxmem': 16512424},
 'sort_bams': {'walltime': 478.99, 'maxmem': 360244},
 'umicount': {'walltime': 3148.29, 'maxmem': 947596},
 'umiextract': {'walltime': 3048.47, 'maxmem': 103872}}
```

In [ ]:

```

```

### resource usage plots¶

comparing summary statistics across cell-sets, first for all steps and then without counting alignment (since zUMIs does it twice)

In [467]:

```
f, axs = plt.subplots(3, 1, figsize=(3, 4))

axt, axm, axd = axs

zys = [z200_time_total_s, z400_time_total_s, z800_time_total_s]
axt.plot([1, 2, 3], zys, lw=3, c='cornflowerblue') 
axt.plot([1, 2, 3], zys, lw=5, c='k', zorder=-1) 

uys = [u200_time_total_s, u400_time_total_s, u800_time_total_s]
axt.plot([1, 2, 3], uys, lw=3, c='lightcoral') 
axt.plot([1, 2, 3], uys, lw=5, c='k', zorder=-1) 

for i in [1, 2, 3]:
    axt.plot([i, i], [zys[i-1], uys[i-1]], lw=2, ls=':', c='k', zorder=-1)

axt.set_yticklabels([round(float(i.get_text())/3600, 0) for i in axt.get_yticklabels()])
axt.set_xticks([1, 2, 3])
axt.set_xticklabels([200, 400, 800])

for n, i in enumerate([200, 400, 800]):
    axt.text(n+1, zys[n], '%.1fs' %(zys[n]/i))
    axt.text(n+1, uys[n], '%.1fs' %(uys[n]/i))
    axt.text(n+1, zys[n] * 0.5, '%.1fx' %(zys[n] / uys[n]))

axt.set_ylabel('Time (Hr)')

############################################

zys = [max([z200_times[i]['maxmem'] for i in z200_times]), 
       max([z400_times[i]['maxmem'] for i in z400_times]), 
       max([z800_times[i]['maxmem'] for i in z800_times])]

axm.plot([1, 2, 3], zys, lw=3, c='cornflowerblue') 
axm.plot([1, 2, 3], zys, lw=5, c='k', zorder=-1) 

uys = [max([u200_times[i]['maxmem'] for i in u200_times]), 
       max([u400_times[i]['maxmem'] for i in u400_times]), 
       max([u800_times[i]['maxmem'] for i in u800_times])]

axm.plot([1, 2, 3], uys, lw=3, c='lightcoral') 
axm.plot([1, 2, 3], uys, lw=5, c='k', zorder=-1) 

for i in [1, 2, 3]:
    axm.plot([i, i], [zys[i-1], uys[i-1]], lw=2, ls=':', c='k', zorder=-1)

axm.set_yticklabels([i.get_position()[1]/1e6 for i in axm.get_yticklabels()])
axm.set_xticks([1, 2, 3])
axm.set_xticklabels([200, 400, 800])

for n, i in enumerate([200, 400, 800]):
    axm.text(n+1, zys[n], '%.1fMb' %((zys[n]/1000)/i))
    axm.text(n+1, uys[n], '%.1fMb' %((uys[n]/1000)/i))
    axm.text(n+1, zys[n] * 0.5, '%.1fx' %(zys[n] / uys[n]))

axm.set_ylabel('Memory (Gb)')

############################################

zys = [z200_mem_total, z400_mem_total, z800_mem_total]
axd.plot([1, 2, 3], zys, lw=3, c='cornflowerblue') 
axd.plot([1, 2, 3], zys, lw=5, c='k', zorder=-1) 

uys = [u200_mem_total, u400_mem_total, u800_mem_total]
axd.plot([1, 2, 3], uys, lw=3, c='lightcoral') 
axd.plot([1, 2, 3], uys, lw=5, c='k', zorder=-1) 

for i in [1, 2, 3]:
    axd.plot([i, i], [zys[i-1], uys[i-1]], lw=2, ls=':', c='k', zorder=-1)

axd.set_yticklabels([i.get_position()[1]/1e6 for i in axm.get_yticklabels()])
axd.set_xticks([1, 2, 3])
axd.set_xticklabels([200, 400, 800])

for n, i in enumerate([200, 400, 800]):
    axd.text(n+1, zys[n], '%.1fMb' %((zys[n]/1000)/i))
    axd.text(n+1, uys[n], '%.1fMb' %((uys[n]/1000)/i))
    axd.text(n+1, zys[n] * 0.5, '%.1fx' %(zys[n] / uys[n]))

axd.set_ylabel('Disk (Gb)')

for ax in axs:
    sns.despine(ax=ax)

plt.tight_layout()
f.align_labels()
plt.savefig('XX_output/figures/benchmark_v2_stats_by_cellcount.svg', format='svg')
plt.show()
```

In [ ]:

```

```

In [468]:

```
f, axs = plt.subplots(3, 1, figsize=(3, 4))

axt, axm, axd = axs

zmap = ['Mapping']
umap = ['load_STAR_genome', 'STAR_alignments']

zys = [z200_time_total_s - z200_times['Mapping']['walltime'], 
       z400_time_total_s - z400_times['Mapping']['walltime'], 
       z800_time_total_s - z800_times['Mapping']['walltime']]

axt.plot([1, 2, 3], zys, lw=3, c='cornflowerblue') 
axt.plot([1, 2, 3], zys, lw=5, c='k', zorder=-1) 

uys = [u200_time_total_s - sum([u200_times[i]['walltime'] for i in umap]), 
       u400_time_total_s - sum([u400_times[i]['walltime'] for i in umap]), 
       u800_time_total_s - sum([u800_times[i]['walltime'] for i in umap]), ]
axt.plot([1, 2, 3], uys, lw=3, c='lightcoral') 
axt.plot([1, 2, 3], uys, lw=5, c='k', zorder=-1) 

for i in [1, 2, 3]:
    axt.plot([i, i], [zys[i-1], uys[i-1]], lw=2, ls=':', c='k', zorder=-1)

axt.set_yticklabels([round(float(i.get_text())/3600, 0) for i in axt.get_yticklabels()])
axt.set_xticks([1, 2, 3])
axt.set_xticklabels([200, 400, 800])

for n, i in enumerate([200, 400, 800]):
    axt.text(n+1, zys[n], '%.1fs' %(zys[n]/i))
    axt.text(n+1, uys[n], '%.1fs' %(uys[n]/i))
    axt.text(n+1, zys[n] * 0.5, '%.1fx' %(zys[n] / uys[n]))

axt.set_ylabel('Time (Hr)')

############################################

zys = [max([z200_times[i]['maxmem'] for i in z200_times if i not in zmap]), 
       max([z400_times[i]['maxmem'] for i in z400_times if i not in zmap]), 
       max([z800_times[i]['maxmem'] for i in z800_times if i not in zmap])]

axm.plot([1, 2, 3], zys, lw=3, c='cornflowerblue') 
axm.plot([1, 2, 3], zys, lw=5, c='k', zorder=-1) 

uys = [max([u200_times[i]['maxmem'] for i in u200_times if i not in umap]), 
       max([u400_times[i]['maxmem'] for i in u400_times if i not in umap]), 
       max([u800_times[i]['maxmem'] for i in u800_times if i not in umap])]

axm.plot([1, 2, 3], uys, lw=3, c='lightcoral') 
axm.plot([1, 2, 3], uys, lw=5, c='k', zorder=-1) 

for i in [1, 2, 3]:
    axm.plot([i, i], [zys[i-1], uys[i-1]], lw=2, ls=':', c='k', zorder=-1)

axm.set_yticklabels([i.get_position()[1]/1e6 for i in axm.get_yticklabels()])
axm.set_xticks([1, 2, 3])
axm.set_xticklabels([200, 400, 800])

for n, i in enumerate([200, 400, 800]):
    axm.text(n+1, zys[n], '%.1fMb' %((zys[n]/1000)/i))
    axm.text(n+1, uys[n], '%.1fMb' %((uys[n]/1000)/i))
    axm.text(n+1, zys[n] * 0.5, '%.1fx' %(zys[n] / uys[n]))

axm.set_ylabel('Memory (Gb)')

############################################

axd.set_axis_off()

for ax in axs:
    sns.despine(ax=ax)

plt.tight_layout()
f.align_labels()
plt.savefig('XX_output/figures/benchmark_v2_stats_by_cellcount_noalign.svg', format='svg')
plt.show()
```

In [ ]:

```

```

now to represent individual stages as rectangles in a 2D axis of time and memory:

In [59]:

```
z800_times
```

Out[59]:

```
{'BCDetection': {'walltime': 0.91, 'maxmem': 98232},
 'ConvertToLoom': {'walltime': 42.45, 'maxmem': 1445040},
 'Counting': {'walltime': 5506.0, 'maxmem': 94019568},
 'Mapping': {'walltime': 9057.0, 'maxmem': 22200796},
 'MergeBAM': {'walltime': 0.01, 'maxmem': 3500},
 'Stats': {'walltime': 1055.19, 'maxmem': 10896700},
 'remultiplex': {'walltime': 323, 'maxmem': 98520},
 'fqfilter': {'walltime': 1843.14, 'maxmem': 548700}}
```

In [60]:

```
u800_times
```

Out[60]:

```
{'GTF_parse_and_dump': {'walltime': 88.6, 'maxmem': 740016},
 'STAR_alignments': {'walltime': 4984.0, 'maxmem': 13832792},
 'load_STAR_genome': {'walltime': 14.97, 'maxmem': 16512424},
 'sort_bams': {'walltime': 478.99, 'maxmem': 360244},
 'umicount': {'walltime': 3148.29, 'maxmem': 947596},
 'umiextract': {'walltime': 3048.47, 'maxmem': 103872}}
```

In [ ]:

```

```

need to map stages between pipelines, here by index within these two arrays:

In [61]:

```
ordermapping = [
    ['remultiplex', 'fqfilter', 'Mapping', None, None, 'Counting', 'Stats', 'rest'],
    [None, 'umiextract', 'STAR_alignments', 'sort_bams', 'GTF_parse_and_dump', 'umicount', None, 'rest']
]
```

In [469]:

```
f = plt.figure(figsize=(7.5, 5))
grid = f.add_gridspec(ncols=2, nrows=1, 
         width_ratios=[1, 1], wspace=0.2)

import matplotlib.patches as patches

ax1 = f.add_subplot(grid[0])
ax2 = f.add_subplot(grid[1], sharey=ax1)

memscale = 1000 # to mB

### ax1: zUMIs
ypos = 0
xmax = 0
xbase = 5e6

ax1.axvline(xbase/memscale, c='dimgray', ls=':', zorder=-1)

for n, i in enumerate(ordermapping[0]):
    
    if i:
        if i == 'rest':
            continue
            pmem = xbase / memscale
            ptim = z800_time_total_s - sum([z800_times[i]['walltime'] for i in ordermapping[0] \
                                        if i not in [None, 'remultiplex', 'rest']])
        else:
            pmem = (xbase + int(z800_times[i]['maxmem'])) / memscale
            ptim = int(z800_times[i]['walltime'])

        rect = patches.Rectangle((0, ypos), width=pmem, height=ptim, linewidth=1,
                                 edgecolor='k', facecolor=sns.color_palette('muted')[n], alpha=0.5)

        ax1.add_patch(rect)
        
        ax1.text(xbase / memscale, ypos + (0.5 * ptim), i, ha='right')

        ypos += ptim
        xmax = max(xmax, pmem)
                
ax1.set_ylim(0, ypos * 1.05 )
ax1.set_xlim(0, xmax * 1.05 )

storelab = ax1.get_xticklabels()
ax1.set_xticks([i + xbase/memscale for i in ax1.get_xticks()])
ax1.set_xticklabels([int((i/memscale)-(xbase/1e6)) for i in ax1.get_xticks()])

sns.despine(ax=ax1, left=True, top=True, right=False, bottom=False)

ax1.xaxis.set_inverted(True)
ax1.yaxis.tick_right()

### ax2: umicount
ypos = 0
xmax = 0
xbase = 5e6

ax2.axvline(xbase/memscale, c='dimgray', ls=':', zorder=-1)

for n, i in enumerate(ordermapping[1]):
    
    if i:
        if i == 'rest':
            pmem = xbase / memscale
            ptim = u800_time_total_s - sum([u800_times[i]['walltime'] for i in ordermapping[1] \
                                        if i not in [None, 'rest']])
        else:
            pmem = (xbase + int(u800_times[i]['maxmem'])) / memscale
            ptim = int(u800_times[i]['walltime'])

        rect = patches.Rectangle((0, ypos), width=pmem, height=ptim, linewidth=1,
                                 edgecolor='k', facecolor=sns.color_palette('muted')[n], alpha=0.5)

        ax2.add_patch(rect)
        
        ax2.text(xbase / memscale, ypos + (0.5 * ptim), i, ha='left')

        ypos += ptim
        xmax = max(xmax, pmem)
        
# ax2.set_ylim(0, ypos * 1.05 )
# ax2.set_xlim(0, xmax * 1.05 )

ax2.set_xticks(ax1.get_xticks())
ax2.set_xticklabels(storelab)

sns.despine(ax=ax2, left=False, top=True, right=True, bottom=False)

plt.tight_layout()
plt.savefig('XX_output/figures/benchmark_v2_800cell_statscomp.svg', format='svg')
plt.show()
```

In [ ]:

```

```

also plot fold changes in resource usage as bar plots

In [470]:

```
f = plt.figure(figsize=(8, 2))
grid = f.add_gridspec(ncols=5, nrows=1,
         width_ratios=[3, 2, 2, 2, 2], wspace=0.1)

ax1 = f.add_subplot(grid[0,0])
ax2 = f.add_subplot(grid[0,1], sharey=ax1)
ax3 = f.add_subplot(grid[0,2], sharey=ax1)
ax4 = f.add_subplot(grid[0,3], sharey=ax1)
ax5 = f.add_subplot(grid[0,4], sharey=ax1)

ax1.set_ylim(-2, 10)
wid = 0.66

#### overall 

timr = z800_time_total_s / u800_time_total_s

ax1.bar(0, height=timr, width=wid, color='lightgray', ec='k', lw=0.5)
ax1.text(0, timr, '%.1fx' %timr, ha='center', va='bottom')

memr = max([int(z800_times[i]['maxmem']) for i in z800_times.keys()]) / \
       max([int(u800_times[i]['maxmem']) for i in u800_times.keys()])
    
ax1.bar(1, height=memr, width=wid, color='dimgray', ec='k', lw=0.5)
ax1.text(1, memr, '%.1fx' %memr, ha='center', va='bottom')

disr = z800_mem_total / u800_mem_total

ax1.bar(2, height=disr, width=wid, color='w', ec='k', lw=0.5)
ax1.text(2, disr, '%.1fx' %disr, ha='center', va='bottom')

ax1.set_xticks([0, 1, 2])
ax1.set_xticklabels(['Time', 'Memory', 'Disk'])
ax1.set_ylabel('umicount\nimprovement')

#### umiextract

timr = -(u800_times['umiextract']['walltime'] / z800_times['fqfilter']['walltime'])

ax3.bar(0, height=timr, width=wid, color='lightgray', ec='k', lw=0.5)
ax3.text(0, max(0, timr), '%.1fx' %timr, ha='center', va='bottom')

memr = z800_times['fqfilter']['maxmem'] / u800_times['umiextract']['maxmem']
    
ax3.bar(1, height=memr, width=wid, color='dimgray', ec='k', lw=0.5)
ax3.text(1, min(memr, 10), '%.1fx' %memr, ha='center', va='bottom')

#### mapping

timr = z800_times['Mapping']['walltime'] / \
       (u800_times['load_STAR_genome']['walltime'] + u800_times['STAR_alignments']['walltime'])

ax4.bar(0, height=timr, width=wid, color='lightgray', ec='k', lw=0.5)
ax4.text(0, max(0, timr), '%.1fx' %timr, ha='center', va='bottom')

memr = z800_times['Mapping']['maxmem'] / u800_times['STAR_alignments']['maxmem']
    
ax4.bar(1, height=memr, width=wid, color='dimgray', ec='k', lw=0.5)
ax4.text(1, min(memr, 10), '%.1fx' %memr, ha='center', va='bottom')

#### counting

timr = z800_times['Counting']['walltime'] / u800_times['umicount']['walltime']

ax5.bar(0, height=timr, width=wid, color='lightgray', ec='k', lw=0.5)
ax5.text(0, max(0, timr), '%.1fx' %timr, ha='center', va='bottom')

memr = z800_times['Counting']['maxmem'] / u800_times['umicount']['maxmem']
    
ax5.bar(1, height=memr, width=wid, color='dimgray', ec='k', lw=0.5)
ax5.text(1, min(memr, 10), '%.1fx' %memr, ha='center', va='bottom')


for ax in [ax1, ax2, ax3, ax4, ax5]:
    sns.despine(ax=ax, bottom=True)
    ax.axhline(0, c='k', lw=0.5)
    
    if ax != ax1:
        ax.set_xticks([])
        ax.set_xticklabels('')
        ax.set_yticklabels('')
        ax.set_xlim(-0.5, 1.5)


plt.tight_layout()
plt.savefig('XX_output/figures/benchmark_v2_800cell_stats_by_stage.svg', format='svg')
plt.show()
```

In [ ]:

```

```

## compare count tables¶

read counts by category in zUMIs and umite, match categories and cells, and compare quantifications

In [ ]:

```

```

### parse counts¶

In [67]:

```
countsdir = '/mnt/volume/resources/external_SS3_cells/2nd_nasal_dataset_complete_bench/count_tables_800cell'
```

In [ ]:

```

```

zumis

zumis counts require preprocessing from R objects (the call here is not exactly the one used but it will be effectively identical)

In [ ]:

```
obj <- readRDS("/mnt/volume/jupyter/projects/manuscript_prep/11_umicounts_benchmarking/XX_input/bench_data_nasopharyngeal/zumis_vs_umicounts_200cell/countables_zumis/expression/zumis_nasopharyngeal_200cell.dgecounts.rds")

write.table(as.matrix(obj$umicount$exon$all), '/home/rstudio/zumis_nasopharyngeal_counts.umi_exon.mat',quote=F,sep=",")
write.table(as.matrix(obj$umicount$intron$all), '/home/rstudio/zumis_nasopharyngeal_counts.umi_intron.mat',quote=F,sep=",")
write.table(as.matrix(obj$readcount_internal$intron$all), '/home/rstudio/zumis_nasopharyngeal_counts.read_intron.mat',quote=F,sep=",")
write.table(as.matrix(obj$readcount_internal$exon$all), '/home/rstudio/zumis_nasopharyngeal_counts.read_exon.mat',quote=F,sep=",")
write.table(as.matrix(obj$readcount$exon$all), '/home/rstudio/zumis_nasopharyngeal_counts.readall_exon.mat',quote=F,sep=",")
write.table(as.matrix(obj$readcount$intron$all), '/home/rstudio/zumis_nasopharyngeal_counts.readall_intron.mat',quote=F,sep=",")
```

In [115]:

```
zumicounts = {
    'umiE':pd.read_csv(countsdir + '/zumis_800cell_counts.umi_exon.mat'),
    'umiI':pd.read_csv(countsdir + '/zumis_800cell_counts.umi_intron.mat'),
    'intE':pd.read_csv(countsdir + '/zumis_800cell_counts.read_exon.mat'),
    'intI':pd.read_csv(countsdir + '/zumis_800cell_counts.read_intron.mat'),
    'readE':pd.read_csv(countsdir + '/zumis_800cell_counts.readall_exon.mat'),
    'readI':pd.read_csv(countsdir + '/zumis_800cell_counts.readall_intron.mat'),
}
```

In [ ]:

```

```

In [116]:

```
geneidx = []
for i in zumicounts:
    geneidx += zumicounts[i].index.tolist()
geneidx = list(set(geneidx))
```

In [117]:

```
len(geneidx)
```

Out[117]:

```
27877
```

In [ ]:

```

```

In [118]:

```
for i in zumicounts:
    zumicounts[i] = zumicounts[i].reindex(geneidx).fillna(0)
```

In [ ]:

```

```

umicount

In [72]:

```
countcols = ['UE', 'UI', 'RE', 'RI', 'D']
```

In [79]:

```
umicounts = {
    'umiE':pd.read_csv(countsdir + '/umicounts.UE.tsv', sep='\t', index_col=0).T,
    'umiI':pd.read_csv(countsdir + '/umicounts.UI.tsv', sep='\t', index_col=0).T,
    'intE':pd.read_csv(countsdir + '/umicounts.RE.tsv', sep='\t', index_col=0).T,
    'intI':pd.read_csv(countsdir + '/umicounts.RI.tsv', sep='\t', index_col=0).T,
    'dupe':pd.read_csv(countsdir + '/umicounts.D.tsv', sep='\t', index_col=0).T
}
```

In [91]:

```
for i in umicounts:
    p = umicounts[i]
    p.columns = [i.split('_')[0] for i in p.columns]
    umicounts[i] = p
```

In [ ]:

```

```

In [92]:

```
geneshare = [i for i in umicounts['umiE'].index if i in zumicounts['umiE'].index]
len(geneshare)
```

Out[92]:

```
27877
```

In [93]:

```
cellorder = sorted(umicounts['umiE'].columns.tolist())
len(cellorder)
```

Out[93]:

```
800
```

In [ ]:

```

```

### map zUMIs barcode IDs¶

zUMIs uses internal IDs for barcodes (assigned in remultiplex) so need to map these back to SRR IDs

In [120]:

```
zumis_samplemap = pd.read_csv('/mnt/volume/resources/external_SS3_cells/2nd_nasal_dataset_complete_bench/benchmark_800cell/reads_for_zUMIs.samples.txt', sep='\t')
```

In [121]:

```
zumis_samplemap
```

Out[121]:

|  | r1 | r2 | sample | BC |
| --- | --- | --- | --- | --- |
| 0 | SRR19884905\_1.fastq.gz | SRR19884905\_2.fastq.gz | SRR19884905 | VFPHIXEA |
| 1 | SRR19884906\_1.fastq.gz | SRR19884906\_2.fastq.gz | SRR19884906 | GIVAMKSP |
| 2 | SRR19884912\_1.fastq.gz | SRR19884912\_2.fastq.gz | SRR19884912 | LOOXWQTH |
| 3 | SRR19884913\_1.fastq.gz | SRR19884913\_2.fastq.gz | SRR19884913 | PGWKJKED |
| 4 | SRR19884915\_1.fastq.gz | SRR19884915\_2.fastq.gz | SRR19884915 | NPLGRUHT |
| ... | ... | ... | ... | ... |
| 795 | SRR19886338\_1.fastq.gz | SRR19886338\_2.fastq.gz | SRR19886338 | IJTSDWEU |
| 796 | SRR19886341\_1.fastq.gz | SRR19886341\_2.fastq.gz | SRR19886341 | NESZVUZZ |
| 797 | SRR19886342\_1.fastq.gz | SRR19886342\_2.fastq.gz | SRR19886342 | RISYBNTX |
| 798 | SRR19886343\_1.fastq.gz | SRR19886343\_2.fastq.gz | SRR19886343 | ATOLMYXN |
| 799 | SRR19886344\_1.fastq.gz | SRR19886344\_2.fastq.gz | SRR19886344 | VNVKSTCK |

800 rows × 4 columns

In [ ]:

```

```

In [122]:

```
zumis_samplemap = zumis_samplemap[['sample', 'BC']].set_index('BC')['sample'].to_dict()
```

In [123]:

```
for i in zumicounts.keys():
    zumicounts[i].columns = zumicounts[i].columns.map(zumis_samplemap)
```

In [ ]:

```

```

### remove cells filtered by zUMIs¶

for some reason zUMIs removes some cells during processing, making them impossible to compare. as such, we are forced to remove these cells from the umicounts table as well

In [125]:

```
zumis_filt_cells = [i for i in umicounts['umiE'].columns if i not in zumicounts['umiE'].columns]
len(zumis_filt_cells)
```

Out[125]:

```
236
```

In [ ]:

```

```

In [126]:

```
ucountsum = pd.concat([umicounts[i].sum() for i in umicounts], axis=1)
```

In [127]:

```
ucountsum.columns = umicounts.keys()
```

In [128]:

```
ucountsum['inz'] = True
ucountsum.loc[zumis_filt_cells, 'inz'] = False
```

In [129]:

```
ucountsum
```

Out[129]:

|  | umiE | umiI | intE | intI | dupe | inz |
| --- | --- | --- | --- | --- | --- | --- |
| SRR19886105 | 38930 | 2603 | 184897 | 16677 | 145936 | False |
| SRR19885277 | 44286 | 3658 | 103787 | 12470 | 184971 | True |
| SRR19885062 | 31011 | 1538 | 158391 | 11218 | 153900 | True |
| SRR19885087 | 42639 | 2262 | 130894 | 8942 | 163002 | True |
| SRR19885080 | 45230 | 2496 | 141312 | 10223 | 157956 | True |
| ... | ... | ... | ... | ... | ... | ... |
| SRR19885076 | 9095 | 647 | 4859 | 1480 | 1576 | True |
| SRR19885855 | 2195 | 47 | 10460 | 1120 | 2629 | True |
| SRR19884929 | 3619 | 206 | 11113 | 1902 | 1036 | True |
| SRR19885941 | 5799 | 275 | 5760 | 1668 | 649 | False |
| SRR19886248 | 3477 | 86 | 6725 | 1994 | 759 | False |

800 rows × 6 columns

In [ ]:

```

```

### counts spearman correlations¶

In [214]:

```
from scipy.stats import spearmanr
```

In [229]:

```
cellspear = pd.DataFrame(index=cellorder, columns=['umiE', 'umiI', 'intE', 'intI'])
```

In [231]:

```
for i in cellorder:
    if i in zumis_filt_cells: continue
    for j in cellspear.columns:
        cellspear.at[i, j] = spearmanr(zumicounts[j].loc[geneshare, i],
                                       umicounts[j].loc[geneshare, i])[0]
```

In [ ]:

```

```

In [232]:

```
cellspear['mqcpass'] = cellspear.index.isin(fmqcdata.index.unique())
```

In [233]:

```
cellspear.dropna()['mqcpass'].value_counts()
```

Out[233]:

```
mqcpass
True     368
False    196
Name: count, dtype: int64
```

In [234]:

```
cellspear.groupby('mqcpass').mean()
```

Out[234]:

|  | umiE | umiI | intE | intI |
| --- | --- | --- | --- | --- |
| mqcpass |  |  |  |  |
| False | 0.953 | 0.940 | 0.964 | 0.907 |
| True | 0.963 | 0.952 | 0.971 | 0.937 |

In [ ]:

```

```

In [235]:

```
fcellspear = cellspear[cellspear['mqcpass'] == True].dropna()
```

In [471]:

```
f = plt.figure(figsize=(3, 2.5))
ax = f.gca()

p = fcellspear.iloc[:, :4]
plt.barh(p.columns, p.mean(), xerr=p.std(), 
         align='center', color='lightgray', ec='k', lw=0.5, capsize=5)

#ax.axvline(0.95, c='lightgray', ls=':', zorder=-1)

sns.despine(ax=ax)

ax.set_xlabel('zUMIs vs umicount\nSpearman correlation')

plt.tight_layout()
plt.savefig('XX_output/figures/benchmark_v2_umicount_zumi_countcorr.svg', format='svg')
plt.show()
```

In [ ]:

```

```

### to UMAP¶

In [152]:

```
udata = sc.AnnData(umicounts['umiE'].loc[geneshare, fcellcorr.index].T)
udata
```

Out[152]:

```
AnnData object with n_obs × n_vars = 368 × 27877
```

In [153]:

```
zdata = sc.AnnData(zumicounts['umiE'].loc[geneshare, fcellcorr.index].T)
zdata
```

Out[153]:

```
AnnData object with n_obs × n_vars = 368 × 27877
```

In [ ]:

```

```

map geneIDs to gene name using an ensembl lookup table i had on disk

In [154]:

```
orthos = pd.read_csv('/mnt/volume/jupyter/projects/oguzhan_wnt_reporter/datasets/T6_C2WO_and_C21R_SS3_singlecells-oct20/XX_input/ensembl102_mouse_human_orthologs.tsv', sep = '\t')
```

In [155]:

```
ensIDmap = orthos[['Gene stable ID', 'Gene name']].set_index('Gene stable ID')['Gene name'].to_dict()
geneshare_geneid = [ensIDmap[i] if i in ensIDmap else 'X' for i in geneshare]
```

In [156]:

```
assert (udata.var.index == geneshare).all()
udata.var.index = geneshare_geneid
udata = udata[:, udata.var.index != 'X']
```

In [157]:

```
assert (zdata.var.index == geneshare).all()
zdata.var.index = geneshare_geneid
zdata = zdata[:, zdata.var.index != 'X']
```

In [ ]:

```

```

consistent preprocessing

In [158]:

```
for ad in [udata, zdata]:
    sc.pp.filter_cells(ad, min_genes=0)
    sc.pp.filter_genes(ad, min_cells=0)
    ad.obs['n_counts'] = ad.X.sum(1)
    
    sc.pp.normalize_total(ad, target_sum=ad.obs['n_counts'].mean())
    sc.pp.log1p(ad)
```

In [159]:

```
sc.pp.highly_variable_genes(udata, n_top_genes=2000, min_mean=0.1)
sc.pp.highly_variable_genes(zdata, n_top_genes=2000, min_mean=0.1)
```

In [ ]:

```

```

use shared highly variable genes for embedding

In [160]:

```
hvgshare = pd.concat([udata.var['highly_variable'].rename('u'), 
                      zdata.var['highly_variable'].rename('z')], axis=1)
```

In [161]:

```
hvgshare = hvgshare[hvgshare.sum(1) == 2].index.tolist()
len(hvgshare)
```

Out[161]:

```
1528
```

In [ ]:

```

```

consistent umap embedding

In [162]:

```
for ad in [udata, zdata]:
    sc.tl.pca(ad, svd_solver='arpack', n_comps=10)
    sc.pp.neighbors(ad, n_neighbors=20, n_pcs=5)
    sc.tl.umap(ad, min_dist=0.5, spread=1)
```

In [518]:

```
f, axs = plt.subplots(1, 2, figsize=(4, 2))

ax1, ax2 = axs

s = 80

p = knndist.sort_values('jacc')
sc = ax1.scatter(p['u_u1'], p['u_u2']*-1, c='w',
                 ec='k', lw=2, s=s,
                 rasterized=True)

p1 = p[p['u_u1'] <= 4]
p2 = p[p['u_u1'] >= 4]
for n, pp in enumerate([p1, p2]):
    sc = ax1.scatter(pp['u_u1'], pp['u_u2']*-1, 
                     c=['lightgreen', 'lightskyblue'][n],
                     ec='k', lw=0, s=s/2, alpha=1,
                     rasterized=True)

p = knndist.sort_values('jacc')
ax2.scatter(p['z_u1'], p['z_u2'], c='w',
            ec='k', lw=2, s=s, 
            rasterized=True)

p1 = p[p['z_u1'] <= 7]
p2 = p[p['z_u1'] >= 7]
for n, pp in enumerate([p1, p2]):
    sc = ax2.scatter(pp['z_u1'], pp['z_u2'], 
                     c=['lightgreen', 'lightskyblue'][n],
                     ec='k', lw=0, s=s/2,
                     rasterized=True)

for ax in axs:
    ax.set_xticks([])
    ax.set_yticks([])

plt.tight_layout()
f.align_labels()
plt.savefig('XX_output/figures/benchmark_v2_umap_colored.svg', format='svg')
plt.show()
```

In [ ]:

```

```

#### compare neighborhoods¶

compare similarity of cell neighborhoods in knn underlying umap embedding

In [170]:

```
uconn = pd.DataFrame(udata.obsp['connectivities'].todense(),
                     index=fcellcorr.index, columns=fcellcorr.index)

zconn = pd.DataFrame(zdata.obsp['connectivities'].todense(),
                     index=fcellcorr.index, columns=fcellcorr.index)
```

In [ ]:

```

```

In [171]:

```
knndist = pd.DataFrame(index=fcellcorr.index)
```

In [172]:

```
from scipy.spatial.distance import euclidean
```

In [173]:

```
for i in fcellcorr.index:
    pu = uconn.loc[i]
    pz = zconn.loc[i]
    
    knndist.at[i, 'corr'] = pearsonr(pu, pz)[0]
    knndist.at[i, 'eucl'] = euclidean(pu, pz)
```

In [174]:

```
knndist['corr'].describe()
```

Out[174]:

```
count   368.000
mean      0.928
std       0.053
min       0.610
25%       0.909
50%       0.943
75%       0.963
max       0.993
Name: corr, dtype: float64
```

In [ ]:

```

```

In [175]:

```
for i in fcellcorr.index:
    pu = set([idx for idx, i in (uconn.loc[i] > 0).items() if i])
    pz = set([idx for idx, i in (zconn.loc[i] > 0).items() if i])
    
    intersection = len(pu.intersection(pz))
    union = len(pu.union(pz))
    knndist.at[i, 'jacc'] = intersection / union
```

In [176]:

```
knndist['jacc'].describe()
```

Out[176]:

```
count   368.000
mean      0.803
std       0.095
min       0.514
25%       0.739
50%       0.810
75%       0.865
max       1.000
Name: jacc, dtype: float64
```

In [ ]:

```

```

In [472]:

```
f = plt.figure(figsize=(4, 2.5))
ax = f.gca()

sns.kdeplot(knndist['jacc'], color='lightcoral', fill=True)

sns.despine(ax=ax)

plt.xlim(0, 1.05)

from mpl_toolkits.axes_grid1.inset_locator import inset_axes
axins = inset_axes(ax, width='3%', height='30%', borderpad=0, loc=3,
                   bbox_to_anchor=(0.29, 0.69, 1, 1), bbox_transform=ax.transAxes)

cb = f.colorbar(sc, cax=axins)
cb.set_label('Jaccard', fontsize=10, rotation=90, labelpad=-45)
cb.set_ticks([0.5, 1])

plt.tight_layout()
plt.savefig('XX_output/figures/benchmark_v2_umap_knn_jaccard_density.svg', format='svg')
plt.show()
```

In [ ]:

```

```

In [549]:

```
f = plt.figure(figsize=(2, 2.5))
ax = f.gca()

plt.boxplot(knndist['jacc'], showfliers=False)

sns.despine(ax=ax, bottom=True)
plt.text(1, 0.5, '%.2f' %knndist['jacc'].median())

plt.xticks([])
plt.yticks([0, 0.5, 1])

plt.ylim(0, 1)
plt.xlim(0.75, 1.5)

plt.ylabel('Jacc')

plt.tight_layout()
plt.savefig('XX_output/figures/benchmark_v2_umap_knn_jaccard_density_boxplot.svg', format='svg')
plt.show()
```

In [ ]:

```

```

### explore use of duplicate counts¶

In [250]:

```
dcounts = umicounts['dupe'].drop(index=[i for i in umicounts['dupe'].index if i.startswith('_')])
```

In [251]:

```
dcounts = dcounts[dcounts.sum(1) > 0]
```

In [252]:

```
dcounts.sum(1).value_counts()[:10]
```

Out[252]:

```
1     1430
2      911
3      691
4      521
5      476
6      384
7      315
8      315
9      290
10     228
Name: count, dtype: int64
```

In [ ]:

```

```

collate information on total reads, umis, and dupes

In [270]:

```
dstats = pd.DataFrame(dcounts.sum(), columns=['D'])
```

In [283]:

```
datadir = '/mnt/volume/resources/external_SS3_cells/2nd_nasal_dataset_complete_bench'
fqsum_800cell = 0
with open(datadir + '/reads_per_cell.grep', 'r') as f:
    for i in f.readlines():
        _, fqnum = i.split(':')
        dstats.at[_.split('_1')[0].split('/')[-1], 'reads'] = int(fqnum)
```

In [286]:

```
dstats = dstats.astype('int64')
```

In [ ]:

```
dstats['U'] = umicounts['umiE'].drop(index=[i for i in umicounts['umiE'].index if i.startswith('_')]).sum() + \
              umicounts['umiI'].drop(index=[i for i in umicounts['umiI'].index if i.startswith('_')]).sum()
```

In [288]:

```
dstats['pdupe'] = dstats['D'] / dstats['reads']
```

In [337]:

```
dstats['mqcpass'] = dstats.index.isin(fmqcdata.index.unique())
```

In [ ]:

```

```

compute saturation metric as in 10X:

In [428]:

```
dstats['sat'] = 1 - (dstats['U'] / (dstats['U'] + dstats['D']))
```

In [ ]:

```

```

In [435]:

```
loesfit = loess(dstats['reads'], dstats['sat'], span=0.3, degree=1)
loesfit.fit()
```

In [436]:

```
loesfit_x = np.linspace(dstats['reads'].min() + 1, dstats['reads'].max() - 1, 20)
loesfit_y = loesfit.predict(loesfit_x, stderror=True)
```

In [473]:

```
f = plt.figure(figsize=(3, 3))
ax = f.gca()

p = dstats.sort_values('U')
plt.scatter(p['reads'], p['sat'], c='lightgray', ec='k', lw=0.5, rasterized=True)

plt.plot(loesfit_x, loesfit_y.values, c='r', lw=2)
# plt.fill_between(loesfit_x, 
#                  loesfit_y.values - (loesfit_y.stderr / 2),
#                  loesfit_y.values + (loesfit_y.stderr / 2),
#                  color='lightcoral', alpha=0.8)

plt.xticks([5e4, 25e4, 50e4], ['50k', '250k', '500k'])
plt.xlim(0, 55e4)

plt.xlabel('Total reads')
plt.ylabel('Saturation (U / (U+D))')

sns.despine(ax=ax)

plt.tight_layout()
plt.savefig('XX_output/figures/benchmark_v2_saturation_from_umidupes.svg', format='svg')
plt.show()
```

In [ ]:

```

```

## generalization on 2nd T-cell dataset¶

in response to reviewer2: "1. All results are derived from a single dataset (GSE207085). To strengthen claims of scalability and generality, the authors should consider including an additional dataset (e.g., with different read lengths or sequencing error profiles)."

In [ ]:

```

```

### SRA download¶

SRA accessions from https://www.ncbi.nlm.nih.gov/Traces/study/?acc=PRJNA1129044 via
https://www.ncbi.nlm.nih.gov/geo/query/acc.cgi?acc=GSE270928

In [ ]:

```
wc -l SRA_accessions.txt: 6416 SRA_accessions.txt
```

In [ ]:

```
cat SRA_accessions.txt | shuf | head -n 500 | sort > accessions_shuf_500.txt # 500 random cells
```

In [ ]:

```
cat accessions_shuf_500.txt | parallel $CO/sratoolkit.2.10.5-ubuntu64/bin/prefetch {}
```

In [ ]:

```

```

In [ ]:

```
# check all files were downloaded
ls -1 -d */ | rev | cut -c 2- | rev | sort > accesions_downloaded.txt
diff -b accesions_downloaded.txt accessions_full.sort.txt | grep '^>' | cut -c 3- > accessions_missed.txt
```

In [ ]:

```
cat accessions_missed.txt | parallel $CO/sratoolkit.2.10.5-ubuntu64/bin/prefetch {}
```

In [ ]:

```

```

In [ ]:

```
find ./ -name '*.sra' | xargs -I % echo /home/ubuntu/dev/co/sratoolkit.2.10.5-ubuntu64/bin/fastq-dump --origfmt --defline-qual '+' --gzip --split-files %> SRA_fastq_dump_calls.sh
```

In [ ]:

```
parallel < ./SRA_fastq_dump_calls.sh
```

In [ ]:

```
find ./ -type d | xargs -I % rm -r %
```

In [ ]:

```

```

### run fastqc and compare to previous cells¶

to assess potential differences in error profiles between datasets

In [ ]:

```

```

#### profile base error rates from sequences¶

Q-scores in order of ASCII characters (from wiki):

In [4]:

```
qscores_sorted = """!"#$%&'()*+,-./0123456789:;<=>?@ABCDEFGHIJKLMNOPQRSTUVWXYZ[\]^_`abcdefghijklmnopqrstuvwxyz{|}~"""
```

In [9]:

```
qscores_sorted.index('J')
```

Out[9]:

```
41
```

In [ ]:

```

```

##### in mouse nasal vasculature¶

write every 4th line (the base quals) from fastq files to a separate file:

In [ ]:

```
ls /mnt/volume/resources/external_SS3_cells/2nd_nasal_dataset_complete_bench/*.gz | cut -c 75- | head -n 2 | xargs -I % sh -c "zcat /mnt/volume/resources/external_SS3_cells/2nd_nasal_dataset_complete_bench/% | sed -n '0~4p' > %"
```

In [ ]:

```

```

we'll sample 150 random cells to limit computation

In [66]:

```
cellnum = 150
```

In [126]:

```
from collections import Counter, defaultdict
import gzip
```

In [379]:

```
def count_letters(file_path, alphabet, seqlen=150):
    alphabet = set(alphabet)

    global_counts = Counter()
    positional_counts = []      # list of Counters, one for each sequence position
    total_lines = 0

    with gzip.open(file_path, mode='r') as f:
        for line in f:
            seq = line.decode("utf-8").strip()
            if not seq or len(seq) <= seqlen:
                continue
            total_lines += 1

            # initialize positional counters on first sequence
            if not positional_counts:
                positional_counts = [Counter() for _ in range(len(seq))]

            # global tally
            global_counts.update(seq)

            # positional tally
            for i, ch in enumerate(seq):
                if ch in alphabet:
                    positional_counts[i][ch] += 1
                    
    return global_counts, positional_counts, total_lines
```

In [141]:

```
def count_letters_cell(cellfile):
    global alphabet
    gcounts, pcounts, n = count_letters(cellfile, alphabet)
    
    posmat = pd.DataFrame(index=range(150), columns=list(alphabet))
    for i in range(150):
        posmat.loc[i] = pcounts[i]

    posmat = posmat.fillna(0).astype('int32')
    return posmat
```

In [ ]:

```

```

In [142]:

```
alphabet = '!"#$%&\'()*+,-./0123456789:;<=>?@ABCDEFGHIJKLMNOPQRSTUVWXYZ[\\]^_`abcdefghijklmnopqrstuvwxyz{|}~'
alphabet
```

Out[142]:

```
'!"#$%&\'()*+,-./0123456789:;<=>?@ABCDEFGHIJKLMNOPQRSTUVWXYZ[\\]^_`abcdefghijklmnopqrstuvwxyz{|}~'
```

In [ ]:

```

```

multithreaded counting of Q-score from each fastq

In [311]:

```
from concurrent.futures import ProcessPoolExecutor

p = "/mnt/volume/analysis/umite_revision_experiments/compare_quality_scores_by_seq"
path = p + "/original_nasal/"
files = pd.Series(os.listdir(path)).sample(cellnum).values

def process(file):
    return count_letters_cell(path + file)

with ProcessPoolExecutor() as pool:
    results = list(pool.map(process, files))
```

In [ ]:

```

```

Q-score counts in a single cell (read position x Q-score code)

In [313]:

```
results[0]
```

Out[313]:

|  | ! | " | # | $ | % | & | ' | ( | ) | \* | ... | u | v | w | x | y | z | { | | | } | ~ |
| --- | --- | --- | --- | --- | --- | --- | --- | --- | --- | --- | --- | --- | --- | --- | --- | --- | --- | --- | --- | --- | --- |
| 0 | 0 | 0 | 0 | 0 | 0 | 0 | 0 | 0 | 0 | 0 | ... | 0 | 0 | 0 | 0 | 0 | 0 | 0 | 0 | 0 | 0 |
| 1 | 0 | 0 | 0 | 0 | 0 | 0 | 0 | 0 | 0 | 0 | ... | 0 | 0 | 0 | 0 | 0 | 0 | 0 | 0 | 0 | 0 |
| 2 | 0 | 0 | 0 | 0 | 0 | 0 | 0 | 0 | 0 | 0 | ... | 0 | 0 | 0 | 0 | 0 | 0 | 0 | 0 | 0 | 0 |
| 3 | 0 | 0 | 0 | 0 | 0 | 0 | 0 | 0 | 0 | 0 | ... | 0 | 0 | 0 | 0 | 0 | 0 | 0 | 0 | 0 | 0 |
| 4 | 0 | 0 | 0 | 0 | 0 | 0 | 0 | 0 | 0 | 0 | ... | 0 | 0 | 0 | 0 | 0 | 0 | 0 | 0 | 0 | 0 |
| ... | ... | ... | ... | ... | ... | ... | ... | ... | ... | ... | ... | ... | ... | ... | ... | ... | ... | ... | ... | ... | ... |
| 145 | 0 | 0 | 0 | 0 | 0 | 0 | 0 | 0 | 252 | 0 | ... | 0 | 0 | 0 | 0 | 0 | 0 | 0 | 0 | 0 | 0 |
| 146 | 0 | 0 | 0 | 0 | 0 | 0 | 0 | 0 | 267 | 0 | ... | 0 | 0 | 0 | 0 | 0 | 0 | 0 | 0 | 0 | 0 |
| 147 | 0 | 0 | 0 | 0 | 0 | 0 | 0 | 0 | 251 | 0 | ... | 0 | 0 | 0 | 0 | 0 | 0 | 0 | 0 | 0 | 0 |
| 148 | 0 | 0 | 0 | 0 | 0 | 0 | 0 | 0 | 286 | 0 | ... | 0 | 0 | 0 | 0 | 0 | 0 | 0 | 0 | 0 | 0 |
| 149 | 0 | 0 | 0 | 0 | 0 | 0 | 0 | 0 | 270 | 0 | ... | 0 | 0 | 0 | 0 | 0 | 0 | 0 | 0 | 0 | 0 |

150 rows × 94 columns

In [314]:

```
files[0]
```

Out[314]:

```
'SRR19886006_2.fastq.gz'
```

In [ ]:

```

```

sum Q-score counts for all cells

In [315]:

```
resmat = pd.DataFrame().reindex_like(results[0]).fillna(0).astype('int64')
readtotal = 0
```

In [316]:

```
for i in results:
    resmat = resmat + i
```

In [317]:

```
resmat = resmat.loc[:, resmat.sum() > 0]
```

In [318]:

```
resmat.columns = [f"Q{alphabet.index(i)}: {i}" for i in resmat.columns]
```

In [319]:

```
resmat
```

Out[319]:

|  | Q2: # | Q8: ) | Q12: - | Q22: 7 | Q27: < | Q32: A | Q37: F | Q41: J |
| --- | --- | --- | --- | --- | --- | --- | --- | --- |
| 0 | 7245 | 0 | 1354563 | 13 | 1190065 | 34944564 | 487 | 16 |
| 1 | 1650 | 0 | 1884519 | 4 | 1503490 | 34106624 | 665 | 1 |
| 2 | 101 | 0 | 1790927 | 627 | 1457845 | 8529078 | 25718375 | 0 |
| 3 | 0 | 0 | 1510171 | 134838 | 1080036 | 3634654 | 31137254 | 0 |
| 4 | 8 | 0 | 1350361 | 267010 | 719858 | 2370582 | 32789134 | 0 |
| ... | ... | ... | ... | ... | ... | ... | ... | ... |
| 145 | 3 | 1400515 | 6569818 | 2316708 | 1292238 | 1703367 | 4003899 | 20210405 |
| 146 | 0 | 1445929 | 6719904 | 2198938 | 1317656 | 1699414 | 4027423 | 20087689 |
| 147 | 0 | 1444225 | 6824252 | 2175821 | 1339189 | 1700391 | 4038248 | 19974827 |
| 148 | 0 | 1461376 | 6999344 | 2086338 | 1340407 | 1709352 | 4092754 | 19807382 |
| 149 | 0 | 1504840 | 7045505 | 2108800 | 1365910 | 1716589 | 4096144 | 19659165 |

150 rows × 8 columns

In [ ]:

```

```

In [323]:

```
f = plt.figure(figsize=(5, 2.5))
ax = f.gca()

plt.plot(resmat[['Q32: A', 'Q37: F', 'Q41: J']].sum(1) / resmat.sum(1))

plt.ylabel('% reads >Q30')
plt.xlabel('position in read')
sns.despine(ax=ax)

plt.tight_layout()
# plt.savefig(figdir + '/', format='svg')
plt.show()
```

In [324]:

```
(resmat[['Q32: A', 'Q37: F', 'Q41: J']].sum(1) / resmat.sum(1))[:50].describe() # first 50 bp of read
```

Out[324]:

```
count   50.000
mean     0.932
std      0.014
min      0.909
25%      0.920
50%      0.934
75%      0.944
max      0.953
dtype: float64
```

In [ ]:

```

```

##### in human T-cell dataset¶

extract quality lines from fastqs

In [ ]:

```
cd /mnt/volume/analysis/umite_revision_experiments/compare_quality_scores_by_seq/second_benchm
```

In [ ]:

```
ls /mnt/volume/analysis/umite_revision_experiments/GEO_SS3_benchmark_dataset/*.gz | cut -c 75- | xargs -I % echo "zcat /mnt/volume/analysis/umite_revision_experiments/GEO_SS3_benchmark_dataset/% | sed -n '0~4p' | gzip > %" > extract_qualities.sh
```

In [ ]:

```

```

In [365]:

```
cellnum
```

Out[365]:

```
150
```

In [380]:

```
path = p + "/second_benchm/"
files = pd.Series(os.listdir(path)).sample(cellnum).values

def process(file):
    return count_letters_cell(path + file)

with ProcessPoolExecutor() as pool:
    results2 = list(pool.map(process, files))
```

In [ ]:

```

```

Q-score counts in T-cell dataset

In [388]:

```
resmat2 = pd.DataFrame().reindex_like(results2[0]).fillna(0).astype('int64')
```

In [389]:

```
for i in results2:
    resmat2 = resmat2 + i
```

In [390]:

```
resmat2 = resmat2.loc[:, resmat2.sum() > 0]
```

In [391]:

```
resmat2.columns = [f"Q{alphabet.index(i)}: {i}" for i in resmat2.columns]
```

In [392]:

```
resmat2
```

Out[392]:

|  | Q2: # | Q11: , | Q12: - | Q25: : | Q26: ; | Q34: C | Q37: F |
| --- | --- | --- | --- | --- | --- | --- | --- |
| 0 | 165191 | 14952223 | 1641177 | 23667685 | 1387919 | 13112843 | 141491869 |
| 1 | 43860 | 13007773 | 1380086 | 17645937 | 1273282 | 13501726 | 149566243 |
| 2 | 671 | 11979368 | 1314405 | 16361444 | 1232103 | 13608647 | 151922269 |
| 3 | 162 | 12289164 | 1218351 | 16334580 | 1137632 | 13799173 | 151639845 |
| 4 | 73273 | 10595830 | 1146376 | 15347215 | 1103133 | 13843413 | 154309667 |
| ... | ... | ... | ... | ... | ... | ... | ... |
| 145 | 13221 | 15777982 | 1557832 | 16164795 | 1405401 | 13191926 | 148307750 |
| 146 | 2050 | 15832545 | 1564715 | 16240500 | 1408228 | 13182216 | 148188653 |
| 147 | 841 | 15751578 | 1588109 | 16188332 | 1425710 | 13141340 | 148322997 |
| 148 | 1459 | 15795946 | 1606548 | 16226372 | 1443428 | 13105183 | 148239971 |
| 149 | 29212 | 15861552 | 1637778 | 16288523 | 1472413 | 13016387 | 148113042 |

150 rows × 7 columns

In [ ]:

```

```

#### effective error rates in umi range¶

recall these matrices are read length x Q-score, summing counts across all cells

In [85]:

```
qresmat = resmat.copy()
qresmat.columns = [int(i[1:].split(':')[0]) for i in resmat.columns]
```

In [86]:

```
qresmat2 = resmat2.copy()
qresmat2.columns = [int(i[1:].split(':')[0]) for i in resmat2.columns]
```

In [ ]:

```

```

sum Q-score values and determine empirical error rate

In [87]:

```
# by chatGPT

def qscore_empirical_error_rate(df):
    total_bases = df.sum().sum()
    
    # compute column sums = total counts per Q
    C_q = df.sum(axis=0)      # Series indexed by Q (int)

    # split HQ vs remainder (Q>=30 vs Q<30)
    hq_mask = C_q.index.astype(int) >= 30
    C_hq = C_q[hq_mask].sum()
    C_rem = C_q[~hq_mask].sum()

    # empirical distribution in remainder
    f_q = (C_q[~hq_mask] / C_rem).fillna(0)

    # convert Q -> error probability
    Q_vals = f_q.index.values.astype(int)
    e_q = 10 ** (-Q_vals / 10.0)
    
    # expected error rate within remainder
    e_rem = (f_q.values * e_q).sum()
    
    # counts of bases
    N_hq = C_hq
    N_rem = C_rem

    # expected errors
    E_hq = N_hq * (10 ** (-30/10.0))
    E_rem = N_rem * e_rem
    E_total = E_hq + E_rem
    
    return E_total / total_bases
```

In [ ]:

```

```

In [88]:

```
qscore_empirical_error_rate(qresmat.loc[0:50]) # empirical error rate among first 50bp of read (by slicing matrix)
```

Out[88]:

```
0.0031072963718039573
```

In [89]:

```
qscore_empirical_error_rate(qresmat2.loc[0:50]) # same for T-cell dataset
```

Out[89]:

```
0.0055707768631960445
```

In [ ]:

```

```

In [90]:

```
f = plt.figure(figsize=(5, 2.5))
ax = f.gca()

plt.plot(resmat[['Q32: A', 'Q37: F', 'Q41: J']].sum(1) / resmat.sum(1), lw=2, label='nasal (mouse)')
plt.plot(resmat2[['Q34: C', 'Q37: F']].sum(1) / resmat2.sum(1), lw=2, label='tcell (human)')

from matplotlib.patches import Rectangle
ax.add_patch(Rectangle((0, 0), 50, 1, color='r', alpha=0.05, ec=None))

ax.text(25, 0.68, "UMI range", ha='center')
ax.text(25, 0.99, "empirical error rate:", ha='center')
ax.text(30, 0.95, f"{qscore_empirical_error_rate(qresmat.loc[0:50]):.4f}", c=plt.get_cmap('tab10')(0))
ax.text(20, 0.82, f"{qscore_empirical_error_rate(qresmat2.loc[0:50]):.4f}", c=plt.get_cmap('tab10')(1))

plt.legend(frameon=False)
ax.set_ylim(0.66, 1)

plt.ylabel('% reads >Q30')
plt.xlabel('position in read')
sns.despine(ax=ax)

plt.tight_layout()
# plt.savefig(figdir + '/', format='svg')
plt.show()
```

In [ ]:

```

```

### compare probability of UMI collision vs sequencing error¶

assuming random UMIs, we can find the probability of collisions at different Hamming thresholds, and otherwise determine the expected number of sequencing errors based on total bases seen (i.e. read depth \* read length)

In [91]:

```
umilen = 8
umialphabet = ['A', 'C', 'G', 'T']
```

In [92]:

```
(len(umialphabet)**umilen)
```

Out[92]:

```
65536
```

In [93]:

```
# exact collision
p_collision = 1 / (len(umialphabet)**umilen)
p_collision
```

Out[93]:

```
1.52587890625e-05
```

In [ ]:

```

```

In [94]:

```
# collision at 1-Hamming: 8 positions, chosen position has 3 options, rest random
p_1ham = umilen * ((len(umialphabet) - 1) / len(umialphabet)) * (1 / (len(umialphabet)) ** (umilen-1))
p_1ham
```

Out[94]:

```
0.0003662109375
```

In [ ]:

```

```

In [95]:

```
# total collision probability (0-Hamming + 1-Hamming)
p_umimatch = p_collision + p_1ham
p_umimatch
```

Out[95]:

```
0.0003814697265625
```

In [ ]:

```

```

#### quantify UMIs per gene¶

here from original nasal dataset, to give an idea what realistic read depths are for quantifying expected number of sequencing errors

In [103]:

```
countsdir = '/mnt/volume/resources/external_SS3_cells/2nd_nasal_dataset_complete_bench/count_tables_800cell'
```

In [104]:

```
countcols = ['UE', 'UI', 'RE', 'RI', 'D']
```

In [105]:

```
mergecounts = {}

for f in tqdm([i for i in os.listdir(countsdir) if i.endswith('.umicounts')]):
    
    cell = f.split('_')[0]
    
    cellcounts = pd.read_csv(countsdir + '/' + f, sep='\t', skiprows=1, header=None, names=['gene'] + countcols)
    mergecounts[cell] = cellcounts.set_index('gene')[['UI', 'UE', 'RE', 'RI', 'D']]
```

In [ ]:

```

```

In [531]:

```
umicounts = {
    'umiE':pd.concat([mergecounts[i]['UE'].rename(i) for i in mergecounts.keys()], axis=1),
    'umiI':pd.concat([mergecounts[i]['UI'].rename(i) for i in mergecounts.keys()], axis=1),
    'intE':pd.concat([mergecounts[i]['RE'].rename(i) for i in mergecounts.keys()], axis=1),
    'intI':pd.concat([mergecounts[i]['RI'].rename(i) for i in mergecounts.keys()], axis=1),
    'dupe':pd.concat([mergecounts[i]['D'].rename(i) for i in mergecounts.keys()], axis=1)
}
```

In [ ]:

```

```

In [541]:

```
ttlcounts = (umicounts['umiE'] + umicounts['intE'] + umicounts['dupe']).iloc[5:]
```

In [545]:

```
ttlcounts
```

Out[545]:

|  | SRR19886053 | SRR19885865 | SRR19885315 | SRR19885276 | SRR19885334 | SRR19886309 | SRR19885707 | SRR19885805 | SRR19885878 | SRR19884996 | ... | SRR19885448 | SRR19885478 | SRR19885470 | SRR19885004 | SRR19886329 | SRR19885803 | SRR19884967 | SRR19885258 | SRR19885591 | SRR19885640 |
| --- | --- | --- | --- | --- | --- | --- | --- | --- | --- | --- | --- | --- | --- | --- | --- | --- | --- | --- | --- | --- | --- |
| gene |  |  |  |  |  |  |  |  |  |  |  |  |  |  |  |  |  |  |  |  |  |
| ENSMUSG00000102693 | 0 | 0 | 0 | 0 | 0 | 0 | 0 | 0 | 0 | 0 | ... | 0 | 0 | 0 | 0 | 0 | 0 | 0 | 0 | 0 | 0 |
| ENSMUSG00000064842 | 0 | 0 | 0 | 0 | 0 | 0 | 0 | 0 | 0 | 0 | ... | 0 | 0 | 0 | 0 | 0 | 0 | 0 | 0 | 0 | 0 |
| ENSMUSG00000051951 | 0 | 0 | 0 | 0 | 0 | 0 | 0 | 0 | 0 | 0 | ... | 0 | 0 | 0 | 0 | 0 | 0 | 0 | 0 | 0 | 0 |
| ENSMUSG00000102851 | 0 | 0 | 0 | 0 | 0 | 0 | 0 | 0 | 0 | 0 | ... | 0 | 0 | 0 | 0 | 0 | 0 | 0 | 0 | 0 | 0 |
| ENSMUSG00000103377 | 0 | 0 | 0 | 0 | 0 | 0 | 0 | 0 | 0 | 0 | ... | 0 | 0 | 0 | 0 | 0 | 0 | 0 | 0 | 0 | 0 |
| ... | ... | ... | ... | ... | ... | ... | ... | ... | ... | ... | ... | ... | ... | ... | ... | ... | ... | ... | ... | ... | ... |
| ENSMUSG00000094431 | 0 | 0 | 0 | 0 | 0 | 0 | 0 | 0 | 0 | 0 | ... | 0 | 0 | 0 | 0 | 0 | 0 | 0 | 0 | 0 | 0 |
| ENSMUSG00000094621 | 0 | 0 | 0 | 0 | 0 | 0 | 0 | 0 | 0 | 0 | ... | 0 | 0 | 0 | 0 | 0 | 0 | 0 | 0 | 0 | 0 |
| ENSMUSG00000098647 | 0 | 0 | 0 | 0 | 0 | 0 | 0 | 0 | 0 | 0 | ... | 0 | 0 | 0 | 0 | 0 | 0 | 0 | 0 | 0 | 0 |
| ENSMUSG00000096730 | 0 | 0 | 0 | 0 | 0 | 0 | 0 | 0 | 0 | 0 | ... | 0 | 0 | 0 | 0 | 0 | 0 | 0 | 0 | 0 | 0 |
| ENSMUSG00000095742 | 0 | 0 | 0 | 0 | 0 | 0 | 0 | 0 | 0 | 0 | ... | 0 | 0 | 0 | 0 | 0 | 0 | 0 | 0 | 0 | 0 |

55536 rows × 800 columns

In [ ]:

```

```

In [546]:

```
ttlcounts = ttlcounts.replace(0, np.NaN)
```

In [552]:

```
fttlcounts = ttlcounts.loc[ttlcounts.shape[1] - ttlcounts.isna().sum(1) > 50] # seen in 50 cells
```

In [556]:

```
fttlcounts.mean(1)
```

Out[556]:

```
gene
ENSMUSG00000025902     6.226
ENSMUSG00000098104     1.387
ENSMUSG00000103922     1.556
ENSMUSG00000033845     9.265
ENSMUSG00000025903     4.378
                       ...  
ENSMUSG00000095320   539.736
ENSMUSG00000079808     2.172
ENSMUSG00000095041     6.562
ENSMUSG00000063897     7.276
ENSMUSG00000095742     1.857
Length: 12281, dtype: float64
```

In [557]:

```
fttlcounts.mean(1).describe()
```

Out[557]:

```
count   12281.000
mean       11.062
std        32.925
min         1.018
25%         5.096
50%         7.186
75%        10.000
max      2107.751
dtype: float64
```

In [ ]:

```

```

In [563]:

```
(fttlcounts.mean(1) < 100).mean() # >99% of genes have <100 reads coverage
```

Out[563]:

```
0.9915316342317401
```

In [ ]:

```

```

plot expected sequencing errors against collisions for realistic coverages

In [107]:

```
seqsets = np.linspace(0, 100, 21)

f = plt.figure(figsize=(5, 3))
ax = f.gca()

xs = range(len(seqsets))

#ax.plot(xs, [p_umimatch*(i - 1) for i in seqsets], label='E[c. per UMI]')
ax.plot(xs, [(p_umimatch*(i - 1)) * (i/2) for i in seqsets], label='E[collisions]')

perr = qscore_empirical_error_rate(qresmat.loc[0:50])
ax.plot(xs, [(1 - (1 - perr)**umilen) * i for i in seqsets], label='nasal: E[UMI err.]')

perr = qscore_empirical_error_rate(qresmat2.loc[0:50])
ax.plot(xs, [(1 - (1 - perr)**umilen) * i for i in seqsets], label='tcell: E[UMI err.]')

ax.set_xticks(xs, [int(i) if i % 10 == 0 else "" for i in seqsets])

ax.set_xlabel('observed UMIs')
#ax.set_ylabel('value')

plt.legend(frameon=False)

sns.despine(ax=ax)

plt.tight_layout()
# plt.savefig(figdir + '/', format='svg')
plt.show()
```

In [ ]:

```

```

In [ ]:

```
# note that in expected number of collisions above, it is assumed that each of the e.g. 100 "observed UMIs" 
# constitute unique sequences. however this is far from the truth as these will mostly 
# contain a small number of UMIs and their duplicates. indeed, in real datasets I expect this value can vary 
# wildly depending on tagmentation level (umi:internal ratio) and depth (duplication level)
```

In [ ]:

```

```

#### quantify umi duplication per dataset¶

by just counting read depth we're overestimating collision rates, so find the global proportion of unique UMIs per dataset to determine an effective number of unique UMIs at a given coverage

In [3]:

```
# first in nasal vasculature dataset
nasal_counts = {i:pd.read_csv('/mnt/volume/analysis/umite_revision_experiments/test_smk_workflow/umite_basic_umite.%s.tsv' %i, sep='\t', index_col=0) for i in countcols}
```

In [ ]:

```

```

In [114]:

```
n_total_umi_reads = nasal_counts['D'] + nasal_counts['UE'] + nasal_counts['UI']
n_total_reads = n_total_umi_reads + nasal_counts['RE'] + nasal_counts['RI']
```

In [ ]:

```

```

In [118]:

```
(nasal_counts['UE'] + nasal_counts['UI']).sum().sum() / n_total_umi_reads.sum().sum() # unique of umi-containing
```

Out[118]:

```
0.3448920782320637
```

In [119]:

```
n_total_umi_reads.sum().sum() / n_total_reads.sum().sum() # umis of total
```

Out[119]:

```
0.45355767252801626
```

In [120]:

```
(nasal_counts['UE'] + nasal_counts['UI']).sum().sum() / n_total_reads.sum().sum() # unique of total
```

Out[120]:

```
0.1564284482762853
```

In [ ]:

```

```

In [121]:

```
n_uoft = (nasal_counts['UE'] + nasal_counts['UI']).sum().sum() / n_total_reads.sum().sum() # unique of total
n_uofu = (nasal_counts['UE'] + nasal_counts['UI']).sum().sum() / n_total_umi_reads.sum().sum() # unique of umi-containing
```

In [ ]:

```

```

In [112]:

```
# then in T-cell dataset
tcell_counts = {i:pd.read_csv('/mnt/volume/analysis/umite_revision_experiments/GEO_SS3_benchmark_dataset/umite_tcell_nofuzzy_umite.%s.tsv' %i, sep='\t', index_col=0) for i in countcols}
```

In [123]:

```
t_total_umi_reads = tcell_counts['D'] + tcell_counts['UE'] + tcell_counts['UI']
t_total_reads = t_total_umi_reads + tcell_counts['RE'] + tcell_counts['RI']
```

In [ ]:

```

```

In [127]:

```
(tcell_counts['UE'] + tcell_counts['UI']).sum().sum() / t_total_umi_reads.sum().sum() # unique of umi-containing
```

Out[127]:

```
0.23025136368855967
```

In [128]:

```
t_total_umi_reads.sum().sum() / t_total_reads.sum().sum() # umis of total
```

Out[128]:

```
0.3706760936302528
```

In [129]:

```
(tcell_counts['UE'] + tcell_counts['UI']).sum().sum() / t_total_reads.sum().sum() # unique of total
```

Out[129]:

```
0.08534867604511392
```

In [ ]:

```

```

In [130]:

```
t_uoft = (tcell_counts['UE'] + tcell_counts['UI']).sum().sum() / t_total_reads.sum().sum() # unique of total
t_uofu = (tcell_counts['UE'] + tcell_counts['UI']).sum().sum() / t_total_umi_reads.sum().sum() # unique of umi-containing
```

In [ ]:

```

```

#### plot combined sequencing errors + collisions¶

In [131]:

```
seqsets = [i for i in range(0, 101, 10)] # range of reasonable coverages
seqsets
```

Out[131]:

```
[0, 10, 20, 30, 40, 50, 60, 70, 80, 90, 100]
```

In [140]:

```
f = plt.figure(figsize=(8, 3))
ax = f.gca()

xs = range(len(seqsets))

# expected from collisions, depth=#UMIs
def ecol(depth):
    return (p_umimatch*(depth - 1)) * (depth/2)

for n, i in enumerate(np.linspace(0, 1, 101)):
    ax.plot(xs, [ecol(float(depth*i)) for depth in seqsets], 
            lw=1, c='k', 
            alpha= (ecol(seqsets[-1] * i) / ecol(seqsets[-1] * 1)) )
    
    if n in [25, 50, 75, 100]:
        e = ecol(seqsets[-1] * i)
        ax.plot((xs[-1] + 0.2, xs[-1] + 0.5), (e, e),
                c='k', lw=0.5)
        
        ax.text(xs[-1] + 0.7, e, f'{n}', fontsize=6, va='center', ha='left')
        
ax.text(xs[-1] + 1.7, ecol(seqsets[-1] * 1) / 2, '%uUMIs at depth', 
    rotation='vertical', va='center', fontsize=7)

# expected from seq errors, depth=depth (umi doesnt matter)
def eseqerr(depth):
    return (1 - (1 - perr)**umilen) * depth

perr = qscore_empirical_error_rate(qresmat.loc[0:50])
ax.plot(xs, [eseqerr(i) for i in seqsets], label='E[seq. errors, nasal dataset]')

perr = qscore_empirical_error_rate(qresmat2.loc[0:50])
ax.plot(xs, [eseqerr(i) for i in seqsets], label='E[seq. errors, tcell dataset]')

ax.plot(xs, [ecol(float(i*n_uofu)) for i in seqsets], 
        label=f'E[collisions, nasal uUMIs: {n_uofu*100:.0f}%]', c='lightcoral')

ax.plot(xs, [ecol(float(i*t_uofu)) for i in seqsets], 
        label=f'E[collisions, tcell uUMIs: {t_uofu*100:.0f}%]', c='r')

ax.set_xticks(xs, [int(i) if i % 10 == 0 else "" for i in seqsets])

ax.set_xlabel('depth aka UMI-containing reads per gene')
ax.set_ylabel('potential corrections')

plt.legend(frameon=False, bbox_to_anchor=[1,1,0,0])

sns.despine(ax=ax)

plt.tight_layout()
# plt.savefig(figdir + '/', format='svg')
plt.show()
```

In [ ]:

```

```

### umite to estimate T-cell UMI gain¶

on GEO tcell dataset to benchmark correction and count fuzzy UMI gains

In [ ]:

```

```

#### track UMI extraction¶

capturing umiextract output

In [ ]:

```
umiextract -1 fastqs/*_1.fastq.gz -2 fastqs/*_2.fastq.gz -d ./fastq_out -c 26 --umilen 8 -a ATTGCGCAATG -t GGG > nofuzz_output.txt
```

In [ ]:

```
umiextract -1 fastqs/*_1.fastq.gz -2 fastqs/*_2.fastq.gz -d ./fastq_out -c 26 --umilen 8 -a ATTGCGCAATG -t GGG -f -s 0 --min_seqlen 30 --anchor_mismatches 2 --anchor_indels 1 --trailing_hamming 1 > yesfuzz_output.txt
```

In [ ]:

```

```

for parsing outputs of umiextract

In [4]:

```
# by chatGPT
import re

pattern = re.compile(
    r'(?P<srr>SRR\d+)_\d+\.fastq\.gz: '
    r'(?P<reads>\d+) reads, '
    r'(?P<umi>\d+) with UMI \((?P<umi_pct>[\d.]+)%\), '
    r'(?P<written>\d+) written \((?P<written_pct>[\d.]+)% skipped\)'
)

def parse_log_line(line):
    m = pattern.search(line)
    if not m:
        return None
    return {
        "SRR": m["srr"],
        "reads": int(m["reads"]),
        "umi": int(m["umi"]),
        "umi_pct": float(m["umi_pct"]),
        "written": int(m["written"]),
        "skipped_pct": float(m["written_pct"]),
    }
```

In [ ]:

```

```

In [5]:

```
nofuzz_stats = {}
with open('/mnt/volume/analysis/umite_revision_experiments/umi_counts_in_GEO_dataset/nofuzz_output.txt', 'r') as f:
    for i in f.readlines():
        stats = parse_log_line(i)
        nofuzz_stats[stats['SRR']] = {i:stats[i] for i in stats if i != 'SRR'}
```

In [6]:

```
nofuzz_stats = pd.DataFrame(nofuzz_stats).T
nofuzz_stats
```

Out[6]:

|  | reads | umi | umi\_pct | written | skipped\_pct |
| --- | --- | --- | --- | --- | --- |
| SRR29626091 | 1597327.000 | 442638.000 | 27.710 | 1597327.000 | 0.000 |
| SRR29625994 | 1831384.000 | 897361.000 | 49.000 | 1831301.000 | 0.000 |
| SRR29627257 | 1698608.000 | 621104.000 | 36.570 | 1698608.000 | 0.000 |
| SRR29624095 | 1814490.000 | 535575.000 | 29.520 | 1814490.000 | 0.000 |
| SRR29626648 | 1884639.000 | 1042806.000 | 55.330 | 1884505.000 | 0.010 |
| ... | ... | ... | ... | ... | ... |
| SRR29625266 | 1649761.000 | 415100.000 | 25.160 | 1648965.000 | 0.050 |
| SRR29626169 | 757898.000 | 167381.000 | 22.080 | 757898.000 | 0.000 |
| SRR29627562 | 707622.000 | 185765.000 | 26.250 | 707622.000 | 0.000 |
| SRR29627871 | 696782.000 | 194924.000 | 27.970 | 696782.000 | 0.000 |
| SRR29628864 | 1014245.000 | 393999.000 | 38.850 | 1014245.000 | 0.000 |

499 rows × 5 columns

In [ ]:

```

```

In [7]:

```
yesfuzz_stats = {}
with open('/mnt/volume/analysis/umite_revision_experiments/umi_counts_in_GEO_dataset/yesfuzz_output.txt', 'r') as f:
    for i in f.readlines():
        stats = parse_log_line(i)
        yesfuzz_stats[stats['SRR']] = {i:stats[i] for i in stats if i != 'SRR'}
```

In [8]:

```
yesfuzz_stats = pd.DataFrame(yesfuzz_stats).T
yesfuzz_stats
```

Out[8]:

|  | reads | umi | umi\_pct | written | skipped\_pct |
| --- | --- | --- | --- | --- | --- |
| SRR29626648 | 1884639.000 | 1122407.000 | 59.560 | 1884129.000 | 0.030 |
| SRR29625994 | 1831384.000 | 972681.000 | 53.110 | 1831061.000 | 0.020 |
| SRR29628506 | 2023167.000 | 1079274.000 | 53.350 | 2022765.000 | 0.020 |
| SRR29627257 | 1698608.000 | 695785.000 | 40.960 | 1698362.000 | 0.010 |
| SRR29625644 | 2859304.000 | 1154128.000 | 40.360 | 2856282.000 | 0.110 |
| ... | ... | ... | ... | ... | ... |
| SRR29625312 | 576841.000 | 131417.000 | 22.780 | 576771.000 | 0.010 |
| SRR29628258 | 668182.000 | 318838.000 | 47.720 | 668141.000 | 0.010 |
| SRR29626169 | 757898.000 | 201588.000 | 26.600 | 757741.000 | 0.020 |
| SRR29627871 | 696782.000 | 227228.000 | 32.610 | 696611.000 | 0.020 |
| SRR29625266 | 1649761.000 | 454807.000 | 27.570 | 1647892.000 | 0.110 |

499 rows × 5 columns

In [ ]:

```

```

In [9]:

```
assert yesfuzz_stats.index.isin(nofuzz_stats.index).all()
```

In [10]:

```
yesfuzz_stats = yesfuzz_stats.reindex(nofuzz_stats.index)
```

In [11]:

```
assert (yesfuzz_stats['reads'] == nofuzz_stats['reads']).all()
```

In [ ]:

```

```

#### umi gain¶

recreating this plot from the manuscript figure (nasal vasculature dataset), now for T-cell dataset

In [ ]:

```

```

In [12]:

```
umiediff = pd.DataFrame()

umiediff['reads'] = yesfuzz_stats['reads']
umiediff['fuzz'] = yesfuzz_stats['umi']
umiediff['nofuzz'] = nofuzz_stats['umi']

umiediff['umigain'] = umiediff['fuzz'] - umiediff['nofuzz']
umiediff['umigain_perc'] = umiediff['umigain'] / umiediff['nofuzz']

umiediff['umiperc'] = umiediff['nofuzz'] / umiediff['reads']

umiediff
```

Out[12]:

|  | reads | fuzz | nofuzz | umigain | umigain\_perc | umiperc |
| --- | --- | --- | --- | --- | --- | --- |
| SRR29626091 | 1597327.000 | 509013.000 | 442638.000 | 66375.000 | 0.150 | 0.277 |
| SRR29625994 | 1831384.000 | 972681.000 | 897361.000 | 75320.000 | 0.084 | 0.490 |
| SRR29627257 | 1698608.000 | 695785.000 | 621104.000 | 74681.000 | 0.120 | 0.366 |
| SRR29624095 | 1814490.000 | 610345.000 | 535575.000 | 74770.000 | 0.140 | 0.295 |
| SRR29626648 | 1884639.000 | 1122407.000 | 1042806.000 | 79601.000 | 0.076 | 0.553 |
| ... | ... | ... | ... | ... | ... | ... |
| SRR29625266 | 1649761.000 | 454807.000 | 415100.000 | 39707.000 | 0.096 | 0.252 |
| SRR29626169 | 757898.000 | 201588.000 | 167381.000 | 34207.000 | 0.204 | 0.221 |
| SRR29627562 | 707622.000 | 220247.000 | 185765.000 | 34482.000 | 0.186 | 0.263 |
| SRR29627871 | 696782.000 | 227228.000 | 194924.000 | 32304.000 | 0.166 | 0.280 |
| SRR29628864 | 1014245.000 | 450615.000 | 393999.000 | 56616.000 | 0.144 | 0.388 |

499 rows × 6 columns

In [ ]:

```

```

In [13]:

```
umiediff['umigain_perc'].describe()
```

Out[13]:

```
count   499.000
mean      0.130
std       0.040
min       0.069
25%       0.103
50%       0.120
75%       0.151
max       0.320
Name: umigain_perc, dtype: float64
```

In [ ]:

```

```

In [14]:

```
f = plt.figure(figsize=(6, 3.5))
ax = f.gca()

p = umiediff[umiediff['reads'] >= 20000].sort_values('umiperc')

sc = plt.scatter(p['umigain_perc'], p['reads'], c=p['umiperc'], 
                 ec='k', lw=0.5, s=60,
                 rasterized=True)


# from matplotlib.patches import Rectangle

# ax.add_patch(Rectangle([mingain, minreads], 
#                        width=maxgain - mingain, 
#                        height=maxreads - minreads,
#                        fill=None, ec='r', lw=3, ls='--'))

# plt.ylim(18e3, 4e6)
# plt.xlim(0.03, 0.23)
plt.yscale('symlog')

plt.xlabel('%UMIs gained by fuzzy matching')
plt.ylabel('Cell library size (#Reads)')

sns.despine(ax=ax)

from mpl_toolkits.axes_grid1.inset_locator import inset_axes
axins = inset_axes(ax, width='3%', height='30%', borderpad=0, loc=3,
                   bbox_to_anchor=(0.89, 0.69, 1, 1), bbox_transform=ax.transAxes)

cb = f.colorbar(sc, cax=axins)
cb.set_label('%UMIs', fontsize=10, rotation=90, labelpad=-10)
cb.set_ticks([p['umiperc'].max(), p['umiperc'].min()])
cb.set_ticklabels([int(round(p['umiperc'].max()*100, 1)), int(round(p['umiperc'].min()*100, 1))])

plt.tight_layout()
#plt.savefig('XX_output/figures/benchmark_v2_scatter_with_cells_noline.svg', format='svg')
plt.show()
```

In [ ]:

```

```

further analzye differences between datasets

In [20]:

```
def extract_umi_stats(text):
    match = re.search(r"^([^:]+): (\d+)\s+reads, (\d+) with UMI.*?, (\d+) written", text)
    return tuple(match.groups()) if match else (None, None, None, None)
```

In [ ]:

```

```

read original umiextract outputs for nasal vasculature dataset

In [21]:

```
nofuzz_orig_stats = pd.DataFrame(columns=['cell', 'reads', 'umis', 'written'])
```

In [22]:

```
with open('/mnt/volume/resources/external_SS3_cells/2nd_nasal_dataset_complete_bench/umicount_umiextract.nofuz.output') as f:
    for n, i in enumerate(f.readlines()):
        if i:
            nofuzz_orig_stats.loc[n] = extract_umi_stats(i)
```

In [23]:

```
nofuzz_orig_stats = nofuzz_orig_stats.set_index('cell').drop(index=[None])
```

In [24]:

```
nofuzz_orig_stats.index = [i.split('_')[0] for i in nofuzz_orig_stats.index]
```

In [25]:

```
nofuzz_orig_stats = nofuzz_orig_stats.astype('int64')
```

In [26]:

```
nofuzz_orig_stats
```

Out[26]:

|  | reads | umis | written |
| --- | --- | --- | --- |
| SRR19885507 | 318840 | 174332 | 318840 |
| SRR19885301 | 322344 | 109011 | 322344 |
| SRR19885158 | 302677 | 172220 | 302677 |
| SRR19885500 | 326264 | 93604 | 326264 |
| SRR19885927 | 318745 | 148780 | 318745 |
| ... | ... | ... | ... |
| SRR19885018 | 1146951 | 752925 | 1146951 |
| SRR19885289 | 1066425 | 552520 | 1066425 |
| SRR19885129 | 1126202 | 552130 | 1126202 |
| SRR19885286 | 1090300 | 673671 | 1090300 |
| SRR19885806 | 1078357 | 688763 | 1078357 |

1423 rows × 3 columns

In [ ]:

```

```

In [27]:

```
yesfuzz_orig_stats = pd.DataFrame(columns=['cell', 'reads', 'umis', 'written'])
```

In [28]:

```
with open('/mnt/volume/resources/external_SS3_cells/2nd_nasal_dataset_complete_bench/umicount_umiextract.yesfuz.output') as f:
    for n, i in enumerate(f.readlines()):
        if i:
            yesfuzz_orig_stats.loc[n] = extract_umi_stats(i)
```

In [29]:

```
yesfuzz_orig_stats = yesfuzz_orig_stats.set_index('cell').drop(index=[None])
```

In [30]:

```
yesfuzz_orig_stats.index = [i.split('_')[0] for i in yesfuzz_orig_stats.index]
```

In [31]:

```
yesfuzz_orig_stats = yesfuzz_orig_stats.astype('int64')
```

In [32]:

```
yesfuzz_orig_stats
```

Out[32]:

|  | reads | umis | written |
| --- | --- | --- | --- |
| SRR19886032 | 276498 | 135813 | 276498 |
| SRR19885578 | 291086 | 114578 | 291086 |
| SRR19885259 | 293474 | 172989 | 293474 |
| SRR19886087 | 286659 | 154886 | 286659 |
| SRR19886281 | 295610 | 164063 | 295610 |
| ... | ... | ... | ... |
| SRR19885146 | 1331512 | 710895 | 1331512 |
| SRR19885216 | 1307853 | 799305 | 1307853 |
| SRR19885150 | 1318194 | 784403 | 1318194 |
| SRR19885522 | 1295670 | 745206 | 1295670 |
| SRR19885297 | 1255936 | 655213 | 1255936 |

1440 rows × 3 columns

In [ ]:

```

```

In [33]:

```
yesfuzz_orig_stats = yesfuzz_orig_stats.reindex(nofuzz_orig_stats.index).dropna()
```

In [34]:

```
assert ((yesfuzz_orig_stats['reads'] - nofuzz_orig_stats['reads']) == 0).all()
```

In [35]:

```
assert ((yesfuzz_orig_stats['written'] - nofuzz_orig_stats['written']) <= 1).all()
```

In [ ]:

```

```

In [36]:

```
umiediff_orig = pd.DataFrame()
```

In [37]:

```
umiediff_orig['reads'] = yesfuzz_orig_stats['reads']
umiediff_orig['fuzz'] = yesfuzz_orig_stats['umis']
umiediff_orig['nofuzz'] = nofuzz_orig_stats['umis']
```

In [38]:

```
umiediff_orig['umigain'] = umiediff_orig['fuzz'] - umiediff_orig['nofuzz']
umiediff_orig['umigain_perc'] = umiediff_orig['umigain'] / umiediff_orig['nofuzz']
```

In [39]:

```
umiediff_orig['umiperc'] = umiediff_orig['nofuzz'] / umiediff_orig['reads']
```

In [41]:

```
umiediff_orig
```

Out[41]:

|  | reads | fuzz | nofuzz | umigain | umigain\_perc | umiperc |
| --- | --- | --- | --- | --- | --- | --- |
| SRR19885507 | 318840 | 183696 | 174332 | 9364 | 0.054 | 0.547 |
| SRR19885301 | 322344 | 115644 | 109011 | 6633 | 0.061 | 0.338 |
| SRR19885158 | 302677 | 181537 | 172220 | 9317 | 0.054 | 0.569 |
| SRR19885500 | 326264 | 100494 | 93604 | 6890 | 0.074 | 0.287 |
| SRR19885927 | 318745 | 158181 | 148780 | 9401 | 0.063 | 0.467 |
| ... | ... | ... | ... | ... | ... | ... |
| SRR19885018 | 1146951 | 796550 | 752925 | 43625 | 0.058 | 0.656 |
| SRR19885289 | 1066425 | 579159 | 552520 | 26639 | 0.048 | 0.518 |
| SRR19885129 | 1126202 | 582017 | 552130 | 29887 | 0.054 | 0.490 |
| SRR19885286 | 1090300 | 703888 | 673671 | 30217 | 0.045 | 0.618 |
| SRR19885806 | 1078357 | 723999 | 688763 | 35236 | 0.051 | 0.639 |

1423 rows × 6 columns

In [ ]:

```

```

begin comparison

In [73]:

```
umiediff_orig['umigain_perc'].describe()
```

Out[73]:

```
count   1423.000
mean       0.079
std        0.027
min        0.042
25%        0.061
50%        0.071
75%        0.090
max        0.403
Name: umigain_perc, dtype: float64
```

In [72]:

```
umiediff['umigain_perc'].describe()
```

Out[72]:

```
count   499.000
mean      0.130
std       0.040
min       0.069
25%       0.103
50%       0.120
75%       0.151
max       0.320
Name: umigain_perc, dtype: float64
```

In [ ]:

```

```

In [71]:

```
f = plt.figure(figsize=(3.5, 2))
ax = f.gca()

sns.kdeplot(umiediff_orig['umigain_perc'], ax=ax, label='nasal (mouse)')
sns.kdeplot(umiediff['umigain_perc'], ax=ax, label='tcell (human)')

plt.legend(frameon=False)

sns.despine(ax=ax)

plt.xlabel('%UMIs gained by fuzzy matching')

plt.tight_layout()
# plt.savefig(figdir + '/', format='svg')
plt.show()
```

In [ ]:

```

```

In [70]:

```
f = plt.figure(figsize=(3.5, 2))
ax = f.gca()

sns.kdeplot(umiediff_orig['umiperc'], ax=ax, label='nasal (mouse)')
sns.kdeplot(umiediff['umiperc'], ax=ax, label='tcell (human)')

#plt.legend(frameon=False)

sns.despine(ax=ax)

plt.xlabel('%UMIs')

plt.tight_layout()
# plt.savefig(figdir + '/', format='svg')
plt.show()
```

In [ ]:

```

```

In [ ]:

```

```
