# Supplementary material for: umite: fast quantification of Smart-seq3 libraries with improved UMI retrieval
Source: Bioinformatics. 2026 Feb 15;42(3):btag075. doi: 10.1093/bioinformatics/btag075 (PMC12989134; doi:10.1093/bioinformatics/btag075)
Supplement: btag075_Supplementary_Data [file btag075_supplementary_data.zip › umite_algorithm_details_word_conversion.pdf]

Algorithmic details

This document serves to outline key algorithmic details of the two umite tools umiextract and umicount.

Fuzzy UMI matching in umiextract

The central aim of umiextract is to identify UMI-containing reads based on the presence of anchor & trailing sequences flanking a putative UMI. The main problem then is to determine whether a read contains sequences which sufficiently resemble the anchor/trailing sequences to enable UMI detection. Among existing tools including zUMIs, this determination is made based on exact matches of the pre-defined anchor & trailing sequences to the read sequence, whereas umiextract uses fuzzy (i.e. approximate) string matching to allow for a small number of indels (i.e. likely sequencing errors) in this case. Thus, fuzzy UMI matching simply describes a procedure where, rather than every character of the anchor & trailing sequences having to be identical and in the same position, a limited number of errors are tolerated in UMI detection. How this is achieved algorithmically and the associated tunable parameters are described below.

Fuzzy matching details

In umiextract, fuzzy string matching is implemented using the python `regex` library (https://github.com/mrabarnett/mrab-regex). This module implements a regex engine in C which enables approximate pattern matches through a qualifier like: `(pattern){e<=2,i<=1,d<=1}`, where `e<=2` sets a maximum of two of any type of error, while limits for insertions (`i`), deletions (`d`), and substitutions (`s`) are denoted separately. Different weights (i.e. edit costs) are able to be specified for these quantities, though this feature is not used in umiextract. To apply fuzzy UMI matching, umiextract precompiles a regex while specifying `{e<={anchor_max_mismatch + anchor_max_indel}}`, where `anchor_max_mismatch` and `anchor_max_indel` are derived from command-line arguments `--anchor_mismatches` and `--anchor_indels` with default values of 2 and 1, respectively. The entire regex construction is as follows:

```
    anchor_fuzzy = rf"({anchor_seq}){{e<={anchor_max_mismatch + anchor_max_indel}}}"
    umi_capture = rf"([ACGTN]{{{umi_len}}})"
    fuzzy_pattern = regex.compile(anchor_fuzzy + umi_capture, flags=regex.BESTMATCH)
```

Here the anchor sequence is specified via `--anchor` on the command line, and the trailing sequence via `--trailing`. The trailing sequence is not included in the `fuzzy_pattern`, as this is checked later once a match has been identified. In the case of a hit, the trailing sequence is determined by extracting the next `len(trailing_seq)` bases after the match. As the resulting sequence match object reports the number of substitutions, insertions, and deletions separately, these are checked against the user-specified `anchor_max_mismatch` and `anchor_max_indel` counts. Additionally, if the Hamming distance (using `RapidFuzz.Hamming`) between the `trailing_seq` and the extracted trailing sequence is also lower than the `trailing_dist_thresh` (defined on the command line by --

trailing_hamming_threshold and with default value 2), the match is determined to contain a bona-fide UMI.

To summarize: first, a regex pattern is defined which matches the anchor sequence with max anchor_max_mismatch plus anchor_max_indel errors, and is combined with a pattern comprising umi_len (via --umilen) random bases that will be used to extract the UMI. R1 sequences are searched against this regex and if a match is found, the trailing sequence is extracted by extending it len(trailing_seq) additional bases. Given a hit, the number of anchor-sequence mismatches and indels, along with the trailing-sequence mismatches are compared to the user-defined thresholds, before extracting the UMI sequence. Note that this fuzzy matching procedure is initiated only if --fuzzy_umi is specified on the command line (or also if any of --anchor_mismatches, --anchor_indels, or --trailing_hamming_threshold are set).

Algorithm overview

Cells are handled independently in umiextract, enabling safe multithreading. For each cell's FASTQs, each read is processed by first searching against a simple, exact-match regex to enable rapid detection of error-less UMIs. If no such match is detected, fuzzy UMI matching is employed as described above. Here, a performance optimization is available via the --search_region parameter which limits where the pattern can be found: for example, by specifying --search_region 30, only the first 30bp of the read will be searched for anchor and trailing sequence hits, saving resources by processing fewer bases overall. If a UMI is found, the anchor, UMI, and trailing sequences are trimmed from the read, and the UMI appended to the read name. If no UMI was found and the user has specified --only_umi, that read is skipped. Once trimmed, the length of the remaining read sequence is checked against the --min_seqlen parameter, such that the use of e.g. --min_seqlen 50 would ensure at least 50bp remain for downstream genome alignment. Finally, if R2 is supplied and a UMI was found for R1, that UMI is also appended to the R2 read name. As each of these reads is processed, the individual R1/R2 FASTQ entries are written to a gzipped output file on the fly. Each read's name is additionally tracked using efficient set operations, such that duplicated read names can be reported.

umiextract command line arguments

**Flag**	**Description**	**Default**
-1 / --read1	*R1* FASTQ files (space-separated)	*required*
-2 / --read2	*R2* FASTQ files (same order as R1)	–
-d / --output_dir	Where to write processed FASTQs	.
-c / --cores	Parallel workers (one cell per core)	4
-l / --logfile	Path to log file	sys.stdout

Flag	Description	Default
`--umilen`	UMI length in bp	`8`
`--anchor`	Pre-UMI anchor (TSO) sequence	`ATTGCGCAATG`
`--trailing`	Post-UMI trailing sequence	`GGG`
`--search_region`	Sequence cutoff to search for UMI	`-1` (whole read)
`--fuzzy_umi`	Enable mismatch/indel-tolerant UMI detection	off
`--anchor_mismatches`	Max mismatches allowed to find anchor match	`2`
`--anchor_indels`	Max indels allowed to find anchor match	`1`
`--trailing_hamming_threshold`	Max Hamming distance allowed in trailing	`2`
`--min_seqlen`	Minimum remaining sequence after trimming UMI	`-1`
`--only_umi`	Drop reads that lack a detectable UMI	off

UMI deduplication and correction in umicount

In umicount, a bam-file containing genome-aligned reads pre-processed with umiextract are checked against the corresponding genome annotation to determine which reads overlap genomic features, and the counts of corrected and deduplicated UMIs as well as internal reads reported. This requires distinct functionalities, including assigning reads to genomic features, deduplicating reads by UMI, correcting and merging similar UMIs based on read support, and reporting gene counts. These individual steps and the associated tunable parameters are described below.

Assigning reads to genomic features

Quantifying gene expression requires linking genomic coordinates to genomic features. These coordinates are determined through genome alignment and are available in the BAM files which serve as input to umicount, while the coordinates of genomic features are retrieved from established databases which record them in GTF format. To link these two sets of coordinates (reads and features), we follow the set-based strategy implemented in htseq-count. Thus, each record is parsed from the user-supplied GTF file (`--gtf`) and the coordinates (e.g. of an exon, gene, transcript, or miRNA, etc.) recorded in a `HTSeq.GenomicArrayOfSets` while relevant

gene- and exon-level attributes are recorded in bespoke dictionaries. Once all GTF records have been parsed, the genomic features overlapping any genomic interval can be efficiently queried by slicing the `HTSeq.GenomicArrayOfSets` with a `HTSeq.GenomicInterval`. In umicount, this is used to determine genomic features overlapping the aligned position of a given read, with additional logic to distinguish intergenic from intronic and exonic features. Ultimately, reads are assigned to one of the following categories, with only `UNIQUE` reads recorded as actual gene counts in the umicount output:

```
    UNIQUE        = "_unique"         # exactly one alignment, counted
    UNMAPPED      = "_unmapped"       # no valid aligned readpairs
    MULTIMAPPING  =  "_multimapping"    #  more  than  one  valid  aligned
readpair
    NO_FEATURE      =  "_no_feature"        #  readpair  alignments  to
intergenic regions
    AMBIGUOUS     = "_ambiguous"      # conflicting gene annotation for
readpair
```

These categories are assigned based on genomic features overlapping a read (described in `ReadTrack.evaluate_overlap()`). If paired-end data are available and both reads overlap exons of a given gene, that gene takes precedence over other overlapping genes without shared exon hits; otherwise, all overlapping genes are recorded and the read is assigned as `AMBIGUOUS` unless only one of the overlapping hits is exonic (and the rest intronic). If no overlapping features exist, a read is assigned `NO_FEATURE`. Likewise, if either read has no alignment reported, a read is considered `UNMAPPED`. Assignment of the `MULTIMAPPING` category depends on how primary alignments are counted. BAM files are sorted by read name prior to running umicount, placing multiple read alignments in adjacent lines. These are read together and overlapping genomic features for each alignment determined as above. Generally, reads reporting multiple alignments overlapping different genes will be assigned as `MULTIMAPPING`, unless the user specifies `--count_primary`, in which case the alignment reported as primary (typically the highest-scoring alignment) will be assigned as `UNIQUE` and counted. However, some aligners have different ways to handle equally-scoring alignments, for example choosing a random alignment as primary, or reporting all best-scoring alignments as primary. In the latter case, these umicount implements several options which depend on the `--multiple_primary_action` command-line argument. This can take values of `warn`, `raise`, or `skip`: if set to warn, a warning is printed and a random primary alignment chosen; if set to raise, an error is raised; and if set to skip, the read is considered `MULTIMAPPING`. This way, all reads are assigned categories according to their genomic overlap, which determines which reads are eligible for downstream processing into umicount count tables.

Of note, as GTF parsing can take several minutes, umicount implements the option to parse from the GTF file once and dump the contents to a `pickle` file. Using `--gtf` with `--GTF_dump` will enable dumping parsed GTF data to a `pickle` file which can be used as input for `umicount` with `--GTF_skip_parse` instead of `--gtf`. This functionality is useful when running multiple repeat quantifications, however generally `--gtf` is the better option. Here a minimal example of this functionality:

```
umicount \
    -gtf examplefile.gtf \
    --GTF_dump umite_GTF_dump.pkl
```

```
umicount \
    --bams example.bam
    --GTF_skip_parse umite_GTF_dump.pkl
```

Deduplicating, correcting, and merging UMIs

With typical sequencing depths of ~100k reads, the 65,536 unique combinations of the random 8bp UMIs which are default in Smart-seq3 are rapidly exhausted and likely to lead to unintended collisions. This is avoided in umite and similar tools by conducting UMI operations in umicount on a per-gene basis. Thus, before deduplication, correction, merging, and counting can begin, all reads must be assigned to their respective overlapping genes, if possible (described above). In umicount, this is handled using python dictionaries indexed by gene and tracking the number of times each UMI (or non-UMI read) was observed, enabling UMI deduplication on the fly. With the command-line option `--no_dedup`, this can be disabled, whereupon the total UMI counts of each gene are output instead of only the unique UMIs.

By specifying `--UMI_correct`, the user can enable UMI correction in umicount. In this case, the set of UMIs corresponding to a given gene are subject to directional Hamming-distance-based merging as in zUMIs and UMI-tools. Briefly, UMI correction operates on a dictionary indexed by unique UMIs, with values tracking the number of times each UMI was observed. For UMI correction and merging, we want to identify UMIs whose Hamming distance is lower than the user-supplied `--hamming_threshold` (default 1), where one UMI has at least 2-times the read-support the other UMI has. This level of read support can be modified by the command-line argument `--count_ratio_threshold`, though both zUMIs and UMI-tools (and umicount) default to a value of 2. The aim of this directed correction is to rule out the collapse of a high-support UMI into a low-support UMI, but promote the vice-versa of merging low-support UMIs into high-support ones. This approach has the added benefit of limiting chain-merging of UMIs (where two 1-Hamming UMIs are merged and subsequently merged into another 1-Hamming neighbor, although one of the original UMIs had a Hamming distance of 2 to the second merge candidate). Practically, UMI correction and merging are implemented by sorting the gene UMI dictionary by decreasing counts, and looping over low-support UMIs to find candidate merges until no further merges are possible according to the directional constraint. The corrected and merged UMI counts returned this way are the final values output by umicount.

Algorithm overview

As in umiextract, cells are handled independently in umicount, enabling safe multithreading. In each instance, the user-supplied GTF file (`--gtf` or `--GTF_skip_parse`, see above) is parsed and bundled reads in the read name sorted BAM file iterated over to determine overlapping genomic features and set read categories. UMI-containing and non-UMI reads are distinguished based on the presence of the UMI in the read name (strictly based on a _ which is included in umiextract). Optionally, reads with a reported mapQ value lower than supplied by `--min_read_mapQ` (default 0) are excluded prior to determining genomic feature overlap. Once assigned to genes, UMIs are deduplicated, corrected, and merged, and finally written cell-by-cell into a gene x cell output matrix.

Of note, intronic and exonic gene counts are distinguished as outlined above, being categorized as UMI-containing exonic (`UE`), UMI-containing intronic (`UI`), non-UMI exonic (`RE`), non-UMI

intronic (`RI`), and UMI-duplicate (`D`) by default. These categories each produce an individual output file collating that category's counts across all processed cells. UMI-containing and non-UMI reads are always distinguished, however whether intronic and exonic reads are treated separately or handled together depends on whether the user supplied `--combine_unspliced` or not. If set, `--combine_unspliced` will reduced the tracked categories to `U`, `R`, and `D` only. Also, the `D` category will be excluded if `--no_dedup` is set.

umicount command line arguments

Flag	Description	Default
`-f / --bams`	Read-name–sorted BAM files, 1 per cell	*required*
`-d / --output_dir`	Output directory	`.`
`-c / --cores`	Parallel workers (one BAM per core)	all cores
`-l / --logfile`	Path to log file	`sys.stdout`
`-g / --gtf`	Ensembl-style GTF annotation	*required**
`--tmp_dir`	Directory to save temporary files	`--output_dir`
`--no_dedup`	Skip deduplication and report all UMI-reads	off
`--mm_count_primary`	Count primary alignment for multimapping reads	off
`--multiple_primary_action`	When a read has mutliple primary alignments: `warn`, `raise`, or `skip`	`warn`
`--min_read_mapQ`	Min mapQ to keep read	`0`
`--combine_unspliced`	If set, don't distinguish intronic and exonic reads	off
`--UMI_correct`	Enable gene-wise UMI collapse by Hamming distance	off
`--hamming_threshold`	Hamming threshold for merging UMIs	`1`
`--count_ratio_threshold`	Only merge UMIs if one has ((threhsold-times)+1) as many counts	`2`

* - use of `--gtf` is required unless specifying a pickle file via `--GTF_skip_parse`, see above.
