# Supplementary material for: umite: fast quantification of Smart-seq3 libraries with improved UMI retrieval
Source: Bioinformatics. 2026 Feb 15;42(3):btag075. doi: 10.1093/bioinformatics/btag075 (PMC12989134; doi:10.1093/bioinformatics/btag075)
Supplement: btag075_Supplementary_Data [file btag075_supplementary_data.zip › umite_workflow_details_word_conversion.pdf]

umite snakemake workflow

This folder contains a Snakemake workflow designed to enable portable and reproducible quantification of Smart-Seq3 libraries with umite.

Requirements

umite quantification requires as input:

- FASTQ file(s) per cell,
- a reference genome in Fasta format (e.g. from Ensembl),
- and a reference genome annotation in GTF format (e.g. from Ensembl).

Both paired-end and single-end FASTQ files are supported. For other input options see the Configuration section below.

Software requirements for the umite pipline include only Snakemake and conda: all other required software will be downloaded and locally installed via Snakemake's integrated package management. These packages are defined in `umite_conda.yaml` and include `STAR`, `samtools`, `pandas`, and `umite` (which extends `HTSeq`, `RapidFuzz`, and `regex`).

Configuration

Prior to running the workflow, you will need to update the configuration file `snakeconfig.yaml` to specify required inputs as well as optional parameters. These include a basic `runID`, which is used to prefix and uniquely name output files, as well as an `output_dir` and `log_dir` for writing output- and log-files, respectively. The number of threads (cores) to use in umite and STAR are defined under `threads`. A reference `genome` and `annotation` file in Fasta and GTF format are also required.

Importantly, input FASTQ files are defined via a `samples_file`, which is expected to contain one sample name per line, such that the concatenation of the sample name and the `R1/R2_suffix` point to an existing R1/R2 FASTQ file in the `fastq_dir` each. For example, `samples_file` could be `./sample_names.txt`, with `fastq_dir` defined as `./fastqs`, while `R1_suffix` is `"_R1.fastq.gz"` and `R2_suffix` is `"_R2.fastq.gz"`. Let's assume sample_names.txt contains the following samples:

```
control_cell1
treated_cell1
treated_cell2
```

then it is expected that the files `./fastqs/control_cell1_R1.fastq.gz` and `./fastqs/control_cell1_R2.fastq.gz` exist, and these will be used to quantify paired-

end read counts for the `control_cell1` sample. The `treated_cell1` and `treated_cell2` samples will be likewise expanded and have their counts quantified.

Other parameters for `umiextract` and `umicount` in this workflow are set in this configuration file and described in greater detail in the umite `README.md`. Of note are the `anchor_seq`, `trailing_seq`, and `umilen` parameters, which are essential to the accurate detection of UMIs. As in the original Smart-seq3 publication, these values default to `"ATTGCGCAATG"`, `"GGG"`, and `8`, respectively. Fuzzy UMI matching is enabled by default in this workflow and can be disabled by setting `fuzzy_umi: False`. Similarly, UMI correction (i.e. merging) can be disabled by setting `correct_umis: False`.

The extra program arguments (`extra_args`) parameters comprise strings of command-line options which are appended to the specified programs when executed.

Execution

To run the umite snakemake workflow, first ensure that the required data are input in `snakeconfig.yaml` and that `Snakemake` and `conda` (or `miniconda`) are installed. Then, the workflow can be initiated by calling:

```
snakemake --sdm conda -s snakefile_umite_star.smk
```

This will read input files from the `samples_file` defined in `snakeconfig.yaml`, conduct UMI detection, alignment to the genome reference, and read count quantification as described in the umite manuscript (see below for more details). Output and log files are written to the `output_dir` and `log_dir` specified in `snakeconfig.yaml`, respectively.

Outputs

umite produces individual output matrices to track gene x cell counts for UMI-containing (`U`) and non-UMI internal (`R`) reads. Intronic (`I`) and exonic (`E`) reads will likewise be distinguished unless `combine_unspliced: True` is set in `snakeconfig.yaml`. Duplicated UMIs (`D`) will likewise be output separately unless `dedupe_umis: False` is specified. Assuming default behavior and a `runID` specified in `snakeconfig.yaml`, the following gene x cell matrices are created:

```
{runID}_umite.UE.tsv # exonic UMI counts
{runID}_umite.UI.tsv # intronic UMI counts
{runID}_umite.RE.tsv # exonic non-UMI counts
{runID}_umite.RI.tsv # intronic non-UMI counts
{runID}_umite.D.tsv  # UMI duplicate counts
```
